# *Clostridium butyricum* alleviates multiple myeloma by remodeling the bone marrow microenvironment and inhibiting PI3K/AKT pathway through the gut‒bone axis

**DOI:** 10.1080/19490976.2025.2609455

**Published:** 2026-01-02

**Authors:** Jingyu Wang, Fuming Zi, Wu Liu, Chengrui Liu, Zhengfeng Zhang, Leilei Kong, Xuan Xu, Jing Wei, Tingtao Chen, Jian Li

**Affiliations:** a The Department of Hematology, The Second Affiliated Hospital, Jiangxi Medical College, Nanchang University, Nanchang, China; b Jiangxi Province Key Laboratory of Bioengineering Drugs, School of Pharmacy, Jiangxi Medical College, Nanchang University, Nanchang, China; c The Department of Cardiovascular Surgery, The Second Affiliated Hospital, Jiangxi Medical College, Nanchang University, Nanchang, China; d Queen Mary School, Jiangxi Medical College, Nanchang University, Nanchang, China; e Huankui Academy, Jiangxi Medical College, Nanchang University, Nanchang, China; f National Engineering Research Center for Bioengineering Drugs and the Technologies, Institute of Translational Medicine, Jiangxi Medical College, Nanchang University, Nanchang, China; g Jiangxi Provincial Key Laboratory of Hematological Diseases, The Second Affiliated Hospital, Jiangxi Medical College, Nanchang University, Nanchang, China

**Keywords:** Multiple myeloma, myeloma bone disease, gut microbiota, bone marrow microenvironment, PI3K/AKT pathway

## Abstract

Emerging evidence reveals a strong connection between the gut microbiota and cancer. However, the exact role of gut microbiota dysbiosis in multiple myeloma (MM) is poorly understood, and the therapeutic potential of microbiota-targeted interventions represents a promising strategy that demands urgent mechanistic and translational investigation. First, we conducted a comprehensive microbiome-metabolite analysis between MM patients and healthy individuals. The result revealed a marked compositional difference characterized by reduced abundances of butyrate-producing bacteria and diminished butyrate levels in the MM cohort. Subsequent fecal microbiota transplantation demonstrated that the gut microbiota critically modulates MM progression, with healthy donor-derived microbiota reducing the tumor burden and concomitantly elevating serum butyrate. Furthermore, through function-based culturomics screening, *Clostridium butyricum* (*C. butyricum*) was identified as a key butyrate-producing specialist. *C. butyricum* or its metabolite butyrate significantly reduced the systemic tumor burden in 5TGM1 mice. Notably, *C. butyricum* and butyrate alleviated bone marrow inflammation and osteolytic lesions by suppressing Th17 cells and IL-17 levels in the bone marrow. Moreover, cellular assays and transcriptome sequencing further revealed that butyrate could induce MM cells' apoptosis via HDAC inhibition-mediated upregulation of PPARγ, leading to sequential suppression of the PI3K/AKT pathway and antiapoptotic BCL-2 expression. This apoptotic signaling cascade was reversed by PPARγ antagonism. The direct antitumor effect was further confirmed in M-NSG mice. Our research systematically verifies the specific role of the gut microbiota in MM and provides the first evidence of the immune and molecular mechanisms by which *C. butyricum* alleviates MM progression, offering preclinical support for probiotic-based therapies against MM.

## 1. Introduction

Multiple myeloma (MM) is the second most common hematological malignancy and has shown an increasing trend in incidence globally with aging and improved diagnostic techniques.^1^ Despite significant advances in treatment, MM remains the leading cause of death among hematological cancers, contributing to more than 100,000 deaths annually.^2^ Characterized by the massive proliferation of malignant plasma cells in the bone marrow and the accumulation of monoclonal immunoglobulins, MM frequently leads to complications such as anemia, bone destruction, and renal insufficiency, severely affecting the patients' quality of life and survival.^3^ Currently, induction therapy based on proteasome inhibitors (PIs), immunomodulatory drugs (IMiDs), and dexamethasone followed by autologous hematopoietic stem cell transplantation remains the mainstay of treatment for MM.^4^ Although next-generation drugs and immunotherapies have broadened therapeutic options, MM remains incurable due to relapse and drug resistance. Thus, identifying novel therapeutic targets is critical for improving MM outcomes.

The pathogenesis of MM is complex and highly heterogeneous, primarily associated with genetic alterations in plasma cells and adaptations in the bone marrow microenvironment (BMM), which provides a permissive niche for malignant plasma cells.^5^ The disorder of BMM is defined by persistent inflammation.^6^ Interactions among immune cells, stromal cells, and plasma cells drive the production of inflammatory mediators such as IL-6, TNF-α, and IL-1β. These mediators promote MM progression and drug resistance by activating pro-survival signaling pathways, notably the frequently upregulated PI3K/AKT pathway.^7^ AKT suppresses autophagy and enhances tumor metabolism by activating mTOR via phosphorylation of the TSC1/2 complex, while phosphorylating pro-apoptotic factors BAD and BAX to inhibit apoptosis.^8^ Furthermore, crosstalk between PI3K/AKT and other pathways (e.g., NF-κB and MAPK) amplifies its pro-tumorigenic effects.^8^ Beyond inflammation, immune dysregulation in the BMM directly facilitates MM immune evasion.^7^ Elevated IL-6 and TGF-β suppress CD4^+^ T-cell proliferation, expand the Th17 population, and promote secretion of IL-17 and IL-10, which collectively impair immune surveillance and exacerbate bone destruction (myeloma bone disease, MBD).^9^ Critically, even successful anti-tumor therapies often fail to fully resolve this pathogenic inflammation, underscoring the need for novel strategies targeting dysregulated microenvironment.^6^

Risk factors for MM may include age, obesity, chronic inflammation, chemical exposures, genetic predisposition, and environmental triggers, but their exact contributions remain incompletely defined.^3^ Recent studies suggest that gut microbiota dysbiosis influences tumorigenesis and therapeutic responses through diverse mechanisms. For instance, bacterial-derived lipopolysaccharide promotes intestinal lymphoma progression via the TLR4/MyD88/NF-κB pathway.^10^ Importantly, the gut microbiota can regulate systemic host metabolism and immune homeostasis, thereby contributing to pathological processes even at extraintestinal sites. Zhou et al. reported that the enrichment of nitrogen-cycling bacteria accelerates MM progression through elevated circulating urea nitrogen.^11^ Similarly, *Citrobacter freundii* exacerbates drug resistance by increasing serum ammonium levels, suggesting that the microbiota as a potential therapeutic target.^12^ *Clostridium butyricum*, a commensal bacterium producing short-chain fatty acids (SCFAs) via dietary fiber fermentation, demonstrates protective roles in multiple pathologies.^13^ It suppresses colon cancer by modulating bile acid metabolism, inhibiting the Wnt/β-catenin pathway, and sensitizes pancreatic cancer cells to ferroptosis through hypoxia-induced lipid peroxidation.^14^ ^,^ ^15^ SCFAs can also improve adoptive immunotherapy in melanoma and pancreatic cancer models by modulating CD8^+^ T cell responses.^16^ However, its role in MM and the underlying mechanisms remain unexplored.

In this study, we conducted a comprehensive analysis of fecal microbiome-metabolite data from a clinical cohort to investigate the relationship between the gut microbiota and MM. Our results revealed a maladjusted gut microbiota feature in MM patients compared with healthy individuals, notably marked by a significant reduction in the abundance of butyrate producers. Furthermore, we evaluated the effects of *C. butyricum* and its metabolite butyrate on the development of MM through a series of *in vivo* and *in vitro* experiments. Our findings demonstrated that *C. butyricum* and butyrate can inhibit the accumulation of Th17 cells in the bone marrow, thereby modulating the BMM. In addition, butyrate promotes MM cell apoptosis directly by activating PPARγ to suppress the PI3K-AKT signaling pathway. Overall, this research provides a novel insight into the mechanism of the gut‒bone axis and identifies a promising auxiliary therapeutic strategy targeting the gut microbiota in MM management.

## 2. Materials and methods

### 2.1. Human subject

The clinical cohort was recruited at the Second Affiliated Hospital of Nanchang University and consisted of 30 newly diagnosed MM patients and 30 age- and gender-matched healthy volunteers. All patients met the diagnostic criteria in the Chinese Guidelines for the Diagnosis and Treatment of MM (Revised 2022). Patients with relapsed/refractory MM or M protein types of IgD, IgM, or IgE were excluded. Other exclusion criteria included: comorbid gastrointestinal and other systemic diseases; use of antibiotics, probiotics, or other medications that may affect the gut microbiota within three months. Furthermore, imaging evaluations, including plain X-ray, CT, MRI, and PET-CT, were performed for all MM patients at the time of diagnosis. According to the 2014 International Myeloma Working Group (IMWG) elaboration on MBD and the actual clinical workup, MBD was defined as the presence of one or more sites of osteolytic bone destruction (≥5 mm in size) seen on bone radiography. High-risk cytogenetic abnormalities included del(17/17p), t(4;14), t(14;16), t(14;20), and gain 1q. All procedures were reviewed and approved by the Medical Ethics Committee of the Second Affiliated Hospital of Nanchang University (Approval No. 2023092). All participants signed the informed consent form and answered detailed medical, lifestyle, and nutritional questions. Disposable sterile stool sampling kits were provided to volunteers according to standard procedures with detailed instructions. Blood samples were collected using anticoagulation tubes and centrifuged at 4 °C to obtain plasma. The samples were immediately stored at −80 °C for gut microbiota analysis and metabolite detection.

### 2.2. High-throughput sequencing

Genomic DNA extraction kit (Personalbio, Shanghai, China) was used to extract genomic DNA following the manufacturer's protocol, and the 16S rRNA gene (V1‒V9 regions) was amplified using universal primers under optimized thermal cycling conditions after quality and concentration assessment.^17^ The fecal microbial community DNA fragments were subjected to single-molecule real-time (SMRT) sequencing on the third-generation sequencing platform (the PacBio Sequel). Convert software CCS v4.0.0 (Highly Accurate Single-Molecule Consensus Reads) was employed for data conversion. The raw sequencing data were processed through cutadapt (v2.3) to remove the primer fragments and the unmatched sequence. The OTU clustering procedure was performed with Vsearch (v2.13.4_linux_x86_64). Chimeras were filtered from the quality-controlled sequence set to obtain high-quality sequences. These sequences were then clustered at 97% similarity using the cluster size module to generate an OTU table. Taxonomic annotation was conducted using QIIME2 (2019.4) and the nt database. Species composition analysis was performed at the phylum, class, order, family, genus, and species levels. The subsequent alpha diversity was characterized by Simpson, Chao1, and Shannon indices. Principal coordinate analysis (PCoA) was used to reduce dimensionality for beta diversity assessment. Linear discriminant analysis effect size (LEfSe) was performed to identify differentially abundant taxa across various taxonomic levels.

### 2.3. Targeted metabolomic analysis

Working solutions of 6 SCFAs (acetic acid, propionic acid, isobutyric acid, butyric acid, isovaleric acid, and valeric acid) and caproic acid (Sigma-Aldrich, Shanghai, China) were prepared using water and ether, respectively. The internal standard (IS) solution (4-methylvaleric acid) was prepared in ether at concentrations of 375 or 75 μg/mL. The calibration curve points, covering a concentration range from 0.02 to 500 μg/mL, were generated by a mixture containing 200 μL of SCFAs working solution series, 20 μL of caproic acid working solution series, 20 μL of IS solution, 100 μL of 15% phosphoric acid, and 260 μL of ether. Fecal samples were homogenized and then centrifuged at 4 °C for 10 min at 12,000 rpm. The supernatant or diluted plasma samples were extracted with 15% phosphoric acid, an IS solution, and ether. Before GC‒MS analysis, the samples were centrifuged at 4 °C for 10 min at 12,000 rpm after vortexing. The GC analysis was performed on the Trace 1300 gas chromatograph system (Thermo Fisher Scientific, USA). Mass spectrometric detection of metabolites was carried out on ISQ 7000 (Thermo Fisher Scientific, U.S.A) using electron impact ionization mode. Convert software (Proteowizard, v3.0.8789) was applied in raw data conversion for downstream qualitative and quantitative analysis of the metabolites.

### 2.4. *In vitro* evaluation of probiotic characteristics

*C. butyricum* used in our research was deposited in the Chinese Microbial Strain Collection Center (CGMCC No. 31386) and named *C. butyricum*-NCU-027. The activated *C. butyricum* was inoculated into RCM at 2% (v/v) and incubated in an anaerobic chamber at 37 °C for 48 h. The absorbance was measured at 600 nm at 2-h intervals starting from 0 h. After that, the growth curve was plotted with time as the horizontal coordinate and OD600 value as the vertical coordinate. Afterwards, the growth curve was plotted with time as the horizontal coordinate and OD600 value as the vertical coordinate. For the cell adhesion experiments, HT-29 cells were cultured in DMEM/F-12 medium containing 10% FBS, and when grown to 95% density, they were transferred to 6-well plates containing coverslips at a density of 1 × 10^6^cells/well overnight. 1 × 10^7^ CFU of *C. butyricum* was added to the 6-well plate and co-incubated with HT29 cells at 37 °C for 2 h. The supernatant was aspirated, rinsed with PBS, and stained with formaldehyde, and visualized under a light microscope. For the determination of tolerance to bile salts, bovine bile salts were added to the medium so that the concentrations were 0, 0.1%, 0.2%, and 0.3% (mass fraction), and the number of viable bacteria was determined by spot-plate counting when the culture reached the plateau stage. For the acid resistance assay, 1 mL of bacterial culture medium was removed at the plateau stage, centrifuged to remove the medium and washed once with PBS, and then resuspended with sterile phosphate buffer adjusted to different pH values (2.0, 3.0, 4.0, 5.0, and 7.0) in advance, incubated at 37 °C for 4 h, and then counted by plate counting. The susceptibility of *C. butyricum* to several drugs was determined using the Kirby-Bauer (K-B) (paper slide method). These included tetracycline (TET), cotrimoxazole (SXT), erythromycin (E), gentamicin (GEN), lincomycin (MY), chloramphenicol (C), ampicillin (AMP), penicillin (PEN), ciprofloxacin (CIP), and ceftriaxone (CTR). After coating the bacteria on RCM agar plates, drug-sensitive paper sheets were attached, and the diameter of the circle of inhibition was recorded after 24 h of incubation. Before performing the inhibition experiments, several pathogenic bacteria were incubated with LB medium for 24 h and then coated with LB agar medium, and then Oxford cups were placed, to which 250 μL of culture supernatant of *C. butyricum* was added, and RCM medium was used for the control group, and the size of the circle of inhibition was measured after 6–8 h.

### 2.5. Cell culture

The mouse myeloma cell line 5TGM1 (RRID: CVCL_VI66) expressing luciferase (5TGM1-Luc) was obtained from Dr. Zhen Cai (the Institute of Hematology, Zhejiang University) and used for animal model construction and *in vitro* experiments. The human myeloma cell line RPMI 8226 (RRID: CVCL_0014) was procured from the American Type Culture Collection (ATCC). RPMI 8226-Luc was purchased from Shanghai Model Organisms Center, Inc. (Cat. No. NM-GS02-1) and used for *in vitro* experiments. These cell lines have been confirmed to be free from contamination. The cells were cultured in RPMI 1640 medium containing 10% fetal bovine serum and 1% penicillin/streptomycin in a humidified atmosphere with 5% CO_2_ at 37 °C, and the culture medium was changed every 2‒3 d.

### 2.6. Animal and experimental design

Healthy 6‒8-week-old female and male C57BL/KaLwRij mice were purchased from GemPharmatech Co., Ltd. The mice were maintained under SPF conditions in a 12/12-h light‒dark cycle. They had free access to water and standard food and were acclimatized and fed for 1 week before the experiment. The 5TGM1 mouse model was constructed by injecting 5TGM1-luc cells (2 × 10^6^ cells in 200 μL of PBS) into C57BL/KaLwRij mice through the tail vein on day 0 of the experiment, and control mice were injected with the same volume of PBS. In all the groups, the number of female and male mice was equal, which was used to eliminate the potential influence of gender. The systemic tumor load of the mice was assessed by the Live Imaging System during the experiment. In detail, 5TGM1 mice were injected intraperitoneally with PBS-configured D-luciferin potassium at a dose of 150 mg/kg, and a bioluminescence assay was performed 5 min later. The mice were euthanized immediately if symptoms such as sudden weight loss, loss of appetite, hunching, or lameness appeared.

To explore the impact of the gut microbiota on MM, we conducted fecal microbiota transplantation (FMT) experiments. In this part, we first collected fresh feces in 8 donor mice on days 28–35 after the injection of 5TGM1-Luc or PBS (*n* = 4). The feces were thoroughly mixed with sterile PBS, filtered through a 70 μm filter, centrifuged, then suspended in 30% glycerol and stored in a −80 °C environment for FMT. Recipient mice were randomly divided into 3 groups (*n* = 6): (1) M group: mice received intragastric administration of normal saline once daily for 1 week, and 5TGM1-Luc cells were injected on day 0. Subsequently, the mice received gelatin normal saline gavage once every 2 d for 5 weeks; (2) FMT-HC group: mice were gavaged orally with an antibiotic cocktail consisting of metronidazole (200 mg/kg), vancomycin (100 mg/kg), neomycin sulfate (200 mg/kg), and ampicillin (200 mg/kg) once daily for 1 week. After the original intestinal bacteria were eliminated, the mice were injected with 5TGM1-Luc cells on day 0 and received fecal bacteria from C57BL/KaLwRij mice for a total of 5 weeks by gavage every 2 d; (3) FMT-MM group: mice were given fecal bacteria from 5TGM1 mice, and other intervention processes were consistent with those of the FMT-HC group. At the end of the experiment, additional assays were performed after assessing the tumor load of the mice by *in vivo* imaging.

In the experiments exploring the effects of *C. butyricum* and butyrate on the progression of MM, the mice were randomly divided into 5 groups (*n* = 6): (1) C group: the control group was composed of C57BL/KaLwRij mice; (2) M group: 5TGM1 mice; (3) B group: to enhance clinical relevance, the treatment with 0.6 mg/kg bortezomib (BTZ) was given intraperitoneally once every 3 d from week 4 onwards; (4) CB group: *C. butyricum* was incubated with a standard reinforced medium (RCM) for Clostridia culture based on anaerobic incubation at 37 °C for 24 h, and the mice were gavaged with 200 µL of bacterial culture every 2 d; BTZ was given intraperitoneally at the same time over the last 2 weeks. (5) But group: Sodium butyrate was administered by gavage at a dose of 15 mg/kg every 2 d; the use of BTZ is the same as that of B and CB groups. To eliminate the influence of the operation, the mice in the C, M, and B groups were given the same dose of PBS or saline by tail vein injection, gavage, and intraperitoneal injection as controls. The *Escherichia coli* strain (K12 MG1655, ATCC 700926) used in the EC group was obtained from Ningbo Mingzhou Bio Co., Ltd. and cultured in BHI medium aerobically at 37 °C for 24 h preceding intragastric administration.

Healthy 6–8-week-old female M-NSG mice (NOD.Cg-*Prkdc* ^scid^ *Il2rg* ^em1Smoc^, Cat. NO. NM-NSG-001) were purchased from Shanghai Model Organisms Center, Inc. The immunodeficient MM mice model was established by intravenous injection of RPMI 8226-luc cells (5 × 10^6^ cells in 200 μL of PBS) via the tail vein on day 0, while control mice received an equal volume of PBS. The housing conditions, as well as the bacterial gavage and BTZ treatment strategies, were identical to those used for the C57BL/KaLwRij mice in this study.

### 2.7. Sample collection and cell preparations

Bone marrow single-cell suspension preparation: Remove the two epiphyses of the mouse femur and rinse the bone marrow cavity with 300 μl of precooled PBS. The rinsate was centrifuged at 500 × g for 5 min at 4 °C, and the supernatant was stored at −80 °C for ELISA or other assays. The cells were then resuspended with pre-cooled PBS supplemented with 5% fetal bovine serum and passed through a 70 μm nylon cell filter to obtain a single-cell suspension for further flow cytometry analysis. Erythrocyte lysis was performed before the experiment.

Spleen single-cell suspension preparation: a small portion of spleen tissue was cut and washed twice with PBS, placed in a Petri dish, gently grinded after adding PBS, the resulting suspension was centrifuged to remove the supernatant, 1 mL of 0.2% collagenase II was added, incubated in a water bath at 37 °C for 10 min, and single-cell suspensions were obtained by passing through a 70 μm filter. Erythrocyte lysis was performed before the experiment.

Preparation of a single-cell suspension of intestinal lymphoid tissue: 6–8 Peyer's patches were taken and washed twice with PBS, and were sheared in a Petri dish with PBS. The fragments were transferred to a centrifuge tube, centrifuged, the supernatant was removed, and 10 mL of isolation solution (HBSS + 2 mM EDTA + 1 mM DTT + 10 mM HEPES) was added, and incubated at 37 °C in a water bath for 30 min, and repeated once. The tissue fragments were placed into a new centrifuge tube, 10 mL of digestion solution (PBS + 1.5 g/mL collagenase A + 100KU/L DNA I) was added, and incubated in a water bath at 37 °C for 30 min. Single cell suspensions were then obtained by passing through a 70 μm filter.

Blood was collected, allowed to stand at room temperature for one hour, and then centrifuged at 4 °C at 3,000 rpm for 15 min. The obtained serum was aliquoted and stored at −80 °C. The tibiae, along with portions of the colon and spleen, were photographed before being fixed in 4% paraformaldehyde for morphological analyses and immunohistochemistry. The remaining tissues were stored at −80 °C for molecular biology experiments.

### 2.8. Flow cytometry

The obtained single cell suspension was washed once with PBS, resuspended in cell staining buffer, and counted. And 1 × 10^6^ cells were taken for subsequent experiments. Surface molecular staining was performed by adding the appropriate fluorescent labeling antibody. After incubation, the cells were fixed and membrane-broken and subjected to IL-17A antibody, mixed well, and incubated for 30 min at room temperature and protected from light. The assay was performed on a flow cytometer (Beckman CytoFLEX), and the data were analyzed using FlowJo software. The antibodies used in the experiments included: CD4-PE (clone GK1.5, Elabscience, E-AB-F1097D), CD3-FITC (clone 17A2, Elabscience, E-AB-F1013C), CD8a-APC (clone 53-6.7, Elabscience, E-AB-F1104E), F4/80-FITC (clone CI:A3-1, Elabscience, E-AB-F0995C), CD11b-PE (clone M1/70 Elabscience, E-AB-F1081D), IL-17 APC (clone TC11-18H10.1, Elabscience, E-AB-F1199E), IL-17A PerCP-Cy5.5 (clone TC11-18H10, BD, 560666), CD19-FITC (clone 1D3, Elabscience, E-AB-F0986C), CD45R/B220-PE (clone RA3.3A 1/6.1, Elabscience, E-AB-F1112D).

### 2.9. Micro-CT analysis

A micro-CT scanner (NEMO, Pingsheng Medical Technology, NMC-200) was used to continuously scan the tibia and femur of mice at 90 KV, 0.04 mA, with a scanning accuracy of 14 μm. The original images were reconstructed using Recon software, and the proximal end of the tibia was subjected to ROI analysis using the data analysis software, Avatar, and the data were exported. The analysis parameters included: bone volume fraction (BV/TV), bone surface area to tissue volume ratio (BS/TV), bone surface area to bone volume ratio (BS/BV), trabecular thickness (Tb.Th), trabecular separation (Tb.Sp), number of trabeculae (Tb.N), trabecular connectivity density (Conn.D), degree of anisotropy (DA), fractal dimension (FD), bone mineral density (BMD), bone mineral content (BMC), tissue mineral density (TMD), and tissue mineral content (TMC).

### 2.10. TRAP staining and histomorphometry analysis of tibiae

To observe the number of osteoclasts, we decalcified and embedded the bone tissue and made 4 μm-thick paraffin sections. Then, we performed TRAP staining and observed the number of osteoclasts under the microscope. To analyze the results more precisely, we used ImageJ software for image analysis. Briefly, the isolated femoral tissues were immediately fixed in 4% paraformaldehyde for 1 week and then placed in EDTA decalcification solution for another 4 weeks. Paraffin-embedded sections were sectioned to a thickness of 4 μm and stained with TRAP. The subchondral bone near the cartilage border was selected as the definite observation area in microscopic examination and image acquisition.

### 2.11. Enzyme-linked immunosorbent assay (ELISA)

For the detection of inflammatory factors in colon tissue fluid, 50 mg of colon tissue was first washed in precooled PBS, and then, 1 mL of PBS was added to cut and grind it, centrifuged at 500 × *g* for 5 min at 4 °C, and the supernatant was collected and stored at −80 °C. Stool samples were spiked with 5 mL of PBS per 100 mg, shaken well, centrifuged at 6000 rpm for 5 min at 4 °C, and the supernatant was aspirated for the butyric acid concentration assay. The inflammatory factors TNF-α, IL-6, IL-1β (Boster, Wuhan, China), and IL-17 (FineTest, Wuhan, China) were detected using enzyme-linked immunosorbent assay (ELISA) kits according to the manufacturer's instructions. Similarly, RANKL (Boster, Wuhan, China), CCL20, the bone metabolism markers CTXI and PINP, and IgG2b secreted by tumor cells were detected using ELISA kits (FineTest, Wuhan, China).

### 2.12. Cell viability assay

5TGM1 or RPMI 8226 cells were inoculated into four 96-well plates at a density of 8 × 10^3^ cells/well, and the blank control wells contained medium only. 24 h later, sodium butyrate solution was added to a final concentration of 0, 0.20, 0.05, 0.1, 0.2, 0.4, 0.6, 0.8, 1, and 1.5 mM, respectively, and the incubation was continued for 24, 48, 72, and 96 h. The cells were then incubated with sodium butyrate for 24, 48, 72, and 96 h, respectively. During this period, fresh medium was replenished every 2 d with 10–20 μL, depending on consumption. Cell viability was assessed using Cell Counting Kit-8 at the indicated times according to the manufacturer's instructions. 10 µl of CCK-8 reagent was added to each well and incubated for 2 h at 37 °C in 5% CO_2_. Cell viability was calculated by measuring the absorbance at 450 nm using an enzyme meter.

### 2.13. Apoptosis and cell cycle assay

Apoptosis was analyzed using the Annexin V-FITC/PI Apoptosis Kit. MM cell lines were cultured in medium containing 1.5 mM sodium butyrate for 24 h and then collected thoroughly and washed with PBS. The cells were resuspended with Buffer and added with FITC stain and PI stain, set up with both blank and single staining tubes, and incubated at room temperature and protected from light for 15 min. For the cell cycle assay, the cells in each group were prefixed with anhydrous ethanol and allowed to stand overnight at 4 °C. The cells were fixed with PBS, and the cell cycle was analyzed using the PI apoptosis kit. The fixed cells were washed with PBS, mixed with staining buffer: PI staining solution: RNase A = 100:5:2 and resuspended, and incubated for 30 min at 37 °C, protected from light. The apoptosis rate and cell cycle distribution were detected by flow cytometry and analyzed by FlowJo and MODFIT software.

### 2.14. RNA-seq

To explore the potential mechanism by which butyrate exerts antitumor effects, gene expression analysis was performed on 5TGM1 cells (*n* = 4) treated with 0.5 mM sodium butyrate (M group) or PBS (C group) for 48 h, respectively. Total RNA was extracted, reverse-transcribed, and purified, followed by library preparation and quality control. Sequencing was performed based on the Illumina sequencing platform (Shanghai Weihuan Biotechnology Co., Ltd.). After obtaining the raw sequencing reads, the fastp software was utilized to remove adapters and filter low-quality data. The clean data were mapped to the reference genome sequence, and gene expression levels were quantified using Stringtie. Differentially expressed genes (DEGs) analysis was performed using DESeq2 and identified based on a significance threshold of fold change > 1 and *p*-value < 0.05. The clusterProfiler was used to perform Gene Ontology (GO) enrichment analysis and Kyoto Encyclopedia of Genes and Genomes (KEGG) pathway enrichment analysis to identify significantly enriched metabolic or signaling pathways in the two groups.

### 2.15. Western blot

Total intestinal tissue, tumor tissue, and cellular proteins were extracted with RIPA lysis buffer containing protease inhibitors and phosphatase inhibitors, and the concentrations were determined by the BCA protein quantification kit. Proteins were separated by 8%–12% sodium dodecyl sulfate polyacrylamide gel electrophoresis (SDS‒PAGE) and transferred to 0.2–0.45 μm PVDF membranes. The nonspecific binding sites were blocked by occlusion with 5% BSA for 1.5 h at room temperature, followed by incubation with the addition of primary antibody and overnight at 4 °C. After a TBST wash, the membrane was incubated with an enzyme-labeled secondary antibody for 2 h at room temperature. Finally, the protein was imaged in a fully automated gel imaging analysis system (Tanon 5200) using an ultrasensitive chemiluminescent (Super ECL) substrate working solution. Proteins were quantified and analyzed using ImageJ.

### 2.16. Real-time fluorescence quantitative PCR analysis

Total fecal mRNA was extracted using the QIAamp Fecal DNA Rapid Purification Mini Kit according to the manufacturer's instructions, and for total RNA in tissues, the TRIzol reagent was used. Subsequently, the concentration of RNA was determined using a NanoDrop 2000, and quantitative PCR was performed. The cDNA was then synthesized by reverse transcription using the PrimeScript™ RT Kit (containing the gDNA removal reagent), and TB Green® premixed with Ex Taq™ II was used for real-time fluorescence quantification, and the mRNA content of the target primers was detected on the ViiA 7 Real-Time PCR System. Finally, the relative mRNA expression level of each target gene was determined using the 2−ΔΔCt method.

### 2.17. Statistical methods

The number of samples used in each study is represented by dots in the graphs, and the data are presented as mean ± SEM. Regarding the clinical data, normally distributed continuous variables are expressed as mean ± standard deviation (SD), non-normally distributed variables as median (interquartile spacing), and categorical variables as numbers and percentages. Non-normally distributed data were compared with the independent samples chi-square (*χ*²) test or Mann‒Whitney *U* test, and data conforming to a normal distribution were compared with the unpaired *t* test for differences between groups. For the animal experiment part, statistical significance was analyzed by one-way analysis of variance (ANOVA): Dunnett's multiple comparison test (both using groups compared with group M). All tests were judged to be statistically significant with a two-sided *p* < 0.05 for differences. Statistical analysis was performed using GraphPad Prism (version 9) and SPSS software (version 26).

## 3. Results

### 3.1. The gut microbiota alterations in MM are characterized by depletion of butyrate producers

**Figure 1.**
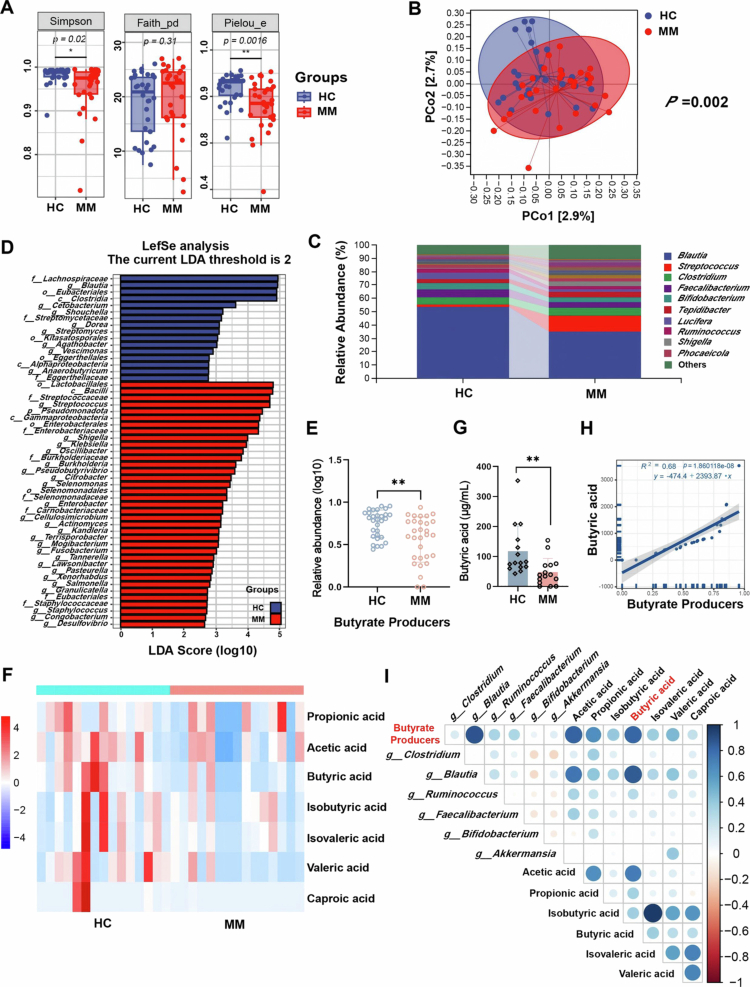
The gut microbiota alterations in MM patients are characterized by the depletion of butyrate-producing bacteria. A) Alpha diversity of the microbial community. B) Beta diversity of the microbial community. C) Genus-level classification histogram. D) Histogram of the linear discriminant analysis effect size showing the relative abundance of bacteria. E) Butyrate-producing bacterial abundance (mean ± SEM, *n* = 30, and **p < 0.01 by Mann‒Whitney *U* test). F) Heat map of fecal short-chain fatty acids (SCFAs) targeted metabolomics. G) Butyric acid level in feces (mean ± SEM, *n* = 15, and **p < 0.01 by Mann‒Whitney *U* test). H) Linear correlation between butyrate-producing bacteria abundance and butyric acid levels. I) Correlation heat map of fecal SCFAs and common butyrate-producing bacteria. MM: multiple myeloma; HC: healthy controls.

To investigate the potential impact of the gut microbiota on MM, full-length 16S rRNA sequencing (regions V1–V9) was performed on fecal samples from 30 newly diagnosed MM patients and 30 healthy controls (HC). Compared with HC, MM patients exhibited markedly reduced α-diversity, as indicated by Simpson diversity indices (*p* = 0.02) and Pielou's evenness homogeneity (*p* = 0.0016) (Figures 1A and S1A). β-Diversity analysis confirmed the differences in the gut microbiota composition between the MM and HC groups (*p* = 0.002) (Figure 1B). Genus-level taxonomic profiling further revealed that, compared with HC, MM patients exhibited decreased relative abundances of *Blautia*, *Faecalibacterium*, and *Bifidobacterium*, and elevated abundances of opportunistic pathogenic bacteria such as *Streptococcus* and *Shigella* (Figures 1C and S1B). Linear discriminant analysis (LDA) effect size (LEfSe) showed that Clostridia and their members, such as *Blautia*, *Dorea*, *Agathobacter*, and *Anaerobutyricum*, were enriched in the HC group (Figures 1D and S1C). This pattern suggests that MM progression may correlate with the depletion of specific microbiota rather than the expansion of pathogenic species because HC-enriched taxa were associated with SCFAs biosynthesis, particularly the production of butyrate. Therefore, we analyzed several common bacteria that can produce SCFAs. Although there was no significant difference in the relative abundance of *Clostridium*, *Faecalibacterium*, *Ruminococcus*, *Lactococcus*, and *Bifidobacterium* (Figure S1D), it was worth noting that the overall abundance of butyrate producers in MM patients was significantly reduced (*p* = 0.0035) (Figure 1E). Furthermore, fecal metabolomics targeting SCFAs indicated a significant decrease of butyrate levels in MM patients (*p* = 0.0017) (Figures 1F,G, and S1E). The correlation analysis further illustrated the significant positive correlation between butyrate levels and the total abundance of butyrate producers (Figures 1H,I and S1F,G). These findings established the structural and functional gut microbiota dysbiosis in MM patients, highlighting the potential therapeutic implications of butyrate in MM pathogenesis.

### 3.2. Healthy fecal microbiota transplantation inhibits MM progression

**Figure 2.**
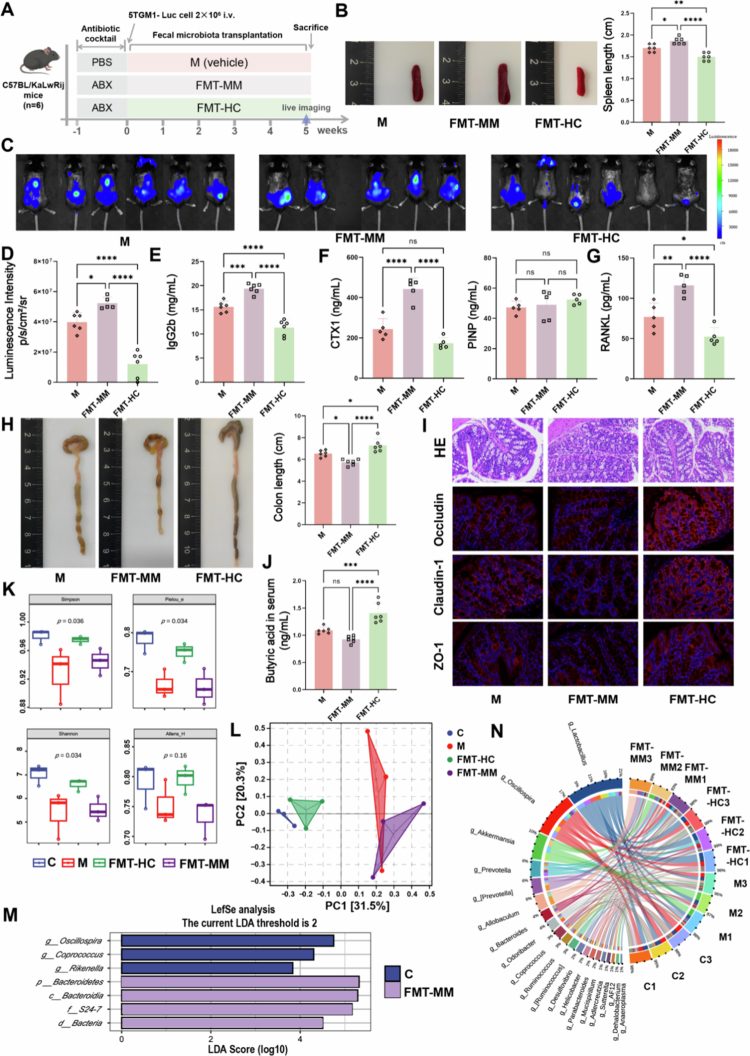
Fecal microbiota transplantation critically modulates MM progression in 5TGM1 mice**.** A) Experimental design schematic diagram. B) Spleen length and representative images (mean ± SEM, *n* = 6, and **p < 0.01 by one-way ANOVA). C) Live imaging of the mice at week 5. D) Quantification of luminescence intensity in live imaging (mean ± SEM, *n* = 5–6, *p < 0.05, and ****p < 0.0001 by one-way ANOVA). E) Concentration of serum IgG2b (mean ± SEM, *n* = 6, ***p < 0.001, and ****p < 0.0001 by one-way ANOVA). F) Concentration of bone metabolism markers in the serum (mean ± SEM, *n* = 5, *p < 0.05, **p < 0.01, and ****p < 0.0001 by one-way ANOVA). G) Concentration of RANKL in the bone marrow (mean ± SEM, *n* = 5, *p < 0.05, **p < 0.01, and ****p < 0.0001 by one-way ANOVA). H) Colon length and representative images (mean ± SEM, *n* = 6, and *p < 0.05 by one-way ANOVA). I) H&E staining images of intestinal tissues and immunofluorescence staining for intestinal tight junction proteins, scale bar: 100 μm. J) Serum butyric acid levels (mean ± SEM, *n* = 6, and ***p < 0.001 by one-way ANOVA). K) Alpha diversity. L) PCoA analysis of beta diversity. M) Histogram of the linear discriminant analysis effect size showing the relative abundance of bacteria. N) Species composition and chordal diagram. *n* = 3 (K)–(N). ABX: antibiotic cocktail; FMT: fecal microbiota transplantation. CTX1: cross-linked C-telopeptide of type I collagen; PINP: N-terminal propeptide of the procollagen type I; RANKL: nuclear factor κB receptor activator ligand; ZO-1: zonula occludens-1.

To explore whether the gut microbiota can be an intervention target for MM, we conducted fecal microbiota transplantation (FMT) experiments in a MM mouse model established by intravenous injection of 5TGM1 cells into C57BL/KaLwRij mice. After tumor inoculation, the mice were orally gavaged with gelatin normal saline (M) or feces from healthy C57BL/KaLwRij (FMT-HC) or MM mice (FMT-MM) (Figure 2A). We found that the tumor burden of the FMT-HC group was significantly lower than that in M and FMT-MM groups, including the systemic luminescence intensity (*p* < 0.0001) (Figure 2C,D) and serum IgG2b concentration (*p* < 0.0001) (Figure 2E). Splenic hyperplasia was also alleviated in FMT-HC group (Figure 2B). In addition, bone disease is an important manifestation and progression marker of MM. Therefore, we evaluated the serum levels of cross-linked C-telopeptide of type I collagen (CTXI) and the N-terminal propeptide of the procollagen type I (PINP), which reflect osteoclast (OC) and osteoblast (OB) activity, respectively. The ELISA results showed that CTXI was significantly increased in FMT-MM group, while there was no meaningful difference in PINP (Figure 2F). Consistently, the content of nuclear factor κB receptor activator ligand (RANKL), the key regulatory factor for OC differentiation in the bone marrow, was decreased in the FMT-HC group (*p* = 0.0297) (Figure 2G), suggesting that the gut microbiota may have a more crucial impact on OC rather than OB.

As the direct target organ of FMT, the intestine was also evaluated. FMT-MM group exhibited notably shortened colon length (*p* = 0.0104) (Figure 2H) and reduced expression of tight junction proteins, the key structural components responsible for maintaining intestinal barrier integrity (Figures 2I and S2E). Conversely, FMT from healthy mice effectively reversed these intestinal pathologies associated with MM and increased the concentration of butyrate in the serum and feces (*p* = 0.0007) (Figures 2J and S2D). Finally, we analyzed alterations in the gut microbiota to verify the effectiveness of FMT. The α-diversity of group M and FMT-MM was significantly lower than that of the healthy C57BL/KaLwRij mice (C) and the FMT-HC group (Figures 2K and S2F). Venn diagram and β-diversity analysis revealed distinct microbiota characteristics, confirming successful colonization of the microbiota derived from healthy or MM mice in recipient mice (Figures 2L and S2G). LEfSe analysis showed that relative to those in the other groups, *Oscillospira*, *Coprococcus*, and *Rikenella*, which were important sources of SCFAs, were significantly enriched in the feces of group C (Figure 2M). The chord diagram and mulberry diagram showed the specific differences in genus composition across samples (Figures 2N and S2H). In conclusion, these results indicate that the gut microbiota is involved in the progression of MM and healthy fecal bacteria have potential therapeutic effects.

### 3.3. *C. butyricum* and its derived butyrate enhance the antimyeloma efficacy of bortezomib

**Figure 3.**
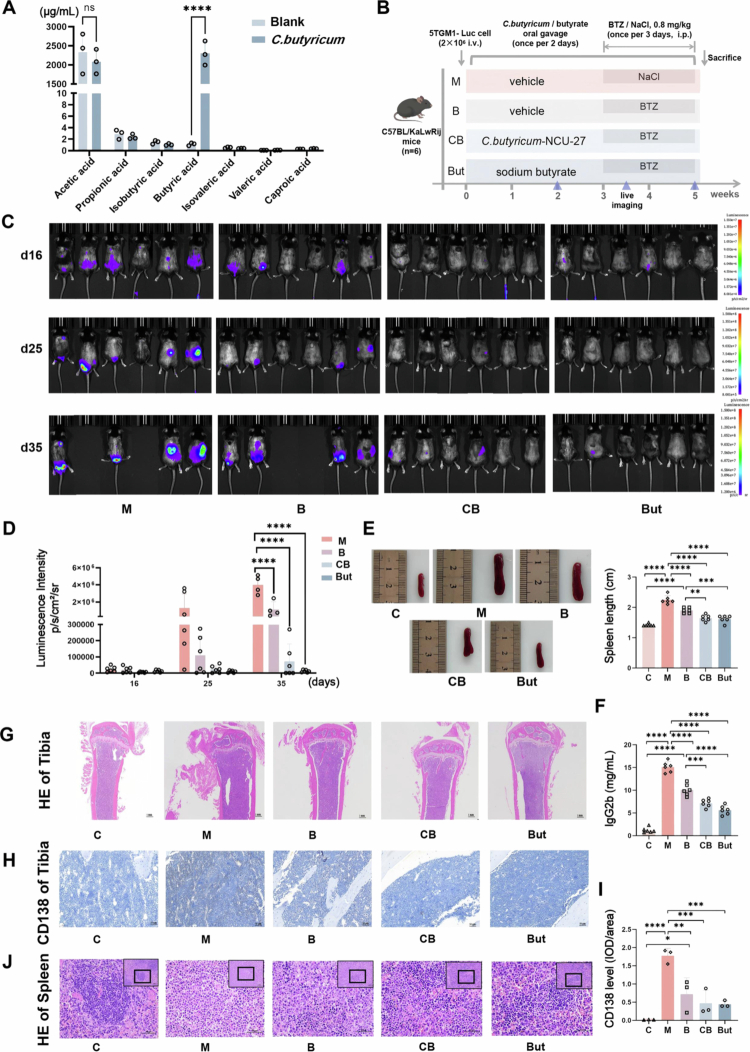
*C. butyricum* and its derived butyrate enhance the antimyeloma efficacy of bortezomib. A) Targeted metabolomics analysis of short-chain fatty acids in *C. butyricum* culture supernatants (mean ± SEM, *n* = 3, and ****p < 0.0001 by Mann‒Whitney *U* test). B) Experimental design schematic diagram. C) Live imaging of the mice. D) Quantification of luminescence intensity in live imaging (mean ± SEM, *n* = 4–6, and ****p < 0.0001 by one-way ANOVA). E) Spleen length and representative images (mean ± SEM, *n* = 6, and ****p < 0.0001 by one-way ANOVA). F) Concentration of serum IgG2b (mean ± SEM, *n* = 6, and ****p < 0.0001 by one-way ANOVA). G) Representative images of H&E staining of the tibia, scale bar: 1 mm. H) Immunohistochemical staining for CD138 in the bone marrow, scale bar: 50 μm. I) Quantification of CD138 immunohistochemical staining (mean ± SEM, *n* = 3, **p < 0.01, ***p < 0.001, and ****p < 0.0001 by one-way ANOVA). J) Representative H&E staining of the spleen showing the histoarchitectural damage, scale bar: 50 μm.

To identify the key probiotic species with therapeutic potential, we collected the feces of healthy volunteers for bacterial culture and screened out a representative butyrate producer, *Clostridium butyricum* (*C. butyricum*). Metabolomics analysis of SCFAs verified the high concentration of butyric acid in its culture supernatant (*p *< 0.0001) (Figure 3A). Notably, by investigating and comparing the butyric acid production capacity of known common butyrate producers, we found that *C. butyricum* exhibited a remarkable advantage in butyrate production, exceeding that of most documented butyrate producers by more than 50% (Figure S3B). We further recorded the growth characteristics (Figure S3A) and evaluated the probiotic properties of *C. butyricum in vitro.* Antibiotic susceptibility testing showed that *C. butyricum* was sensitive to common antibiotics (Figure S3C). Meanwhile, *C. butyricum* was tolerant to bile salts and acidic environments (Figure S3D,E), and could adhere to intestinal cells (Figure S3F), suggesting its advantages as an oral probiotic. These results provided a prerequisite for its function *in vivo*.

First, we evaluated the effects of *C. butyricum* on MM in combination with bortezomib (BTZ) therapy, given the relatively rapid progression of MM in mice and the fact that combination treatment better reflects clinical therapeutic principles. The 5TGM1 mice were treated with BTZ and gavaged with either gelatin normal saline (B group), *Escherichia coli* (*E. coli*) (EC group) or *C. butyricum* (CB group) (Figure S4A). Living imaging (Figure S4B,D) and the detection of serum IgG2b (Figure S4C) showed that *C. butyricum* significantly inhibited MM progression compared to *E. coli*. In addition, HE staining of the spleen revealed less structural damage in the CB group than in group B and EC (Figure S4G). Meanwhile, CD138 immunohistochemical staining of the femur indicated that the accumulation of MM cells in the bone marrow was significantly reduced in the CB group (Figure S4E,F). Then, we further explored the *in vivo* effects of butyrate (Figure 3B). Compared with MM mice receiving gelatin saline (Group M) and BTZ treatment alone (Group B), supplementation of *C. butyricum* (Group CB) or sodium butyrate (Group But) in combination with BTZ significantly reduced the tumor burden (*p* < 0.0001), including the luminescence intensity (Figure 3C,D), splenic volume (Figure 3E), and serum IgG2b concentration (Figure 3F). CD138 immunohistochemical staining of the tibia and spleen HE staining showed that, relative to the M and B groups, the CB and But groups exhibited a significant reduction in MM cell accumulation within the bone marrow (*p *< 0.001) (Figure 3G–I) and spleen (Figure 3J). These evidences demonstrate the great potential of combining *C. butyricum* or butyrate with BTZ to improve MM.

### 3.4. *C. butyricum* and butyrate alleviate gut damage caused by MM and bortezomib

**Figure 4.**
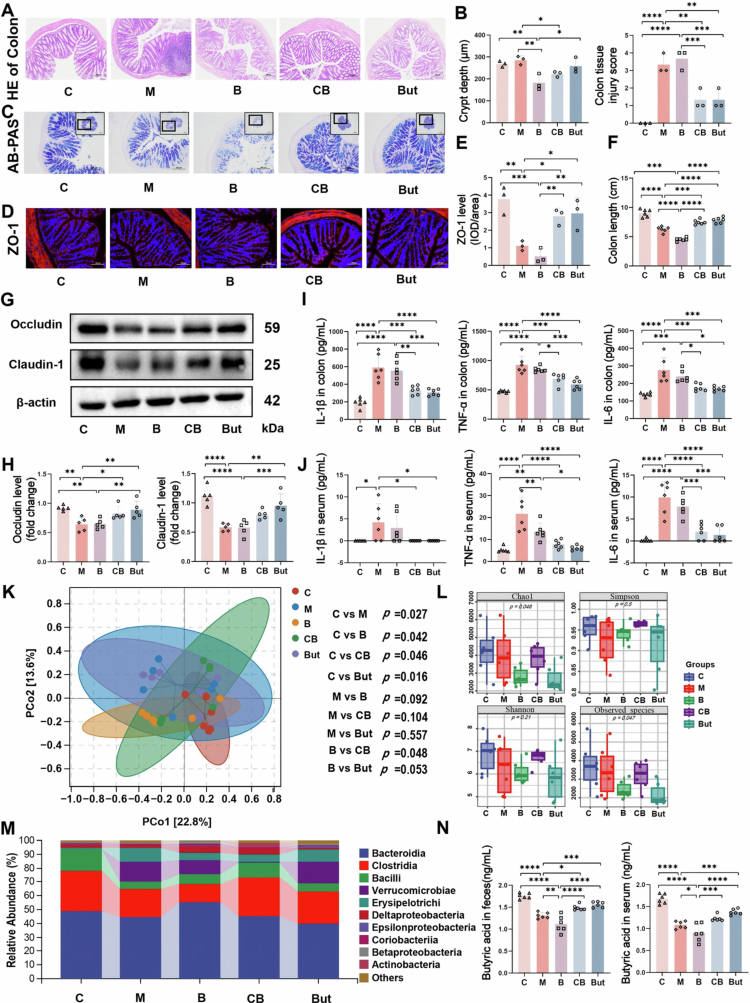
*C. butyricum* and butyrate alleviate the gut damage caused by MM and bortezomib. A) Representative images of H&E staining of the colon, scale bar: 200 μm. B) Crypt depth and tissue injury score of the colon (mean ± SEM, *n* = 3, *p < 0.05, **p < 0.01, and ****p < 0.0001 by one-way ANOVA). C) AB-PAS staining of colon, Scale bar: 200 μm. D) Representative images of immunofluorescence staining for ZO-1, Scale bar: 100 μm. E) Quantification of ZO-1 immunofluorescence staining (mean ± SEM, *n* = 3, *p < 0.05, and **p < 0.01 by one-way ANOVA). F) Colon length (mean ± SEM, *n* = 6, and ****p < 0.0001 by one-way ANOVA). G) Western blot analysis of occludin and claudin-1 expression in intestinal tissues. H) Quantitative results of occludin and claudin-1 expression (mean ± SEM, *n* = 5, *p < 0.05, **p < 0.01, and ****p < 0.0001 by one-way ANOVA). I) Inflammatory factor levels in the colon (mean ± SEM, *n* = 6, and ****p < 0.0001 by one-way ANOVA). J) Serum inflammatory factor levels (mean ± SEM, *n* = 6, *p < 0.05, **p < 0.01, ****p < 0.0001 by one-way ANOVA). K) Beta diversity. L) Alpha diversity. M) Class-level classification histogram. N) Butyric acid content in feces and serum. *p* values were determined by the Mann‒Whitney *U* test (mean ± SEM, *n* = 6, *p < 0.05, **p < 0.01, ***p < 0.001, and ****p < 0.0001 by one-way ANOVA). AB-PAS: Alcian blue-periodic acid Schiff; IL-1β: interleukin-1β; TNF-α: tumor necrosis factor-α.

The evaluation of the intestinal tract showed that compared with group C, the colon length of mice in group M was shorter (Figures 4F and S5A) and the injury score was increased (Figure 4A,B), which reflected the effects of MM as a systemic disease on the intestinal tract. In addition, similar to the gastrointestinal adverse effects such as diarrhea, constipation, nausea, and vomiting induced by BTZ in the clinic, we observed that intestinal damage could not be recovered, even though BTZ treatment significantly reduced the systemic tumor burden in mice. While, supplementation with *C. butyricum* or butyrate increased colon length and intestinal crypt depth (Figures 4A,B,F and S5A), restored the number of goblet cells (Figure 4C) and the expression of ZO-1 (Figure 4D,E), occludin and claudin-1 (*p* < 0.05) (Figure 4G,H), thereby improving intestinal barrier function. Moreover, ELISA revealed that the contents of the inflammatory factors IL-6, TNF-α, and IL-1β in the colon tissues and serum of the mice in the CB and But groups were significantly lower than those in Groups B and M (Figure 4I,J), suggesting that *C. butyricum* and butyrate not only ameliorated intestinal inflammation caused by tumor and the adverse effects of BTZ but also further improved systemic inflammation and thereby affect MM progression.

Furthermore, analysis of the gut microbiota showed an increased *α*-diversity in the CB group compared to the M and B groups, which was consistent with a distinct overall microbial composition (Figure 4K,L). Specifically, *C. butyricum* and butyrate increased the abundance of *Lactobacillaceae*, *Ruminococcaceae*, and *Lachnospiraceae* of Clostridia (Figures 4M and S5E). Although butyrate did not modulate the α-diversity as effectively as live bacteria, a functional complex, it still enhances the enrichment of beneficial genera such as *Akkermansia* (Figure S5D). The relative abundance of beneficial genera such as *Lactobacillus*, *Lactococcus*, *Bifidobacterium*, and *Roseburia* exhibited an increasing trend (Figure S5F), and prolonged intervention may lead to pronounced effects. Meanwhile, quantitative PCR analysis showed that *C. butyricum* was able to colonize the intestines (Figure S5B). We further verified the significant increase of butyric acid in the feces and serum of group CB and But (*p* < 0.05) (Figure 4N). In conclusion, *C. butyricum* can produce butyrate to alleviate intestinal damage caused by MM and the adverse reactions of BTZ.

### 3.5. Butyrate remodels the bone marrow microenvironment in 5TGM1 mice

**Figure 5.**
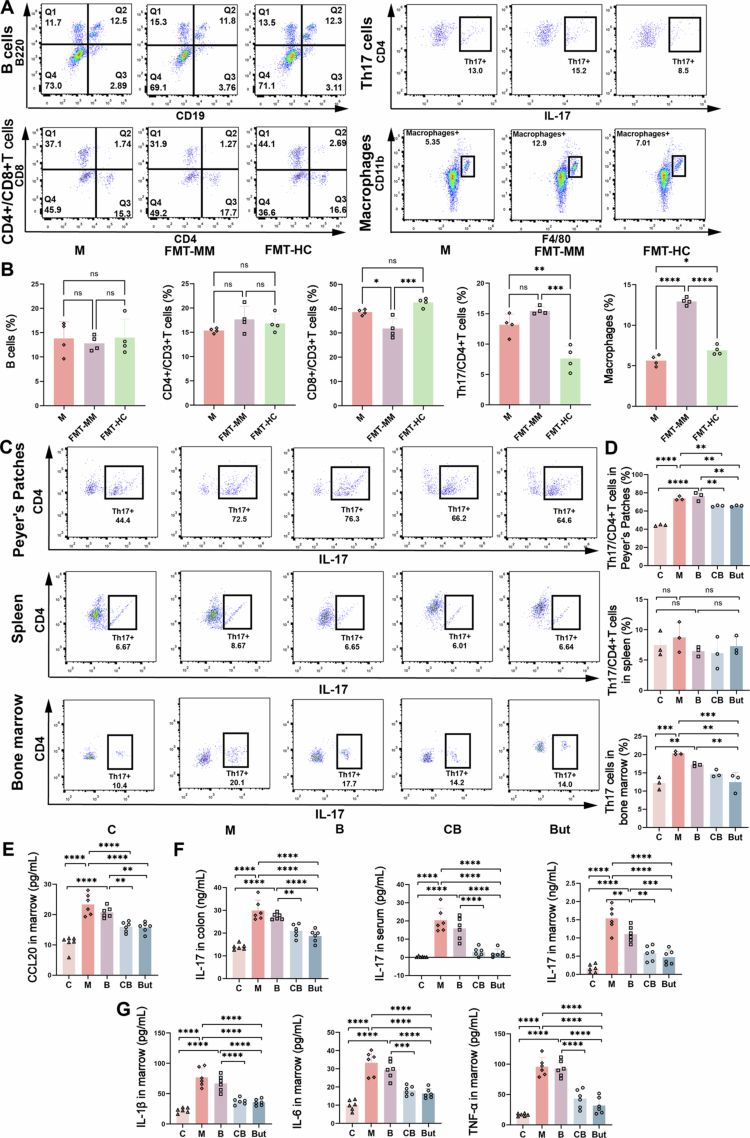
Butyrate inhibits the trafficking of Th17 cells from the intestine to the bone marrow and alleviates inflammation. A) Flow cytometric analysis of immune cells in the bone marrow following fecal microbiota transplantation. B) The percentage of immune cells in the bone marrow (mean ± SEM, *n* = 4, *p < 0.05, **p < 0.01, and ****p < 0.0001 by one-way ANOVA). C) Flow cytometric analysis of Th17 cells in Peyer's patches, spleen, and bone marrow. D) The percentage of Th17 cells in Peyer's patches, spleen, and bone marrow (mean ± SEM, *n* = 3, **p < 0.01, and ****p < 0.0001 by one-way ANOVA). E) The concentration of CCL20 in the bone marrow (mean ± SEM, *n* = 6, **p < 0.01, and ****p < 0.0001 by one-way ANOVA). F) The concentration of IL-17 in colon, serum, and bone marrow (mean ± SEM, *n* = 6, **p < 0.01, and ****p < 0.0001 by one-way ANOVA). G) The concentration of inflammatory factors in the bone marrow (mean ± SEM, *n* = 6, **p < 0.01, and ****p < 0.0001 by one-way ANOVA).

Restoration of antitumor immunity is one of the most important targets for MM treatment, while the gut microbiota can influence immune homeostasis in various ways, such as by modulating the intestinal barrier and systemic inflammation and producing active metabolites. To investigate whether immune regulation contributes to the effects of *C. butyricum*, we examined several immune cell groups in the bone marrow of MM mice following FMT. Flow cytometry analysis revealed that the proportion of macrophages was significantly elevated in the FMT-MM group (*p* < 0.0001), whereas transplantation of healthy fecal bacteria led mainly to a decrease in the proportion of Th17 cells (*p* = 0.0011) (Figure 5A,B), with the changes in Th17 cells showing an opposite trend in FMT-MM and FMT-HC groups. This result reflected the positive response of Th17 cells to changes in the gut microbiota and their potential impact on the function of probiotics. Given the unique ability to migrate from the intestine to the inflammatory site, Th17 cells may serve as pivotal mediators in the gut‒bone axis. We further analyzed the proportions of Th17 cells in Peyer's patches (lymphoid tissue in the intestinal mucosa), spleen, and bone marrow in single bacterial intervention experiment. The results showed that the proportions of Th17 cells in Peyer's patches of groups M and B were increased compared with group C (*p* < 0.0001), whereas their differentiation from CD4^+^ T cells was significantly reduced in group CB and But (*p* = 0.0067) (Figure 5C,D). More importantly, the difference in Th17 cells in the bone marrow between the groups was consistent with that observed in Peyer's patches (Figure 5C,D). In addition, compared to group M and B, CCL20 (a key chemokine that mediates the migration of peripheral Th17 cells to the bone marrow) levels in the bone marrow were also downregulated in group CB and But (Figure 5E). The lack of significant variation in splenic Th17 cell numbers might be attributed to their homing to the bone marrow. Th17 cells are closely associated with bone marrow inflammation and contribute to the proliferation of MM cells and to bone destruction. ELISA analysis showed that the levels of IL-17 in intestinal tissues, serum, and bone marrow, as well as the levels of IL-1β, IL-6, and TNF-α in the bone marrow, were significantly reduced in the CB and But groups (*p* < 0.0001) (Figure 5F,G). In conclusion, these results suggest that *C. butyricum* and butyrate maintain BMM homeostasis and thereby suppress MM progression via the inhibition of Th17 cells.

### 3.6. Butyrate-mediated BMM remodeling alleviates myeloma bone disease by suppressing osteoclasts

**Figure 6.**
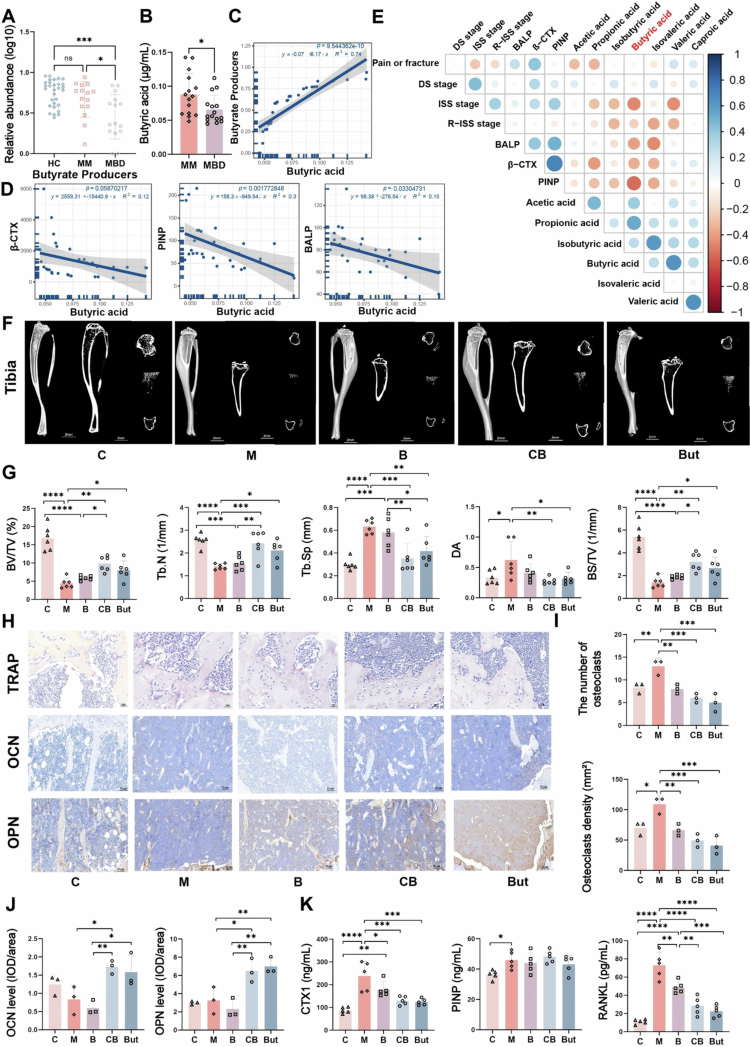
Myeloma bone disease correlates with reduced butyrate levels and can be mitigated through treatment with *C. butyricum* or butyrate. A) The relative abundance of butyrate-producing bacteria in multiple myeloma patients with or without bone disease (mean ± SEM, *n* = 15, and *p < 0.05 by Mann‒Whitney *U* test). B) Serum butyric acid concentrations (mean ± SEM, *n* = 15, and *p < 0.05 by Mann‒Whitney *U* test). C) Linear correlation between butyrate-producing bacteria abundance and butyric acid levels. D) Linear correlation between butyric acid levels and bone metabolism markers in the serum. E) Correlation heat map of clinical indicators and short-chain fatty acids. F) Representative three-dimensional reconstruction images of the tibia, scale bar: 2 mm. G) Quantitative results of bone parameters, including BV/TV, Tb. *N*, Tb.Sp, DA, and BS/TV (mean ± SEM, *n* = 6, *p < 0.05, **p < 0.01, ***p < 0.001, and ****p < 0.0001 by one-way ANOVA). H) Representative images of tartrate-resistant acid phosphatase staining and OCN/OPN immunohistochemistry of the tibia. Scale bar: 20–50 μm. I) and J) Quantitative results of osteoclasts, OCN, and OPN (mean ± SEM, *n* = 3, *p < 0.05, **p < 0.01, and ***p < 0.001 by one-way ANOVA). K) The concentration of bone metabolism markers in the serum and the RANKL level in the bone marrow (mean ± SEM, *n* = 5, *p < 0.05, **p < 0.01, ***p < 0.001, and ****p < 0.0001 by one-way ANOVA). β-CTX: β-C-terminal telopeptide of type I collagen; CTX1: cross-linked C-telopeptide of type I collagen; PINP: N-terminal propeptide of the procollagen type I; BALP: bone alkaline phosphatase; DS: Durie–Salmon staging system; ISS: international staging system; R-ISS: revised international staging system; OCN: osteocalcin; OPN: osteopontin; BV/TV: bone volume fraction; Tb.N: number of trabecular bone; Tb.Sp: bone trabecular separation; DA: anisotropic degree; BS/TV: bone surface area and tissue volume ratio.

Given the regulatory ability of *C. butyricum* on the bone marrow immune environment and inflammatory state, we speculate that it may consequently alleviate MBD. First, we analyzed the gut microbiota characteristics in two clinical subgroups: patients with MBD (MBD group) and those without MBD (MM group). Although there was no significant difference in α-diversity and β-diversity (Figure S6A,B), genera such as *Blautia*, *Butyrivibrio*, *Coprococcus*, and *Dorea* of Lachnospiraceae were significantly enriched in the MM group compared with the MBD group (Figure S6C–E). This finding is of particular interest because these genera are important sources of butyrate. Further analysis revealed that the overall abundance of gut butyrate producers and serum butyric acid levels were significantly reduced in MBD patients (*p* < 0.05) (Figures 6A,B and S6F, G), and there was a positive correlation between these two parameters (Figure 6C). Clinical data demonstrated that β-CTX, a marker reflecting OC activity, was markedly elevated in MBD patients (Table S1) and exhibited a negative correlation with the serum butyric acid level and fecal butyrate producers abundance (Figures 6D and S6H,I). However, the PINP level reflecting OB activity showed no significant difference between the two groups. These findings highlight the heterogeneity in the gut microbiota composition among MM patients and suggest a potential association between butyrate deficiency and MBD pathogenesis.

Consequently, we conducted a comprehensive investigation into the effects of *C. butyricum* on bone metabolism in MM mice. Micro-CT reconstruction images and quantitative parameters revealed that mice in group CB and But exhibited attenuated bone loss, mitigated trabecular structural destruction (Figure 6F), and significant improvements in the bone volume fraction (BV/TV), trabecular number (Tb.N), and bone surface/tissue volume (BS/TV), accompanied by reduced trabecular separation (Tb.Sp) and degree of anisotropy (DA) (*p* < 0.05) (Figures 6G and S6J). However, these parameters did not improve in Group B. This finding indicated that the correction of MBD does not solely depend on tumor elimination and may involve a lag effect, thereby underscoring the critical importance of maintaining BMM homeostasis. Furthermore, TRAP staining showed that the quantity and density of OC in the tibia of the CB and But groups were significantly decreased (*p* < 0.01) (Figure 6H,I), and immunohistochemical staining for osteocalcin (OCN) and osteopontin (OPN) indicated partial restoration of OB function (Figure 6H,J). In addition, RANKL levels in the bone marrow and CTXI levels in the serum were markedly decreased in the CB and But groups, while PINP levels showed minimal changes (Figure 6K), similar to what was previously found from clinical data. These results suggest that the improvement of MBD is mainly a result of OC inhibition rather than increased OB formation. In conclusion, *C. butyricum* and butyrate can restore bone metabolic homeostasis in MM, and this process is an essential component in the overall improvement of the BMM.

### 3.7. Butyrate promotes MM cell apoptosis through PPARγ-mediated suppression of the PI3K/AKT signaling pathway

**Figure 7.**
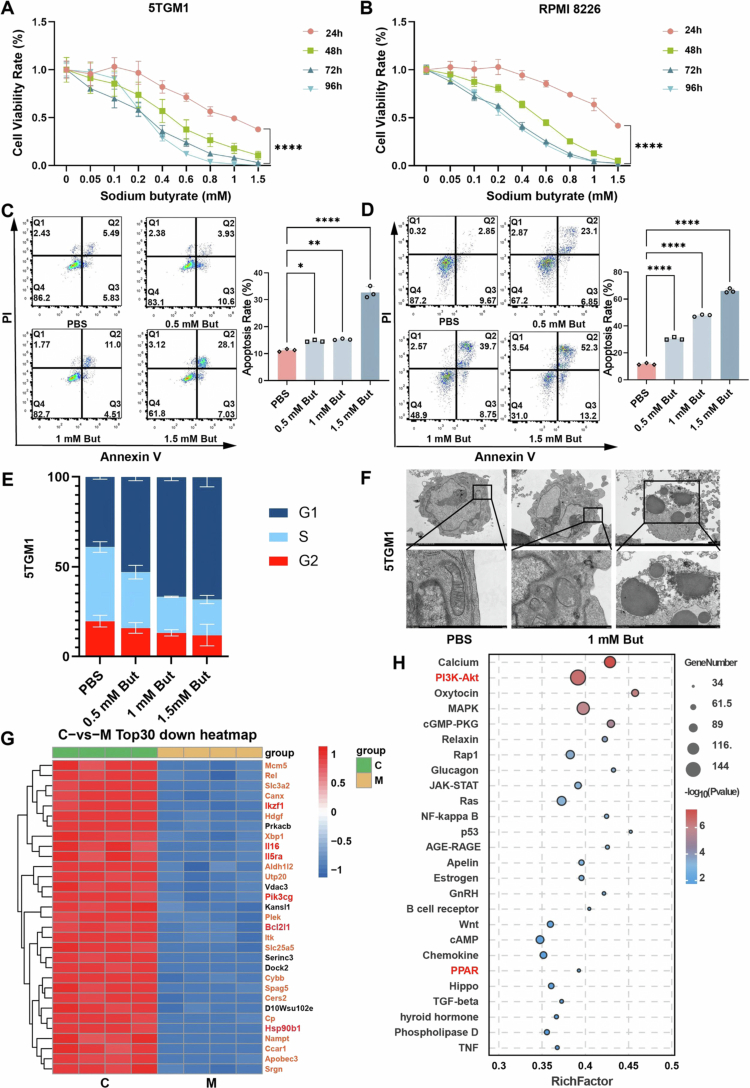
Butyrate promotes MM cell apoptosis through the PI3K/AKT signaling pathway. A) Cell viability of 5TGM1 cells (mice MM cell line) assessed by Cell Counting Kit-8 in response to sodium butyrate treatments at diverse concentrations and time points. B) Cell viability of RPMI 8226 cells (human MM cell line) assessed by a Cell Counting Kit-8 in response to sodium butyrate treatments at diverse concentrations and time points (mean ± SEM, *n* = 6, and ****p < 0.0001 by two-way ANOVA). C) Representative flow cytometry plots and quantification of apoptotic 5TGM1 cells after treatment with various concentrations of sodium butyrate for 24 h. D) Representative flow cytometry plots and quantification of apoptotic RPMI 8226 cells after treatment with various concentrations of sodium butyrate for 24 h (mean ± SEM, *n* = 3, *p < 0.05, **p < 0.01, and ****p < 0.0001 by one-way ANOVA). E) The histogram of 5TGM1-cell cycle distribution detected by flow cytometry (*n* = 3). F) Transmission electron microscope images of apoptotic bodies and abnormal mitochondria in 5TGM1 cells (*n* = 3), scale bar: 500 nm. G) Heatmap of differentially downregulated genes in 5TGM1 cells identified via RNA-Seq analysis (*n* = 4). H) Bubble plot of KEGG gene enrichment analysis in 5TGM1 cells.

In addition to its ability to modulate BMM, we are very curious whether butyrate can exert a direct antitumor effect, as *C. butyricum* or butyrate gavage was found to increase the butyrate level in the bone marrow (Figure S7A). First, we assessed cell viability at 24, 48, 72, and 96 h post-treatment with varying concentrations of sodium butyrate in RPMI 8226 and 5TGM1 cell lines, respectively. The results demonstrated that butyrate inhibited the growth of MM cells in a dose- and time-dependent manner (*p* < 0.0001) (Figure 7A,B). Flow cytometry analysis revealed that even with a short-term (24 h) intervention, sodium butyrate could still promote MM cells apoptosis (*p* < 0.05) in a concentration-dependent manner (Figure 7C,D) and blocked the cell cycle at G1 phase, reducing the proportion of S/G2 phase (Figure 7E). Meanwhile, abnormal cell morphology, mitochondrial swelling, and the formation of apoptotic bodies could be observed under electron microscopy (Figures 7F and S7B). To investigate the molecular mechanism of the antitumor effect of butyrate, transcriptome sequencing was performed on 5TGM1 cells. The genes encoding PI3K catalytic subunit γ (Pik3cg), the antiapoptotic protein Bcl-xl (Bcl2l1), heat shock protein 90β family member 1 (Hsp90b1), interleukin 16 (IL-16), the interleukin 5 receptor α (IL-5ra), and the transcription factors Rel and Ikzf1 were significantly downregulated in group M (Figure 7G). Meanwhile, the expression of the autophagy-related gene CALCOCO1 and the tumor suppressor genes B-cell translocation gene 2 (BTG2), MKNK2, MAGED1, and phospholipase D3 (PLD3) were upregulated (Figure S7C). The results of GO enrichment analysis and GSEA molecular function analysis indicated that cell cycle regulation and DNA repair were significantly inhibited (Figure S7D–G).

**Figure 8.**
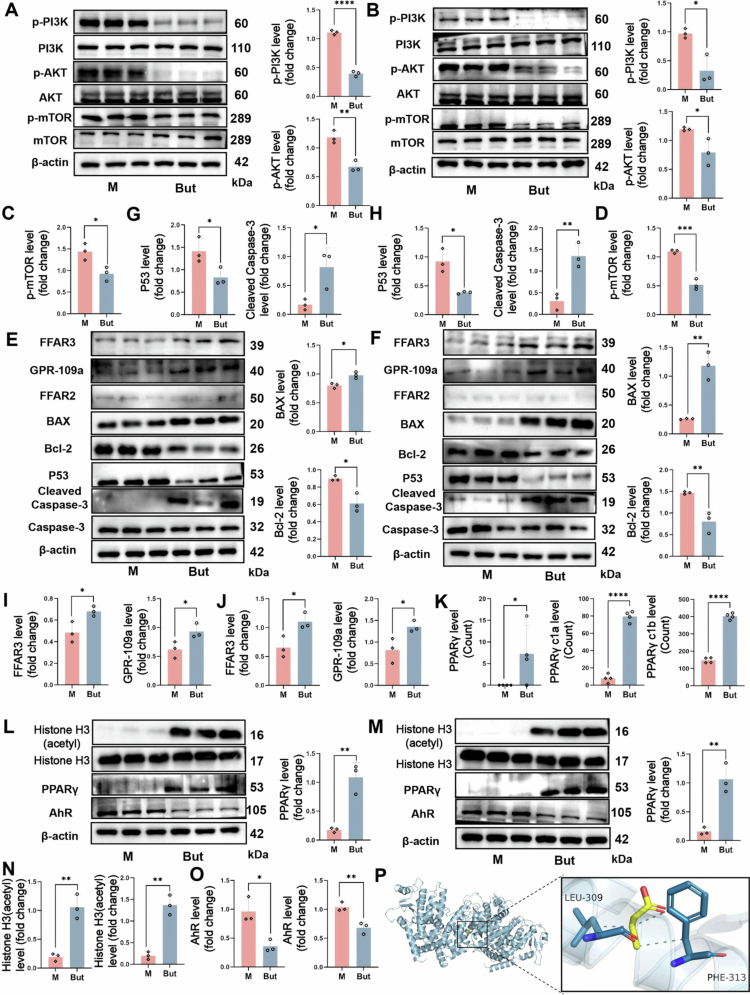
Butyrate inhibits the PI3K/AKT pathway via upregulation of PPARγ. A) and B) Western blot analysis of protein expression levels for PI3K/AKT/mTOR pathway in A) 5TGM1 cells and B) RPMI 8226 cells. C) and D) Quantification of *p*-mTOR protein levels in C) 5TGM1 cells and D) RPMI 8226 cells. E) and F) Western blot analysis of butyrate receptors and apoptosis-related proteins in E) 5TGM1 cells and F) RPMI 8226 cells. G) and H) Quantification of p53 and cleaved Caspase3 protein levels in G) 5TGM1 cells and H) RPMI 8226 cells. I) and J) Quantification of butyrate receptor protein levels in I) 5TGM1 cells and J) RPMI 8226 cells (mean ± SEM, *n* = 3, *p < 0.05, **p < 0.01, and ****p < 0.0001 by Mann‒Whitney *U* test). K) PPARγ differential gene counts in 5TGM1 cells treated with PBS or sodium butyrate. (mean ± SEM, *n* = 4, *p < 0.05, and ****p < 0.0001 by Mann‒Whitney *U* test). L) and M) Acetylated histone, PPARγ, and AhR levels in L) 5TGM1 cells and M) RPMI 8226 cells. N) and O) Quantification of acetylated histone and AhR protein levels in 5TGM1 cells (left) and RPMI 8226 cells (right) (mean ± SEM, *n* = 3, *p < 0.05, and **p < 0.01 by Mann‒Whitney *U* test). P) Molecular docking diagram of butyric acid and PPARγ. HDAC: histone deacetylase; AhR: aryl hydrocarbon receptor; PPARγ: peroxisome proliferator-activated receptor γ.

Notably, KEGG analysis indicated that the PI3K/AKT pathway was significantly affected by butyrate (group M) (Figure 7H). PCR and western blot analysis further confirmed that phosphorylation of key proteins in the PI3K/AKT pathway (PI3K, AKT, and mTOR) was significantly inhibited by butyrate in 5TGM1 (*p* < 0.05) (Figures 8A,C and S8A,B) and RPMI 8226 cells (*p* < 0.05) (Figures 8B,D and S8C). Furthermore, butyrate upregulated the expression of proapoptotic BAX (*p* < 0.05), downstream of PI3K/AKT while suppressing the expression of the antiapoptotic protein BCL-2 (*p* < 0.05), resulting in disruption of mitochondrial membrane stability and subsequent promotion of the expression of the apoptotic marker cleaved caspase-3 (*p* < 0.05) (Figure 8E–H). Meanwhile, the expression of P53 was also suppressed by butyrate (Figure 8E–H), which might be the result of the downregulation of P53 mutants that exhibit negative activity. In addition, we observed the local expansion of tumor cells in the paraspinal bone in MM mice and found that the phosphorylation of PI3K, AKT, and mTOR and the expression of BCL-2 were inhibited in the local tumor tissues of both CB and But groups (Figure S8D), just as the results presented *in vitro*.

Butyrate primarily exerts effects through the activation of G protein-coupled receptor (GPR) or the inhibition of histone deacetylase (HDAC), yet its specific mechanisms in MM remain unclear. We examined the expression of GPR41 (FFAR3), GPR43 (FFAR2), and GPR109A. The western blot results revealed low expression of FFAR2 in MM cells, while GPR109A and FFAR3 expression was upregulated following butyrate intervention (*p* < 0.05) (Figure 8E,F,I,J). However, GPR can activate the PI3K/AKT pathway as a receptor for certain upstream signaling molecules. Thus, the suppression of PI3K/AKT signaling by butyrate in MM cells was not mediated directly by GPRs. Consequently, we investigated alternative mechanisms of butyrate. Western blot demonstrated that butyrate significantly elevated the acetylation level of histone H3 (an indirect indicator of HDAC inhibition activity) in MM cells (*p* < 0.01) (Figures 8L–N and S8E) and localized tumor tissues of 5TGM1 mice (Figure S8F). Although butyrate is known to enhance AhR signaling by increasing aryl hydrocarbon receptor (AhR) expression^18^ or acting as an HDAC inhibitor,^19^ our results did not observe the promoting effect of butyrate on AhR expression in MM cells (Figure 8L,M,O). Notably, transcriptome sequencing indicated the significant upregulation of peroxisome proliferator-activated receptor γ (PPARγ) (*p* < 0.05) (Figure 8K), which was subsequently confirmed by western blot in both 5TGM1 and RPMI 8226 MM cells (*p* < 0.01) (Figure 8L,M). Additionally, molecular docking simulations revealed that butyrate might directly bind to PPARγ (Figure 8P) and act as an activator. We further conducted rescue experiments using GW9226, a potent and selective PPARγ antagonist. The results showed that GW9226 reduced butyrate-induced apoptosis and reversed the changes in the PI3K/AKT pathway (Figure S9A,B). Our findings suggest that butyrate can suppress the PI3K/AKT pathway by up-regulating the expression of PPARγ, which may be related to the inhibition of HDAC. Additionally, apoptosis detection and Western blot analysis revealed that butyrate could act synergistically with BTZ to induce MM cell apoptosis through suppression of the PI3K/AKT pathway (Figure S9C–F), thereby confirming our initial *in vivo* results.

### 3.8. *C. butyricum* and butyrate exert direct antitumor effects in immunodeficient MM mice

To further investigate the direct antitumor effect of *C. butyricum* in MM mice, we established a systemic xenograft MM model using M-NSG mice and RPMI 8226-luc cells. The mice were treated with BTZ and gavaged with solvent (B group), *E. coli* (EC group), *C. butyricum* (CB group), or butyrate (But group) (Figure S10A). Living imaging (Figure S10B,C) and detection of immunoglobulin light chains in serum (Figure S10D) showed that *C. butyricum* or butyrate significantly suppressed MM progression compared with that in the B and EC groups. In addition, HE and CD138 immunohistochemical staining showed that, compared to the M, B, and EC groups, MM cell infiltration in the spleens was significantly reduced in both CB and But groups (Figure S10E–G). These results further demonstrated the antitumor effect of *C. butyricum* in MM mice, which was independent of the adaptive immune system, and highlighted its translational potential.

## 4. Discussion

Despite advancements in treatment, MM remains incurable due to persistent threats of relapse and drug resistance. Therefore, to achieve maximal remission and enhance patients' quality of life is the primary therapeutic objective. Emerging research on the interplay between the gut microbiota, host metabolism, and disease pathogenesis has positioned microbiota modulation as a promising therapeutic strategy in solid tumors.^20^ However, studies exploring its implications in hematological malignancies remain limited. Furthermore, most investigations focus on the detrimental effects of gut microbiota dysbiosis on MM; critical gaps persist in identifying key probiotic species. In this study, we elucidate the relationship between the gut microbiota and BMM dysfunction in MM and, for the first time, demonstrate that *C. butyricum* can suppress MM progression by remodeling the BMM and directly inducing tumor cell apoptosis.

Our analysis of the gut microbiota and metabolites revealed significant depletion of butyrate producers and reduced fecal butyrate levels in newly diagnosed MM patients. This finding is consistent with a previous report that persistent minimal residual disease negativity, a strong predictor of long-term survival, correlates with the abundance of butyrate-producing *Eubacterium hallii* and *Faecalibacterium prausnitzii.* ^21^ ^,^ ^22^ Recently, Shi et al. discovered that butyrate could inhibit the progression of natural killer/T-cell lymphoma by downregulating the JAK-STAT pathway.^23^ Another pancancer study demonstrated that supplementation with a mixture of traditional *Clostridium* species capable of producing butyrate can prevent and even successfully treat colorectal cancer and melanoma in mice.^24^ Furthermore, *C. butyricum* has shown some efficacy in clinical trials, and Tomita Y et al. found that it significantly improved the outcome of lung cancer patients receiving immune checkpoint inhibitors.^25^ In summary, supplementation with *C. butyricum* or butyrate may inhibit MM progression.

To investigate whether the alterations of gut microbiota observed in clinical research are involved in the pathogenesis of MM, we conducted the FMT experiment and found that the fecal microbiota from healthy donors significantly reduced the tumor burden and restored intestinal barrier integrity. These effects might correlate with elevated butyrate levels. Consistent with our findings, transplantation of healthy fecal bacteria could increase butyrate producers and SCFA levels in patients with metabolic syndrome.^26^ Butyrate-mediated inhibition of disease progression by FMT was also observed in acute myeloid leukemia and lymphoma.^10^ ^,^ ^27^ These data collectively demonstrate that microbiota reconstitution, particularly enrichment of butyrate producers, may improve MM through metabolite-dependent mechanisms. PIs have been established as the cornerstone of MM therapy, and BTZ, being one of the most representative agents, is widely adopted in clinical practice. Therefore, to provide further evidence for clinical application, we investigated the effects of *C. butyricum* and butyrate in combination with BTZ. As we hypothesized, *C. butyricum* and butyrate increased the efficacy of BTZ. In addition, adverse reactions such as nausea, diarrhea, and constipation can be observed during BTZ treatment in the clinic. Lenalidomide and dexamethasone may aggravate gastrointestinal symptoms.^28^ Furthermore, owing to plasma cell dysfunction and treatment-induced neutropenia, the risk of infections in MM patients is significantly increased, leading to the prevalent use of antibiotics.^29^ ^,^ ^30^ Therefore, it is essential to mitigate gastrointestinal complications and restore gut microbiota balance. Butyrate was found to strengthen the intestinal barrier by upregulating the expression of claudin-1, ZO-1, occludin, and Mucin2.^31^ Our results further indicated that *C. butyricum* and butyrate could reduce the gastrointestinal damage induced by MM and BTZ.

Chronic inflammation driven by interactions between malignant plasma cells and the BMM serves as a key driver of MM progression and therapeutic resistance. The BMM comprises noncellular components (e.g., cytokines and chemokines) and cellular constituents, including immune cells, osteoblasts, osteoclasts, and bone marrow stromal cells.^32^ We found that the fecal microbiota from healthy donors could significantly reduce the accumulation of Th17 cells and the production of IL-17 in the bone marrow of mice. Previous studies have shown that murine gut-resident *segmented filamentous bacteria* and human-derived *Bifidobacterium adolescentis* are both inducers of Th17 differentiation.^33^ ^,^ ^34^ While physiological Th17 responses mediate mucosal protection, pathologically Th17 activation by gut microbes predisposes to inflammatory disorders such as IBD and rheumatoid arthritis.^35^ Of particular relevance to MM, clinical studies have demonstrated that Th17 cells and IL-17 are elevated in the bone marrow, which correlates with exacerbated osteolysis and MM cell proliferation.^9^ ^,^ ^36^ These findings identify Th17 cells as an important link between the gut microbiota and MM. In addition, detecting changes in macrophage subsets would provide a clearer depiction of the immune microenvironment composition and reveal the mechanism underlying their alterations in the FMT-MM group.

A recent study demonstrated that *Prevotella heparinolytica* promotes Th17 cell migration in Vκ*MYC mice, thereby accelerating MM progression.^37^ Intriguingly, our findings revealed that *C. butyricum* and butyrate suppressed intestinal Th17 differentiation, bone marrow Th17 infiltration, and IL-17 secretion. Prior research has established that microbiota-derived SCFAs modulate the Th17/Treg imbalance in autoimmune pathologies by attenuating IL-17A production.^38^ Specifically, butyrate can upregulate T-bet expression to enhance Th1 polarization while downregulating RORγt to inhibit Th17 commitment.^39^ Hence, the migration of gut-derived Th17 cells may represent a pivotal mechanism through which *C. butyricum* modulates the BMM via the gut‒bone axis. Furthermore, chronically elevated inflammatory mediators in the BMM fuel MM progression through pleiotropic mechanisms.^7^ SCFAs are known to orchestrate the gut microbiota-mediated regulation of systemic inflammation via multiple signaling pathways.^40^ For instance, GPR43-deficient murine models of colitis, arthritis, and asthma exhibit exacerbated inflammatory responses.^41^ Butyrate can inhibit HDAC to blunt LPS-driven M1 macrophage polarization and reduce proinflammatory cytokine output.^42^ Collectively, *C. butyricum* may restore BMM balance by inhibiting the differentiation and migration of Th17 cells and alleviating systemic inflammation.

Notably, severe bone metabolic imbalance represents a hallmark manifestation of BMM dysregulation in MM. Over 80% of patients develop a spectrum of skeletal complications, including osteoporosis, hypercalcemia, osteolytic lesions, and pathologic fractures.^43^ Evidence links bone marrow Th17/IL-17 axis hyperactivity to OC activation and osteolytic progression in MM.^9^ Inflammatory cytokines act as osteoclast-activating factors to exacerbate MBD through excessive activation of the RANKL‒RANK signaling pathway.^44^ Our research indicates that *C. butyricum* can restore the imbalance of bone metabolism by inhibiting the secretion of IL-17 and alleviating bone marrow inflammation. Supporting this, probiotics could suppress systemic inflammation and bone loss via TNFα/RANKL/IL-17 suppression in sex steroid-deficient mice.^45^ He et al. reported a depletion of butyrate producers in rheumatoid arthritis patients and demonstrated that butyrate inhibited Th17 polarization and blocked OC-driven bone destruction.^46^ In summary, *C. butyricum* and butyrate disrupt the pathogenic crosstalk between intestinal Th17 cells and bone metabolism, thereby alleviating MBD.

In addition to regulating the BMM, our study revealed that butyrate directly induces MM cell apoptosis by modulating BCL2 family proteins through PI3K/AKT pathway suppression. This finding is consistent with the previous study that butyrate could reduce MM cell viability in a concentration-dependent manner.^47^ Recent clinical evidence has demonstrated the antitumor activity of AKT or BCL-2 inhibitors in subsets of MM patients.^48^ ^,^ ^49^ Furthermore, transcriptomic profiling identified a significant downregulation of PIK3CG encoding the PI3K catalytic subunit, in MM cells treated with butyrate. PIK3CG expression is bone marrow restricted, and its inhibition can block AKT signaling and suppress acute myeloid leukemia.^50^ These findings highlight the potential of targeting PI3K/AKT signaling in MM pathogenesis. Regarding upstream mechanisms, our study revealed that butyrate upregulates the expression of PPARγ. Garcia Bates TM et al. discovered that overexpression of PPARγ could inhibit the proliferation of MM cells and induce apoptosis by downregulating MCL-1.^51^ In addition, PPARγ activation promotes the expression of its target gene PTEN to inhibit the PI3K/AKT/mTOR signaling, thereby suppressing the proliferation of breast cancer cells and the tumorigenicity and metastasis of cervical, glioblastoma, and liver cancer stem cells.^52^ ^,^ ^53^ In our study, the rescue of butyrate-induced apoptosis by PPARγ inhibitors at least explains the critical role of PPARγ in the antitumor activity of butyrate. HDAC inhibitors can induce acetylation of the upstream regulatory factor C/EBPα of PPARγ.^54^ Notably, an increase in histone acetylation levels induced by butyrate was also observed in our study. Therefore, the anti-MM effects of butyrate may arise from its HDAC inhibitory activity and the suppression of PI3K/AKT pathway signaling mediated by PPARγ.

Our study does have certain limitations. First, although oral administration is relatively safe, further exploration is necessary to clarify whether butyrate affects nontumor cells through cytotoxicity or functional regulation. The effects of butyrate on other cellular components of BMM also require further investigation. Second, the specific mechanisms of butyrate's role in regulating Th17 cell differentiation and migration can be further verified and explored in genetically engineered or immune-deficient mouse models. Furthermore, the potential therapeutic synergy of *C. butyricum* or butyrate combined with standard treatment remains to be validated in clinical trials. Finally, while *C. butyricum* demonstrates beneficial effects on immune and inflammation modulation in MM. The contributions of other probiotic strains to these processes remain unclear. These concerns will be the primary focus of future research.

## 5. Conclusions

**Figure 9.**
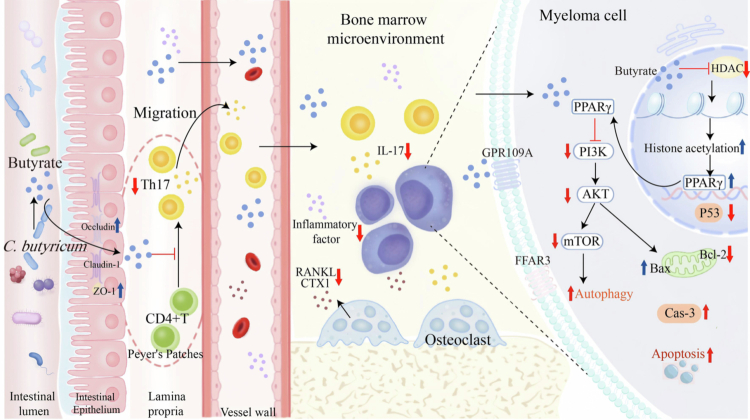
Schematic diagram of the mechanism by which *C. butyricum* exerts antitumor effects in MM.*C. butyricum* repairs the intestinal barrier by restoring the gut microbiota composition and producing a large amount of butyrate. At the same time, it inhibits the differentiation of Th17 cells in intestinal Peyer's patches and their migration to the bone marrow, thereby alleviating bone marrow inflammation and restoring the balance of bone metabolism. These effects remodel the bone marrow microenvironment to suppress MM progression and alleviate myeloma bone disease. Additionally, butyrate can also upregulate the expression of PPARγ by inhibiting HDAC activity, thereby inhibiting the PI3K/AKT pathway to promote the apoptosis and autophagy of myeloma cells.

Our research clarifies two relatively independent mechanisms by which *C. butyricum* alleviates MM progression. On the one hand, *C. butyricum* suppress bone marrow inflammation and bone metabolic imbalance, thereby remodeling the BMM by inhibiting Th17 cells; on the other hand, butyrate directly induce malignant plasma cell apoptosis via the PPARγ/PI3K/AKT pathway. In addition, *C. butyricum* can protect the intestinal barrier and alleviate intestinal damage caused by MM and chemotherapy drugs. In conclusion, probiotics represent a clinically safe and mild adjuvant therapy, particularly relevant for MM management given the higher prevalence among older adults. Our study provide a multiperspective understanding of the role of the gut microbiota in MM and establish a scientific basis for improving the clinical outcomes in hematological malignancies by manipulating the gut microbiota (Figure 9).

## Supplementary Material

Supplementary material Supporting Information1.docx

Supplementary material Supporting Information2.docx

Supplementary material Supplementary Material

Supplementary material Supplementary Material

## Data Availability

The raw 16S rRNA sequencing data generated for the human and murine experiments and 5TGM1 cell RNA-seq data have been deposited in the NCBI Sequence Read Archive under the accession numbers PRJNA1244567, PRJNA1248389, PRJNA1248399, and PRJNA1380903.
