# Supplementary material for: Clostridium butyricum alleviates multiple myeloma by remodeling the bone marrow microenvironment and inhibiting PI3K/AKT pathway through the gut‒bone axis
Source: Gut Microbes. 2026 Jan 2;18(1):2609455. doi: 10.1080/19490976.2025.2609455 (PMC12773645; doi:10.1080/19490976.2025.2609455)
Supplement: Supplementary material — Supporting Information1.docx [file KGMI_A_2609455_SM8872.docx]

**Table S1. Primer sequences used in real-time quantitative RT-PCR**

| Gene | Species | Forward | Reverse |
| --- | --- | --- | --- |
| *mTOR* | mouse | ATGCAGCTGTCCTGGTTCTC | AATCAGACAGGCACGAAGGG |
| *AKT* | mouse | CTGAGATTGTGTCAGCCCTG | CACAGCCCGAAGTCTGTGATCTTA |
| *JAK2* | mouse | TGTCTTACCTCTTTGCTCAGTGGCG | CAATGACATTTTCTCGCTCGACAGC |
| *TLR4* | mouse | GGGCCTAAACCCAGTCTGTTTG | GCCCGGTAAGGTCCATGCTA |
| *HIF-1α* | mouse | TGACTGTGCACCTACTATGTCACTT | GGTCAGCTGTGGGTAATCCACTC |
| *NLRP3* | mouse | ATTACCCGCCCGAGAAAGG | TCGCAGCAAAGATCCACACAG |
| *iNOS* | mouse | GAAAACCCCAAGTGCCGTTC | TGATCTTCACGTGGTGTGGG |
| *β-Actin* | mouse | GATGTATGAAGGCTTTGGTC | TGTGCACTTTTATTGGTCTC |
| *CB* | mouse | AATTACTCTGTAATGGAGGAAGCCA | ATGAGATGCAACCTCGCGAGAGTGA |
| *16s* | mouse | ACTCCTACGGGAGGCAGCAGT | TATTACCGCGGCTGCTGGC |

*CB:*Clostridium butylicum.

**Table S2. Antibodies used in the article**

| Target antigen | Vendor | Catalog# | Concentration |
| --- | --- | --- | --- |
| anti-β-Actin antibody | Cell Signaling  Technology | 4967S | 1:1,000 |
| anti-PI3K antibody | Abcam | ab151549 | 1:2,000 |
| anti-p-PI3K antibody | Abcam | ab278545 | 1:2,000 |
| anti-AKT antibody | Cell Signaling  Technology | 9272S | 1:20,00 |
| anti-p-AKT antibody | Cell Signaling  Technology | 4060S | 1:2,000 |
| anti-mTOR antibody | Cell Signaling  Technology | 2983S | 1:1,000 |
| anti-p-mTOR antibody | Cell Signaling  Technology | 5536T | 1:1,000 |
| anti-Bax antibody | Cell Signaling  Technology | 2772 | 1:1,000 |
| anti-Bcl-2 antibody | Cell Signaling  Technology | 3498 | 1:1,000 |
| anti-p53 (1C12) antibody | Cell Signaling  Technology | 2524 | 1:1,000 |
| anti-GPR109A antibody | Bioss | bs-10079R | 1:2,000 |
| anti-FFAR3 antibody | Abclonal | A12636 | 1:1,000 |
| anti-FFAR2 antibody | proteintech | 19952-1-AP | 1:1,000 |
| anti-Caspase 3 antibody | Abcam | ab32351 | 1:2,000 |
| anti-Cleaved Caspase 3 antibody | Cell Signaling  Technology | 9661 | 1:1,000 |
| anti-Histone H3 antibody | Cell Signaling  Technology | 4499s | 1:1,000 |
| anti-Histone H3(acetyl) antibody | Abcam | ab308373 | 1:1,000 |
| anti-PPAR-γ antibody | Cell Signaling | 2435 | 1:1,000 |
| anti- AhR antibody | Technology | 28727-1 | 1:500 |
| anti-Claudin-1 antibody | Abcam | ab180158 | 1:2,000 |
| anti-Occludin antibody | Proteintech | 66378-1 | 1:5,000 |
| anti-ZO-1 antibody | Servicebio | GB111402 | 1:500 |
| Anti-Mouse secondary antibody | Proteintech | SA00001-1 | 1:2,000 |
| Anti-Rabbit secondary antibody | Proteintech | SA00001-2 | 1:2,000 |
| FITC(green) conjugated Goat Anti-Rabbit IgG | Servicebio | GB22303 | 1:100 |
| Cy3(red) conjugated Goat Anti-Rabbit IgG | Servicebio | GB21303 | 1:100 |

**Table S3. Antibodies used in the flow cytometry**

| Target antigen | Vendor | Catalog# |
| --- | --- | --- |
| PE Anti-Mouse CD4 Antibody[GK1.5] | Elabscience | E-AB-F1097D |
| FITC Anti-Mouse CD3 Antibody[17A2] | Elabscience | E-AB-F1013C |
| APC Anti-Mouse CD8a Antibody[53-6.7] | Elabscience | E-AB-F1104E |
| FITC Anti-Mouse F4/80 Antibody[CI:A3-1] | Elabscience | E-AB-F0995C |
| PE Anti-Mouse/Human CD11b Antibody[M1/70] | Elabscience | E-AB-F1081D |
| APC Anti-Mouse IL-17Antibody[TC11-18H10.1] | Elabscience | E-AB-F1199E |
| FITC Anti-Mouse CD19 Antibody[1D3] | Elabscience | E-AB-F0986C |
| PE Anti-Mouse CD45R/B220 Antibody | Elabscience | E-AB-F1112D |
| PE Anti-Mouse CD4 Antibody[GK1.5] | Elabscience | E-AB-F1097D |
| BD Pharmingen™ PerCP-Cy™5.5 Rat Anti-Mouse IL-17A | BD | 560666 |

**Table S4. Key Resources**

| Chemicals, Peptides, and Recombinant Proteins | | |
| --- | --- | --- |
| Intracellular Fixation/  Permeabilization Buffer Kit | Elabscience | E-CK-A109 |
| Cell Staining Buffer | Elabscience | E-CK-A107 |
| 10× RBC Lysis/Fixation Solution | Elabscience | E-CK-A106 |
| Brefeldin A | Merck | B5936 |
| foetal bovine serum(FBS) | Gibco | 16000-044 |
| Sodium butyrate | Sigma-Aldrich | 156-54-7 |
| GW9662 | MedChemExpress | HY-16578 |
| TRIzol reagent | Takara | 9109 |
| RPMI Medium 1640 | Gibco | 11875093 |
| Dulbecco's Modified Eagle Medium(DMEM) | Gibco | 11965500BT |
| Penicillin and Streptomycin | Beyotime Biotechnology | C0222 |
| radioimmunoprecipitation assay (RIPA) | Solarbio | R0020 |
| polyvinylidene difluoride (PVDF) | Merck | ISEQ00010 |
| Tween-20 | Solarbio | T8220 |
| ultrasensitive chemiluminescence substrate | UElandy | S6009M |
| Critical Commercial Assays | | |
| QIAamp Faecal DNA Rapid Purification Mini Kit | QIAGEN GmbH | 51604 |
| PrimeScript™RT Reagent Kit with gDNA Eraser | Takara | RR047A |
| TB Green®Premix Ex Taq™ II | Takara | RR820A |
| Genomic DNA Extraction Kit | MP Biomedicals | 116564384 |
| BCA Protein Quantification Kit | Thermo Fisher | 23227 |
| IL6 ELISA Kit | Boster | EK0411 |
| IL-1 β ELISA Kit | Boster | EK0394 |
| TNF-α ELISA Kit | Boster | EK0527 |
| IL-17 ELISA Kit | FineTest | EGP0028 |
| RANKL/TNFSF11 ELISA kits | Boster | EK0843 |
| CTXI ELISA Kit | FineTest | EM0960 |
| PINP ELISA Kit | FineTest | EM0481 |
| IgG2b ELISA Kit | FineTest | EM2086 |
| Cell Counting Kit-8 | Miracle | MRC-C23009 |
| Annexin V-FITC/PI Apoptosis Kit | Coolaber | SK2070 |
| Experimental Models: Organisms/Strains | | |
| *Clostridium butylicum* | Segregation | N/A |
| C57BL/KaLwRij | GemPharmatech Co, Ltd | N/A |
| 5TGM1-luc | Donation |  |
| RPMI 8226 | Shanghai Model Organisms |  |
| HT-29 | Shanghai Model Organisms |  |
