# Supplementary figures and images for: Clostridium butyricum alleviates multiple myeloma by remodeling the bone marrow microenvironment and inhibiting PI3K/AKT pathway through the gut‒bone axis
Source: Gut Microbes. 2026 Jan 2;18(1):2609455. doi: 10.1080/19490976.2025.2609455 (PMC12773645; doi:10.1080/19490976.2025.2609455)

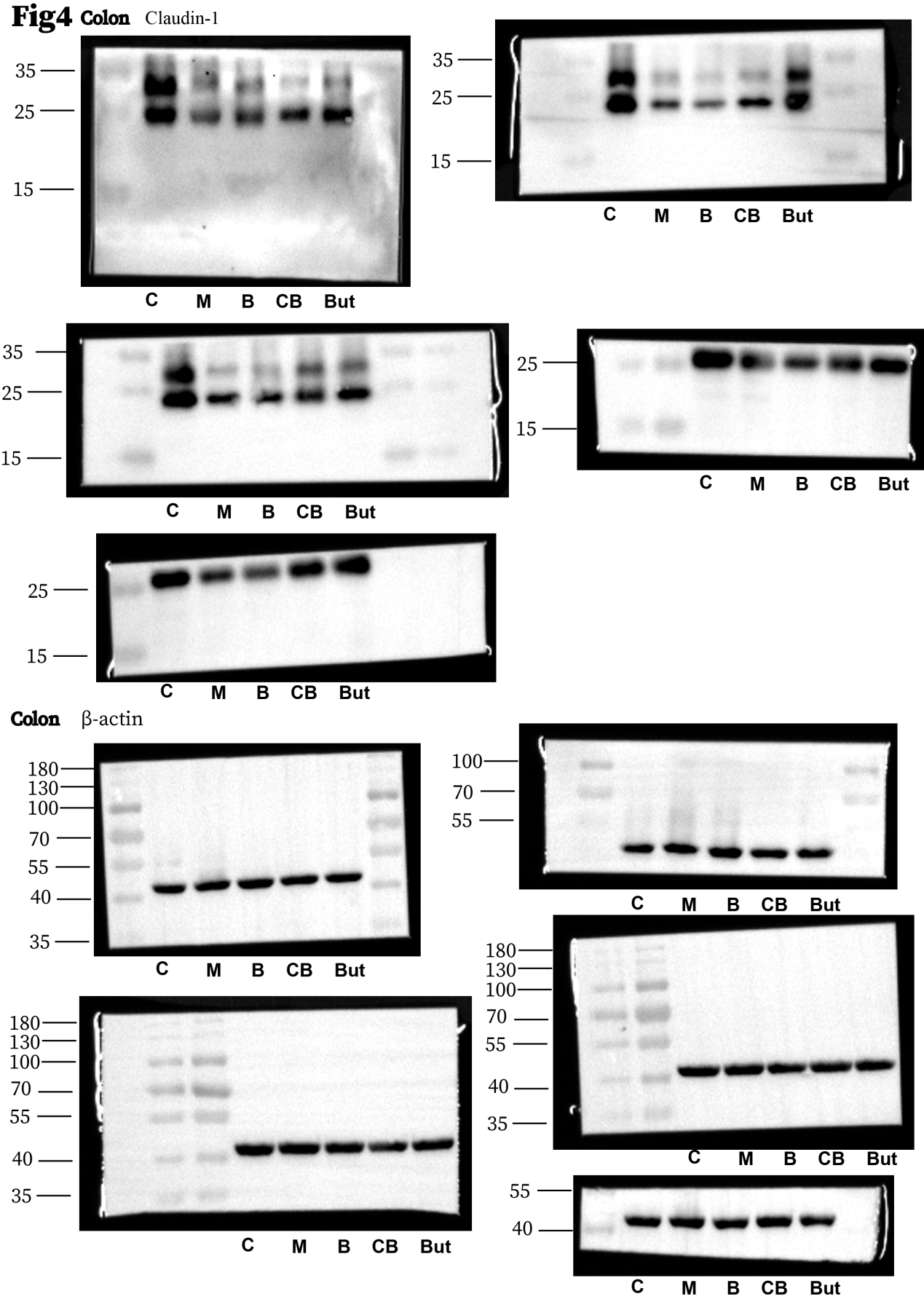

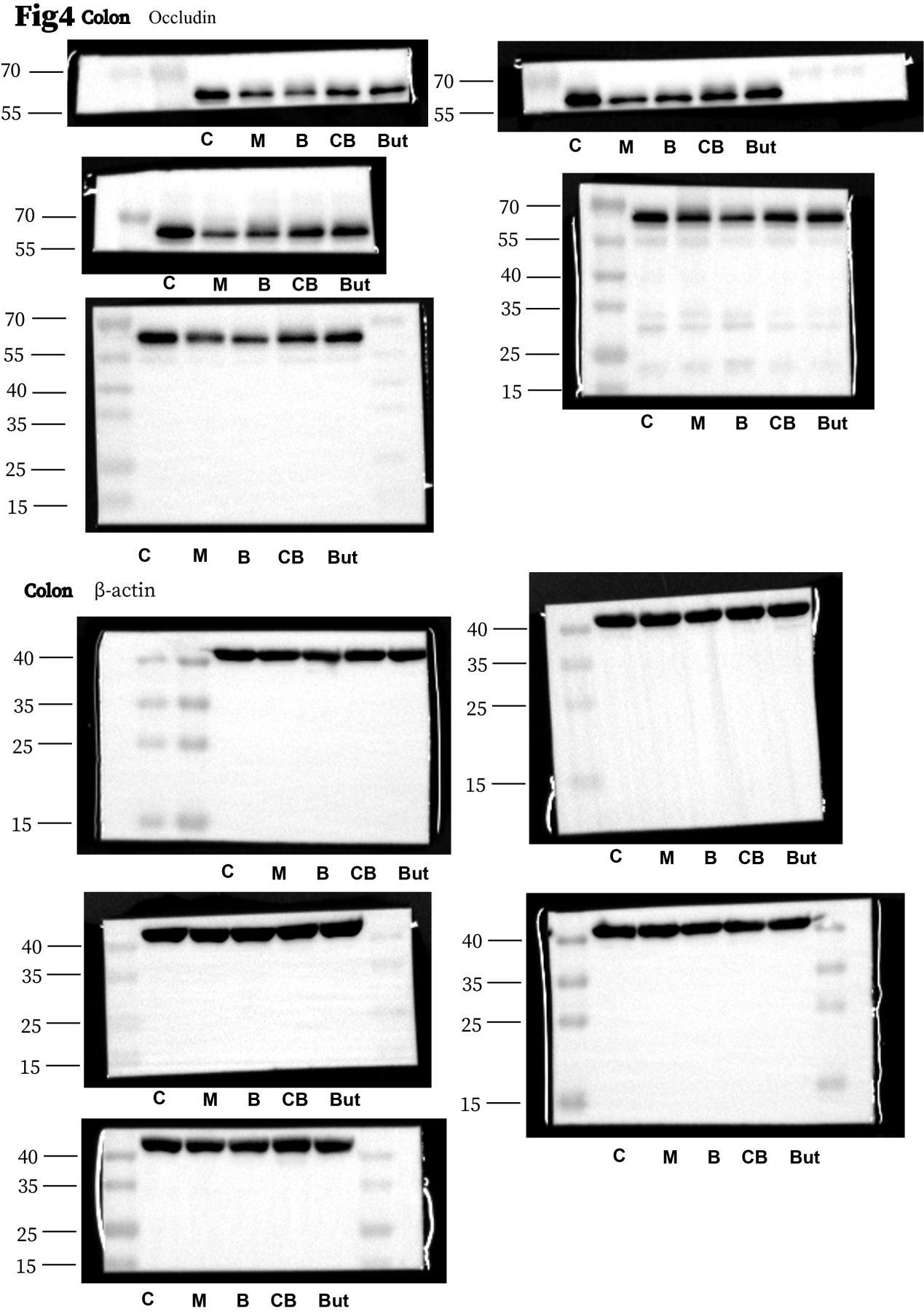


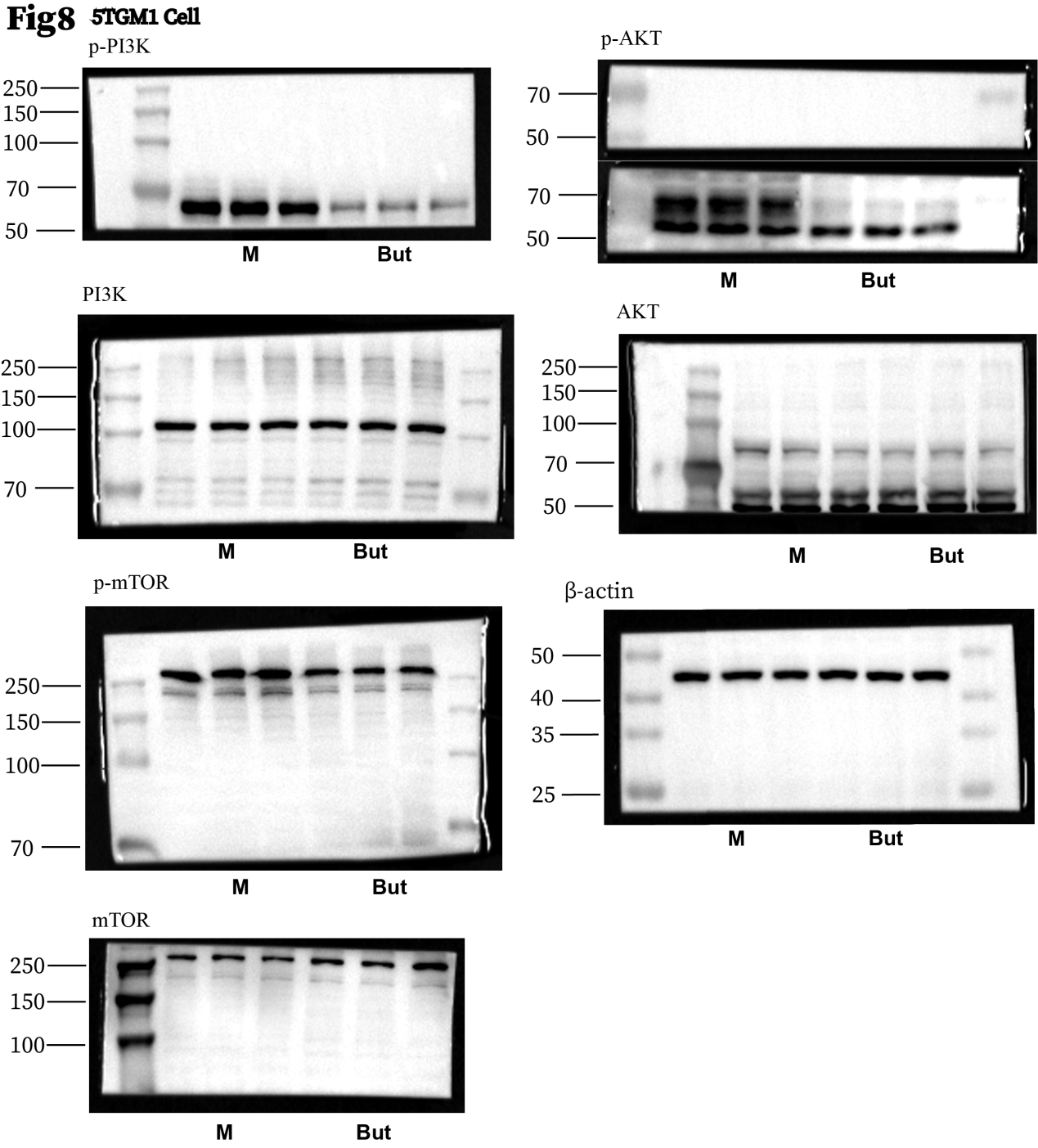

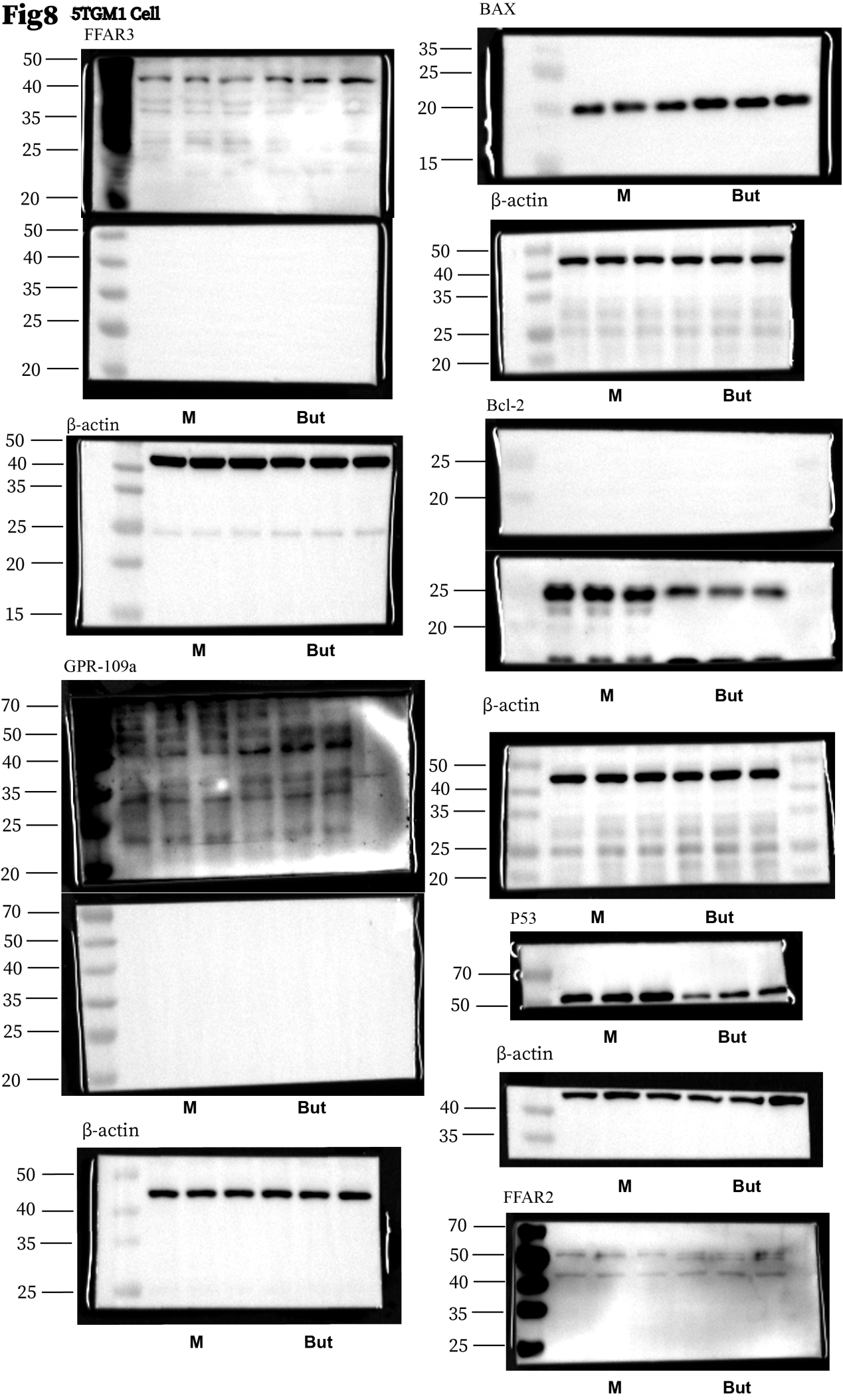


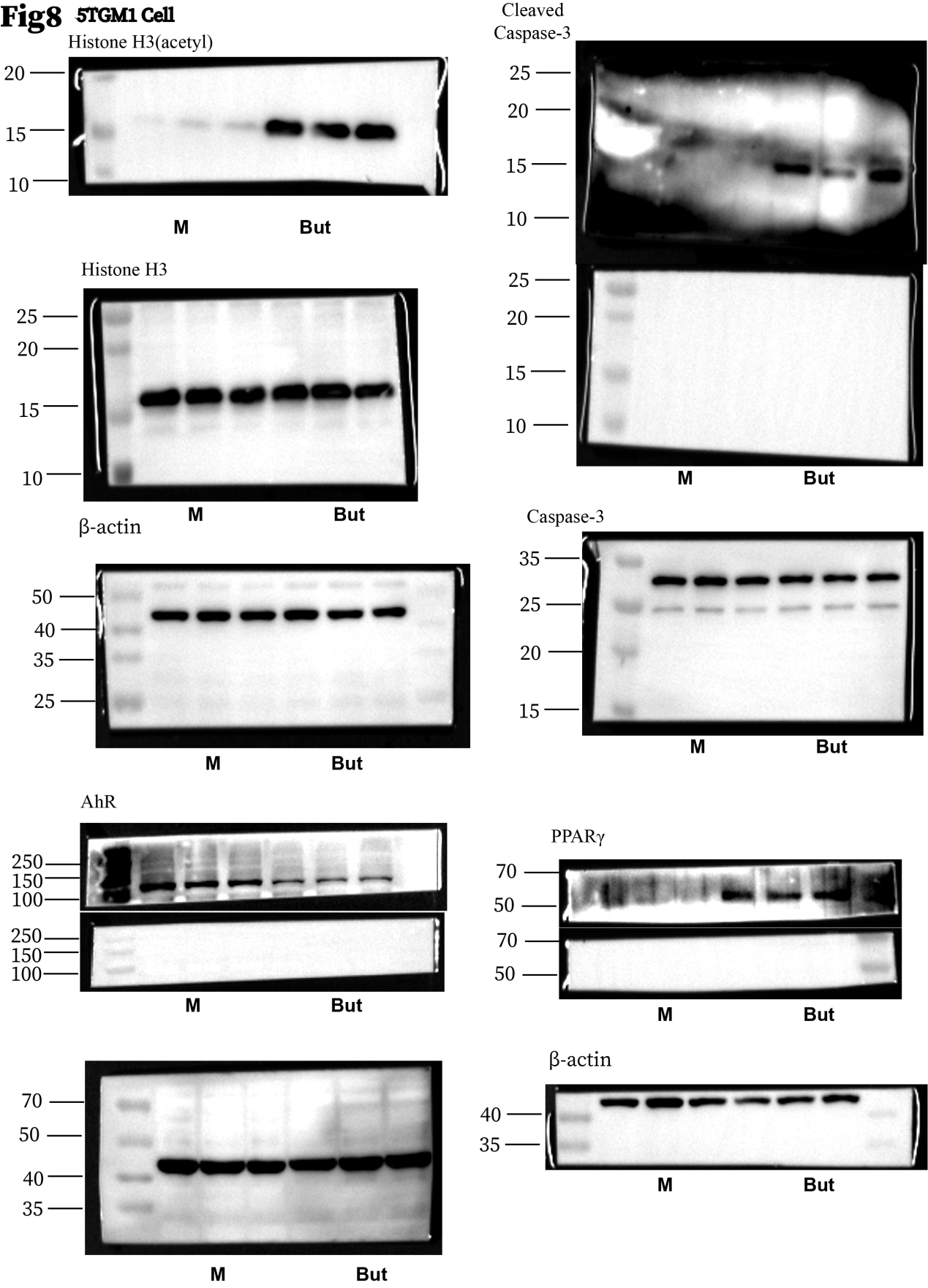


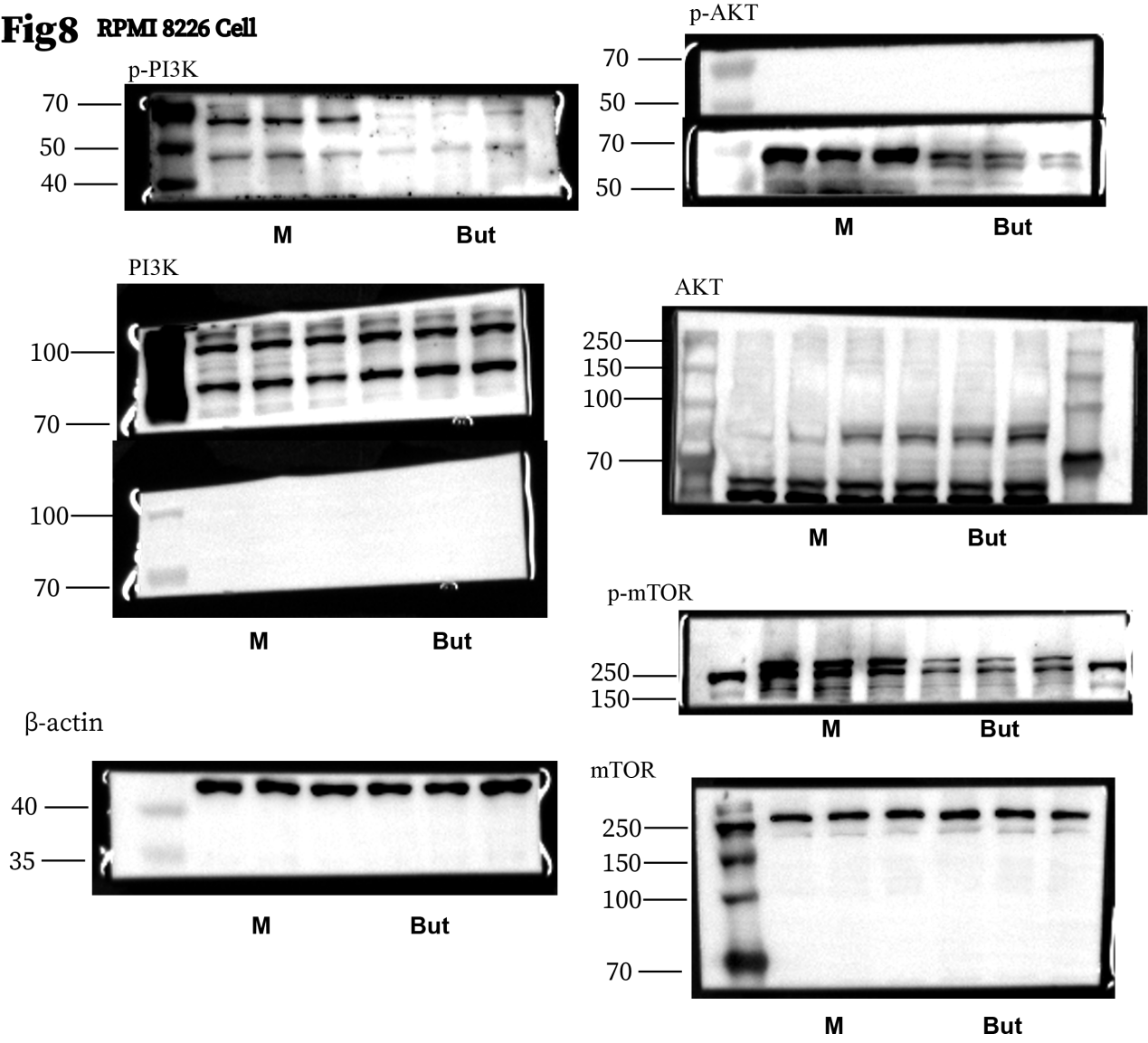

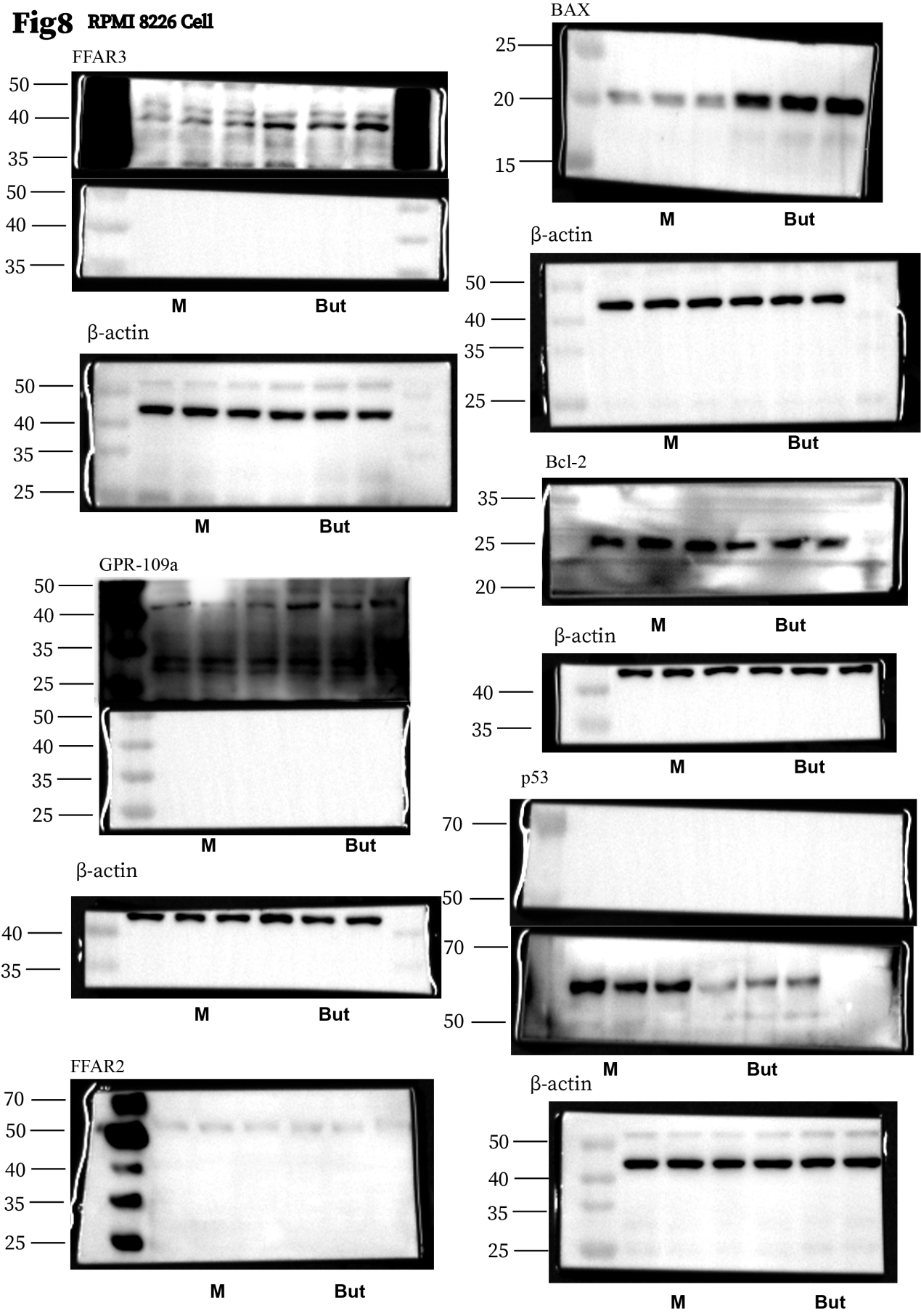

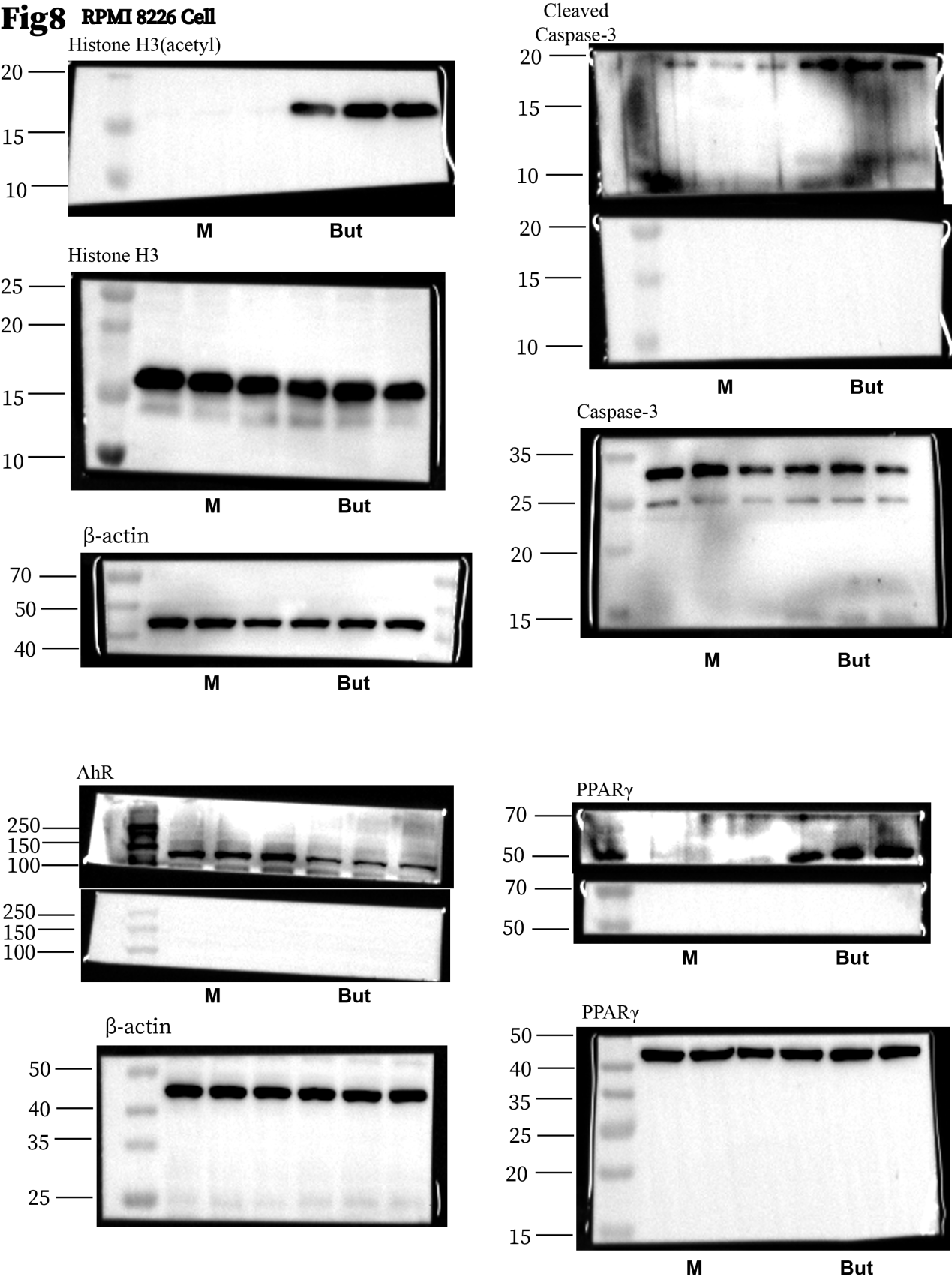


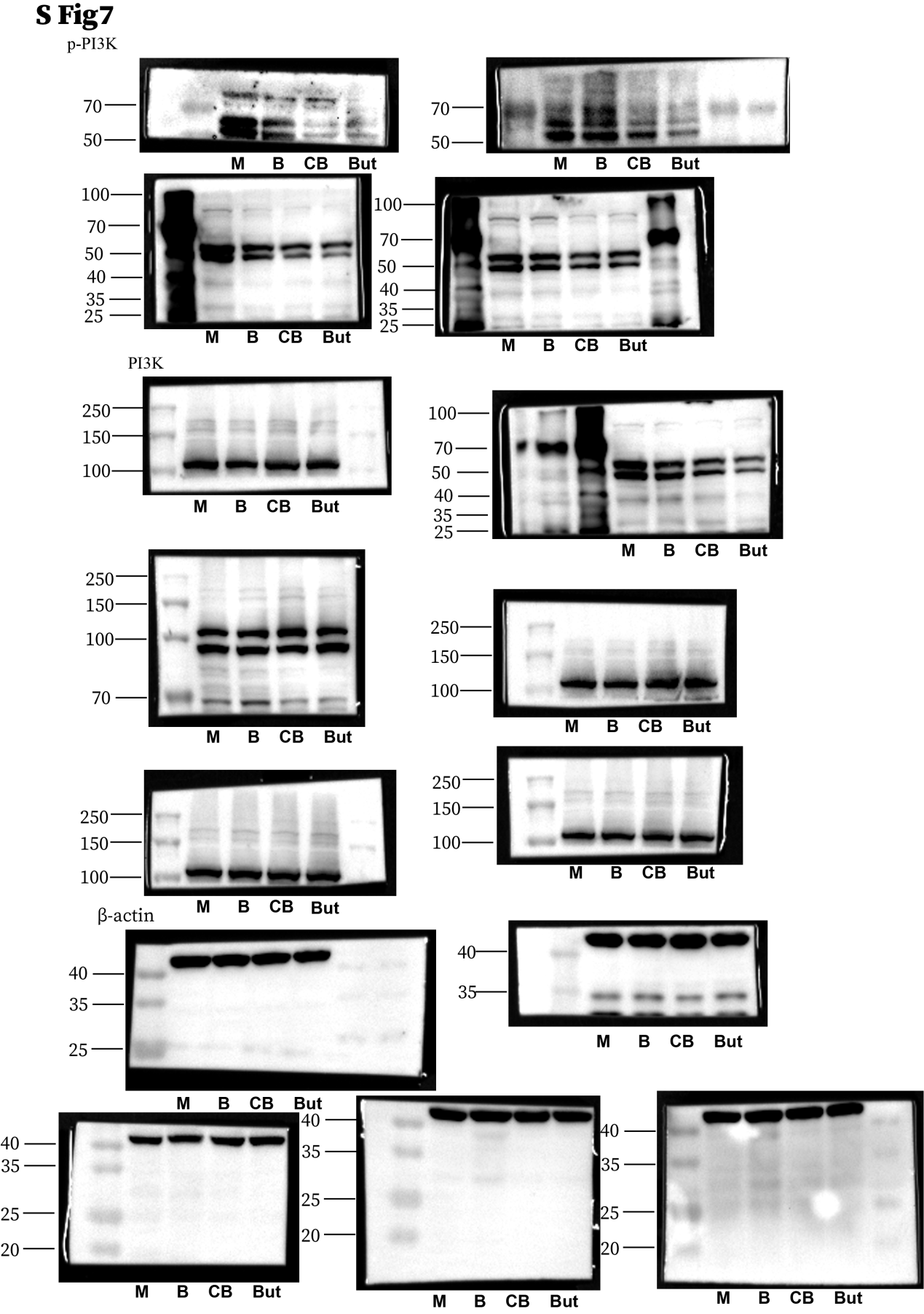

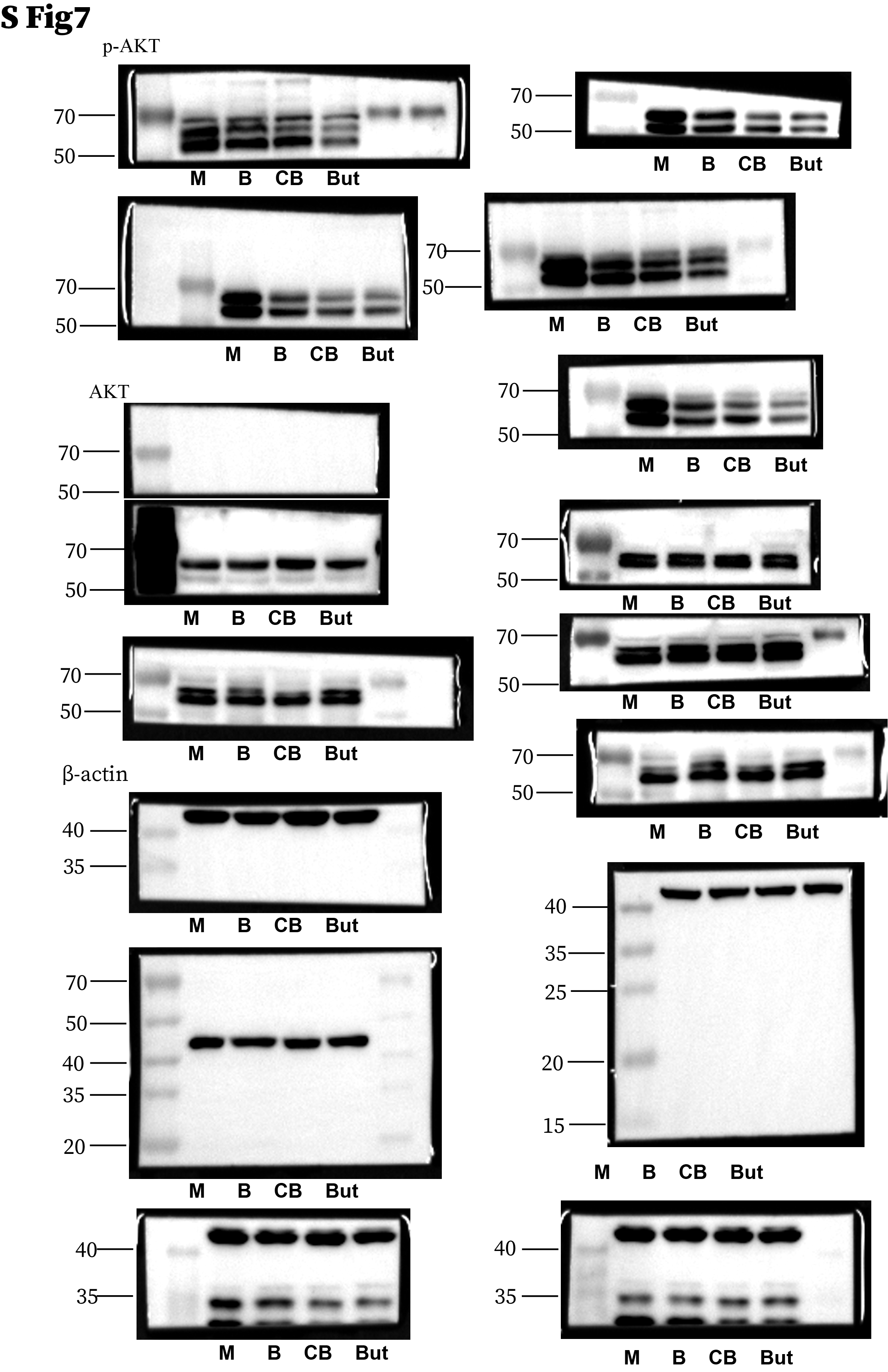

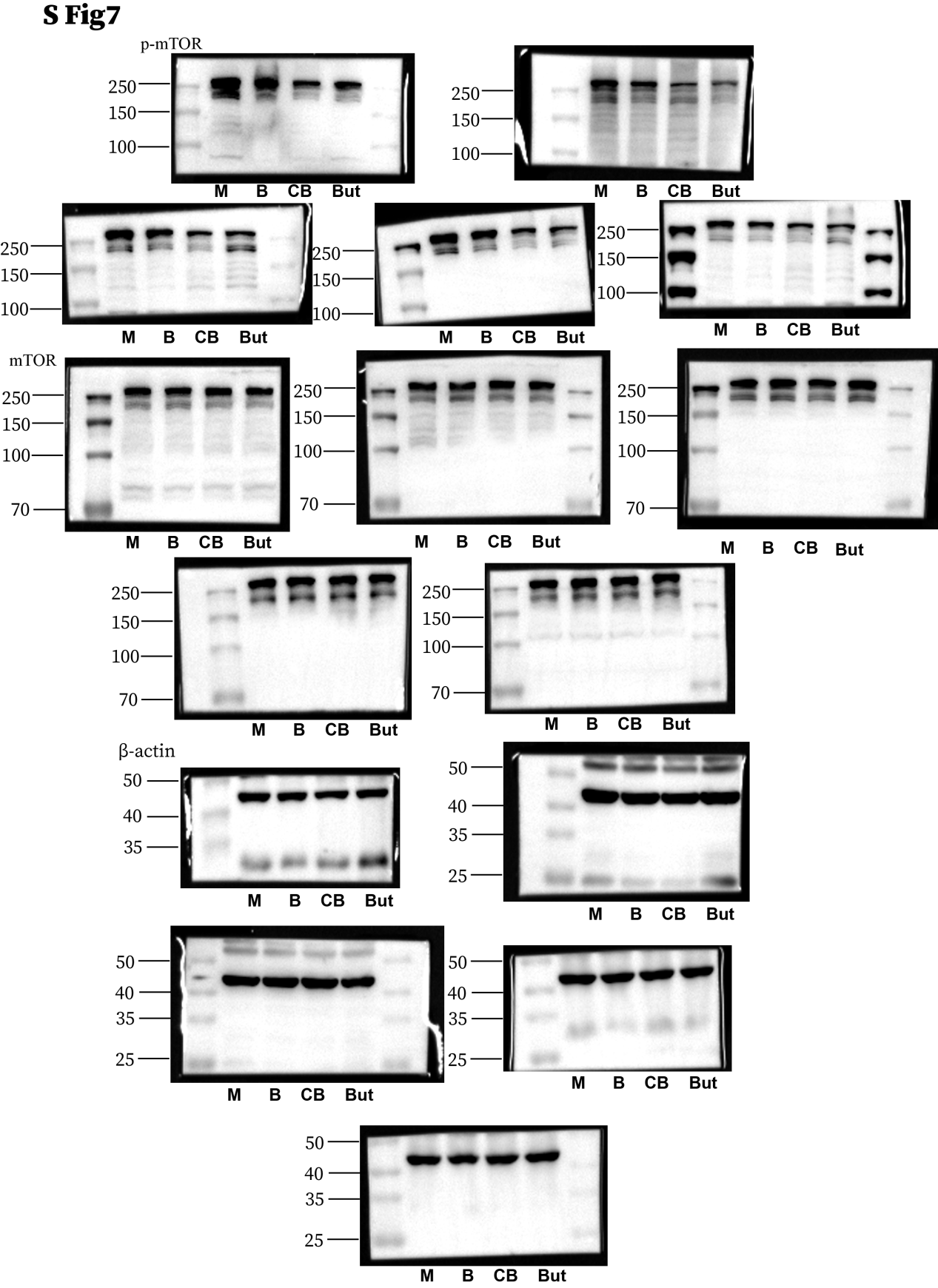

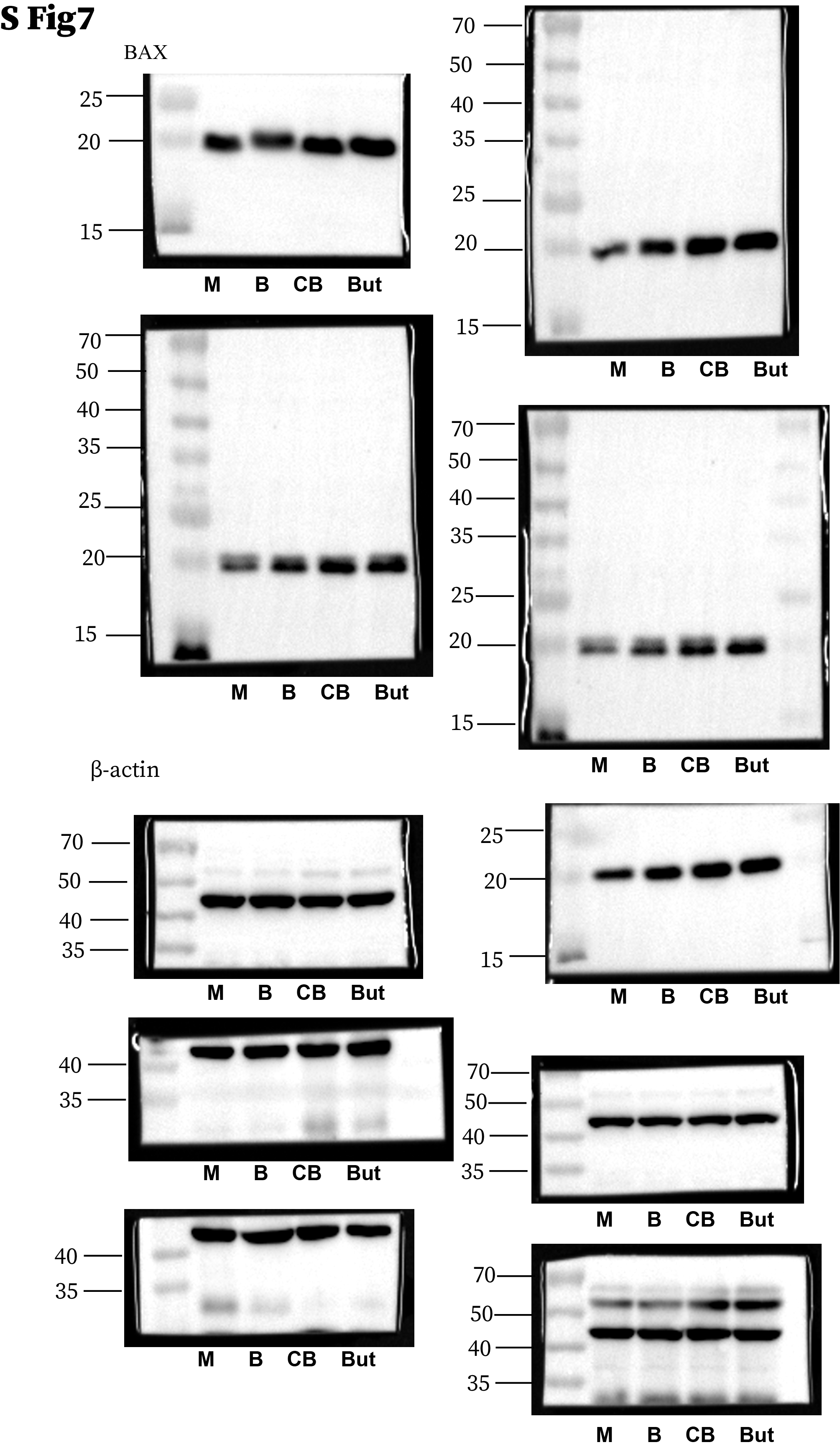

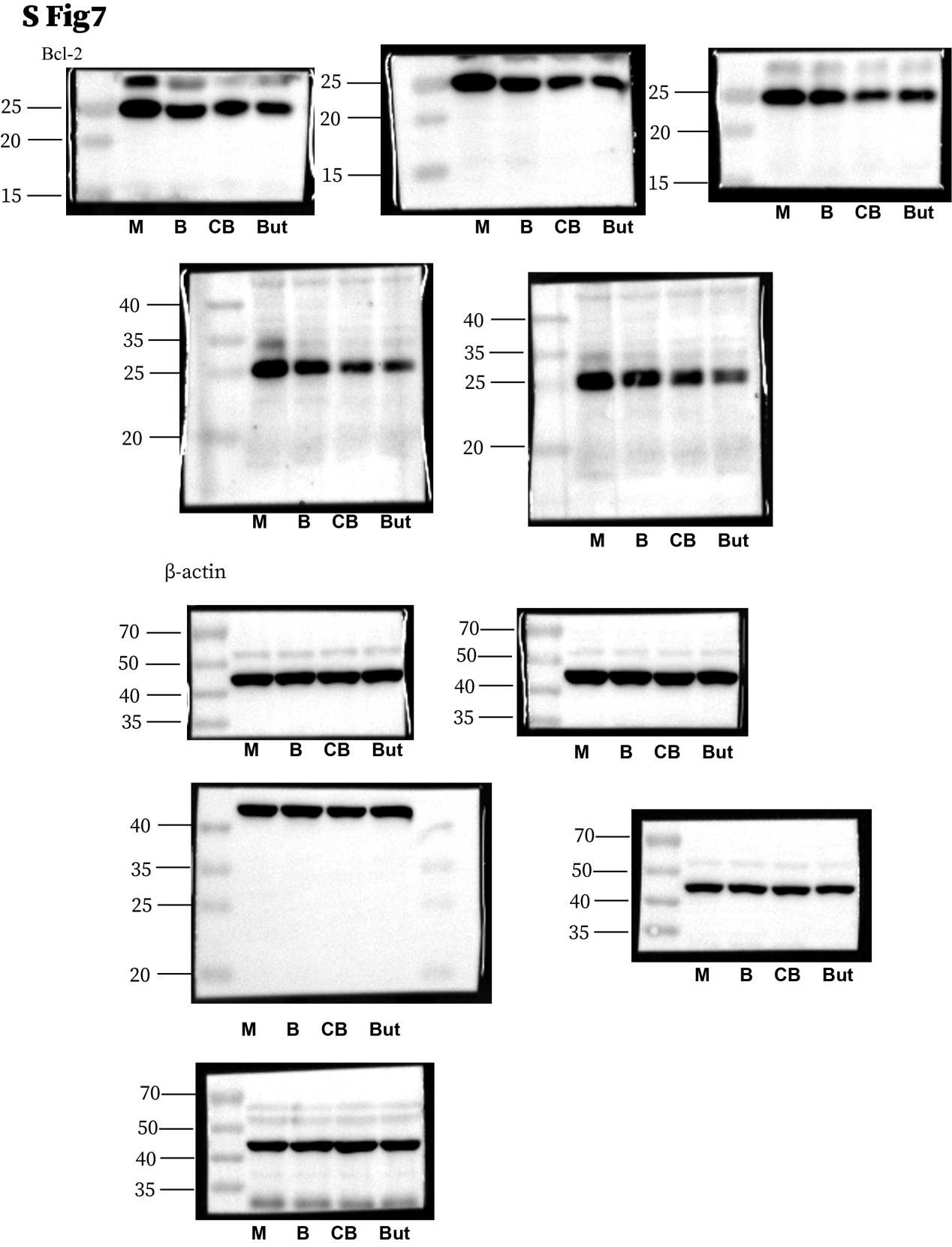


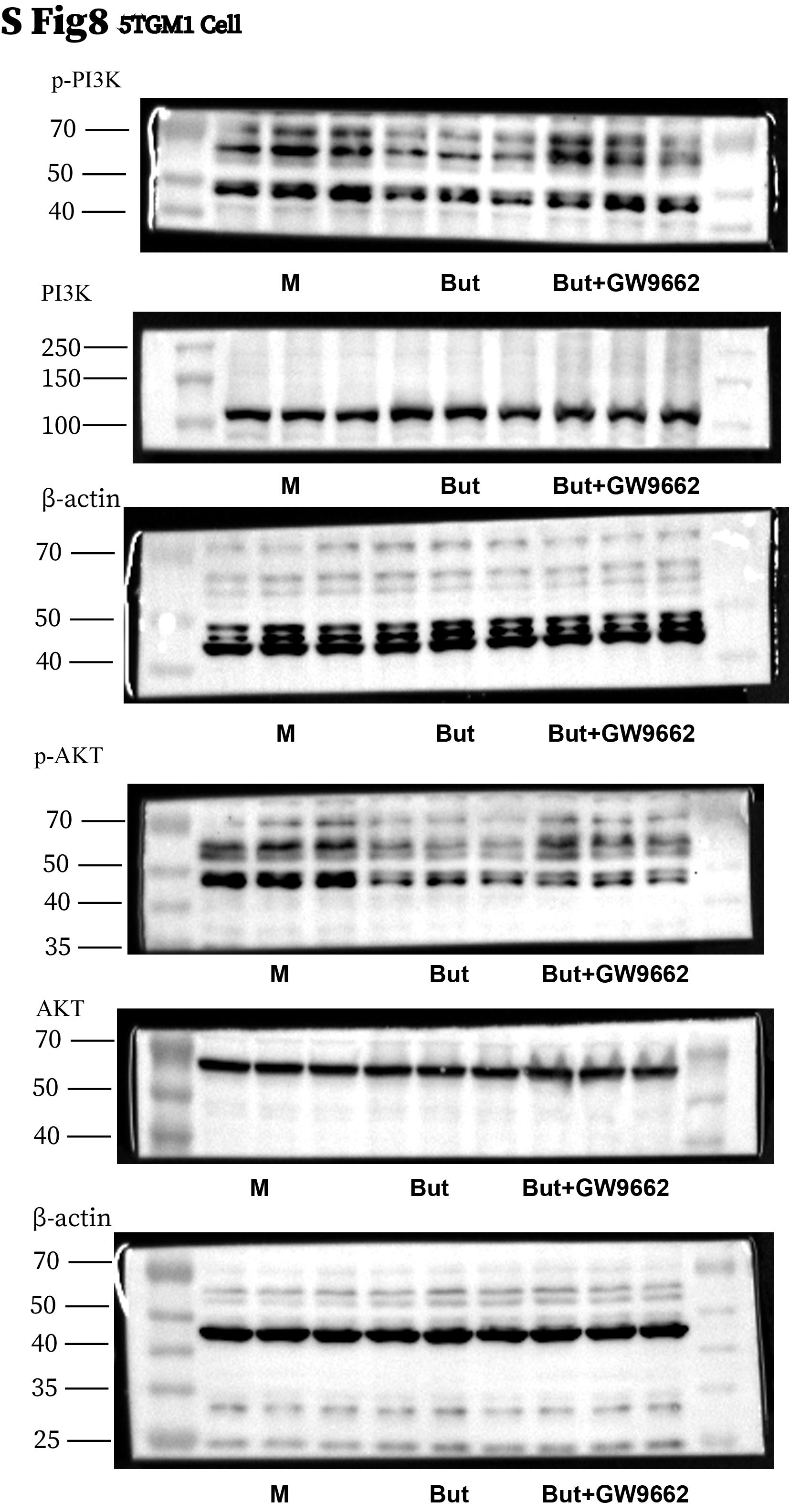


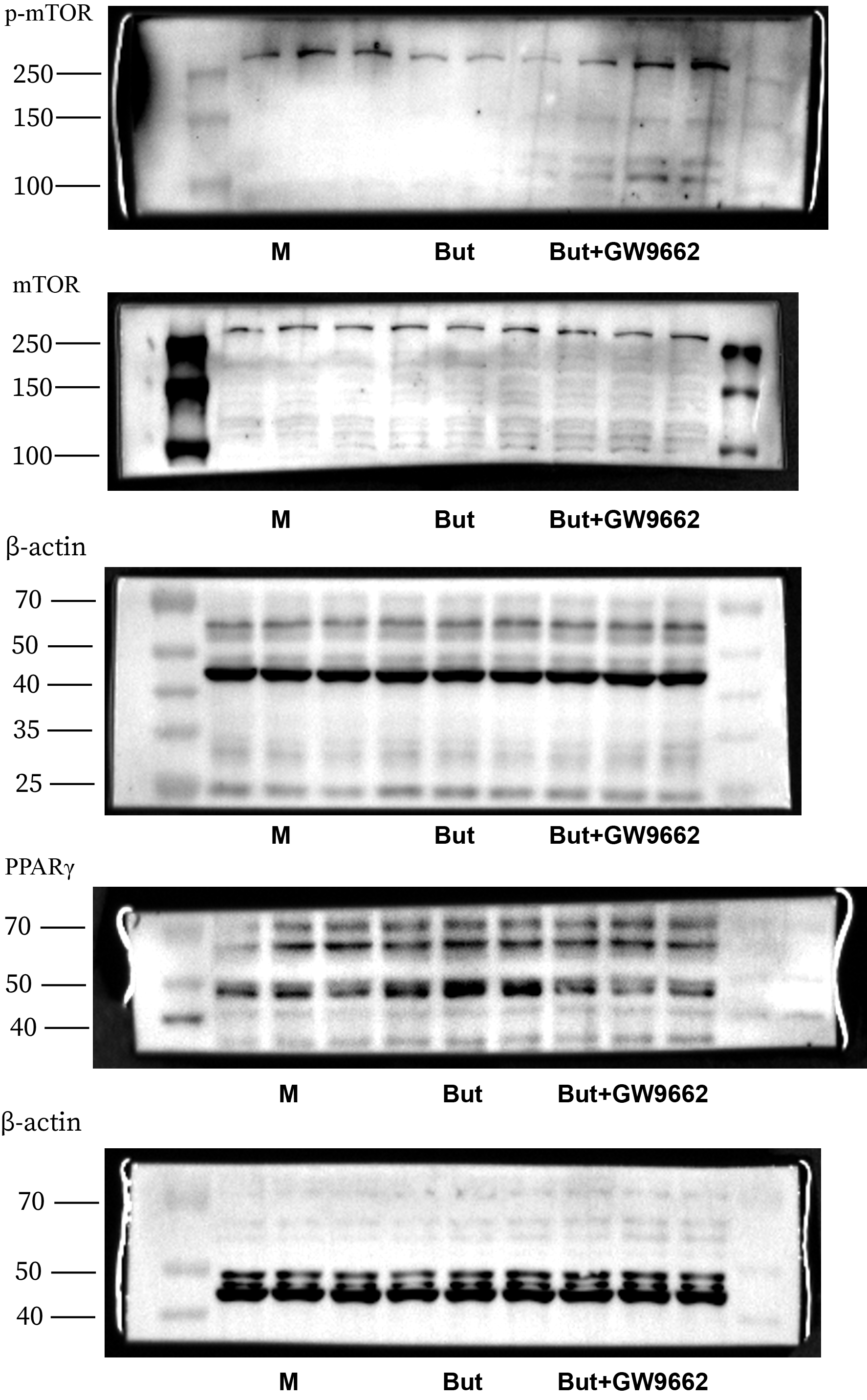


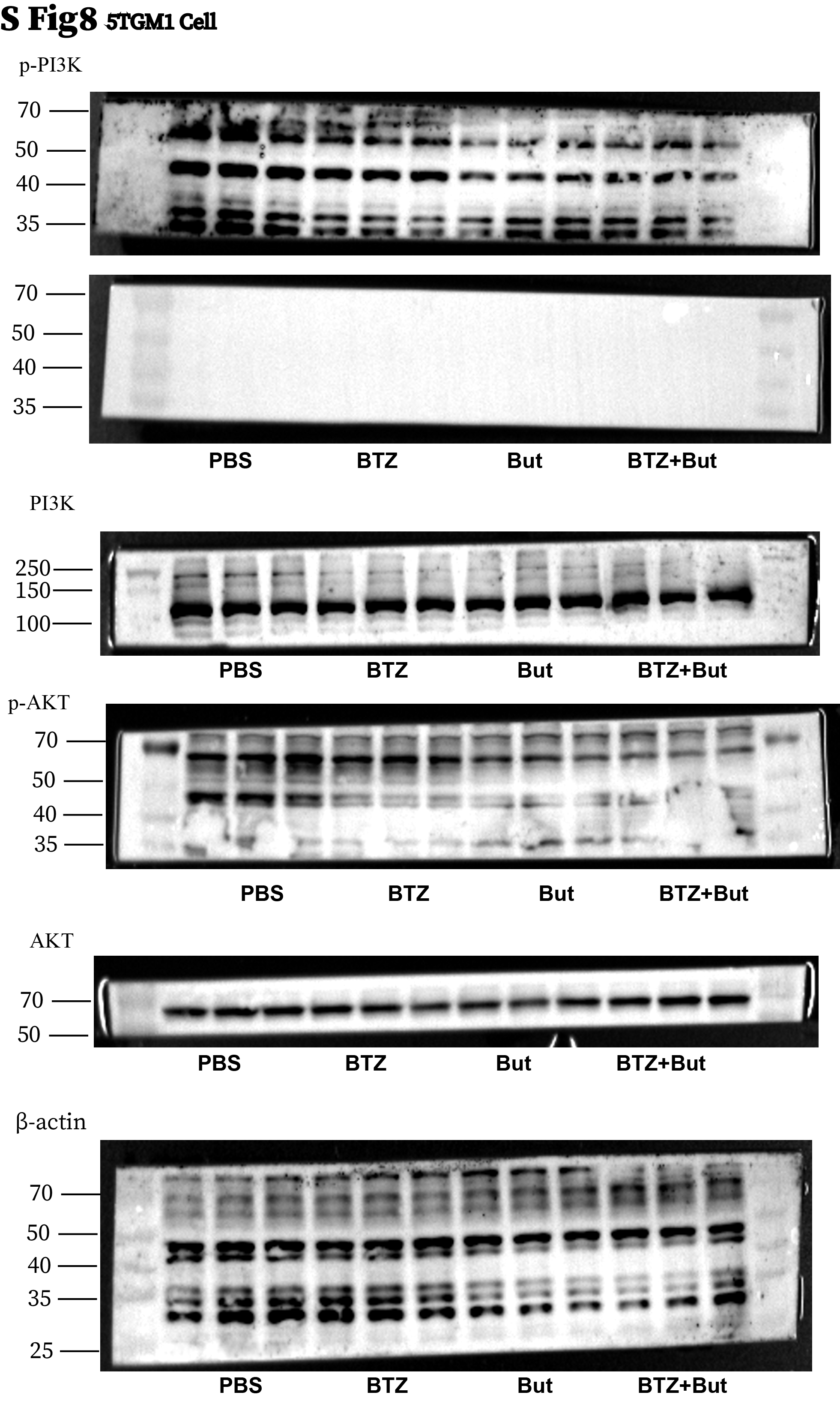

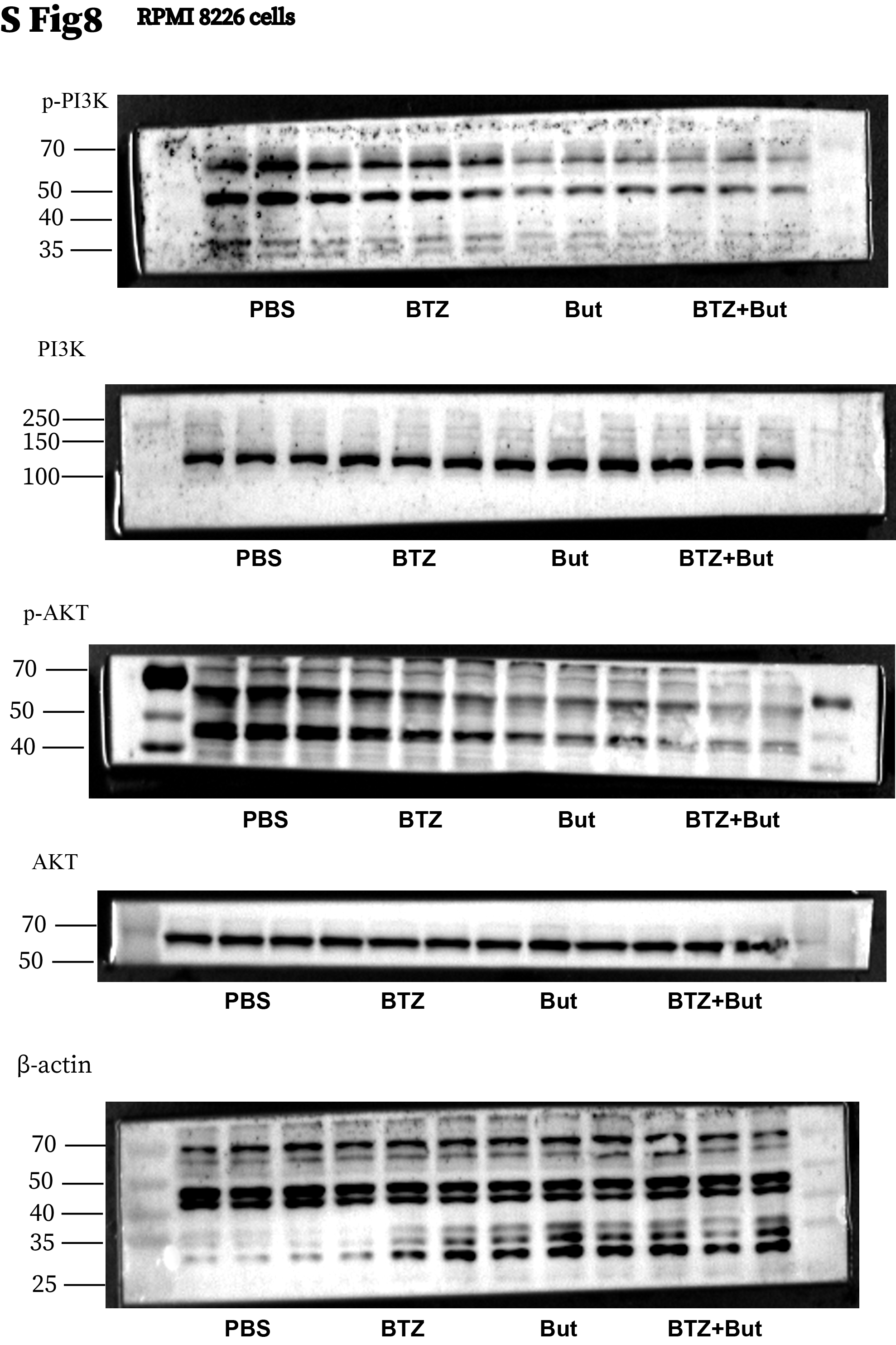

Supplement: Supplementary material — Supporting Information2.docx [file KGMI_A_2609455_SM8871.docx]
