# Supplementary material for: Clostridium butyricum alleviates multiple myeloma by remodeling the bone marrow microenvironment and inhibiting PI3K/AKT pathway through the gut‒bone axis
Source: Gut Microbes. 2026 Jan 2;18(1):2609455. doi: 10.1080/19490976.2025.2609455 (PMC12773645; doi:10.1080/19490976.2025.2609455)
Supplement: Supplementary material [file KGMI_A_2609455_SM3629.docx]

**Supplemental Table 1. Basic Characteristics of MM patients in the study.**

| Variables | MM without BD | MM with BD | *P* Value |
| --- | --- | --- | --- |
|  | N=15 | N=15 |  |
| Age(y) | 64.8±7.27 | 63.53±8.9 | 0.437 |
| Female, n (%) | 7 (46.7) | 11 (73.3) | 0.264 |
| BMI (kg/m^2^) | 22.37±1.66 | 22.22±3.88 | 0.051 |
| Pain/fracture, n (%) | 5(33.3) | 11(73.3) | 0.026 |
| DS staging, n (%) | 10(66.6) | 14(93.3) | 0.171 |
| ISS staging, n (%) | 11(73.3) | 11(73.3) | 1.000 |
| R-ISS staging, n (%) | 5(33.3) | 3 (20) | 0.680 |
| Plasma cells in bone marrow, n (%) | 34.5(21-59) | 31.5(23-44.5) | 0.740 |
| High-risk cytogenetics, n (%) | 8(53.3) | 5(33.3) | 0.462 |
| renal insufficiency, n (%) | 4(26.7) | 5(33.3) | 1.000 |
| WBC (10^9^/L) | 6.17±2.16 | 6.02±2.58 | 0.692 |
| HB (g/L) | 77.13±24.45 | 75.6±18 | 0.217 |
| PLT (10^9^/L) | 124(105-163) | 158(98-268) | 0.254 |
| LDH (U/L) | 196.7(162.24-294.3) | 165.09(142.59-212.95) | 0.165 |
| β2-MG (mg/L) | 7.28±3.17 | 7.23±2.49 | 0.392 |
| Ca^2+^(mmol/L) | 2.43(2.32-2.81) | 2.49(2.29-2.84) | 0.950 |
| Albumin (g/L) | 31.36±5 | 31.51±7.55 | 0.086 |
| BGP (ng/mL) | 21.16(17.87-40.83) | 26.37(13.26-57.37) | 0.787 |
| 25(OH)D (ng/mL) | 19.73±11.79 | 22.48±8.85 | 0.394 |
| PTH (pg/mL) | 41.73(16.12-63.06) | 16.88(10.34-175.7) | 0.576 |
| BALP (U/L) | 75.53±17.63 | 78.67±22.64 | 0.447 |
| β-CTX (pg/mL) | 561.9(390.2-853.2) | 1456(966.3-2514) | 0.001 |
| PINP (ng/mL) | 70.49(53.96-97.11) | 82.69(56.5-122.8) | 0.468 |
| Induction therapy, n (%) |  |  | 0.689 |
| Vcd | 4(26.7) | 5(33.3) | 0.341 |
| Vrd | 8(53.3) | 6(40.0) | 0.001 |
| Vd | 3(20.0) | 4(26.7) | 0.912 |
| Monoclonal protein type |  |  | 0.607 |

The continuous variables of normal distribution are expressed as mean ± standard deviation (SD), the variables of non-normal distribution are expressed as median (interquartile range), and the categorical variables are expressed as numbers and percentages. Independent sample chi-square (χ^2^) test or Mann-Whitney U test was used for non-normally distributed data, and non-paired t-test was used for normally distributed data to compare differences between groups. All tests were statistically significant at bilateral *P*<0.05. BD: Bone disease; BMI: Body mass index; WBC: White blood cell; HB: Hemoglobin; PLT: Platelet count; LDH: Lactate dehydrogenase; β2-MG: β2-microglobulin; BGP: Bone glaprotein; 25(OH)D: 25-hydroxyvitamin D; PTH: Parathyroid hormone; Vcd: Bortezomib/ cyclophosphamide/ dexamethasone; Vrd: Bortezomib/ lenalidomide/ dexamethasone.

**Supplemental Figure**


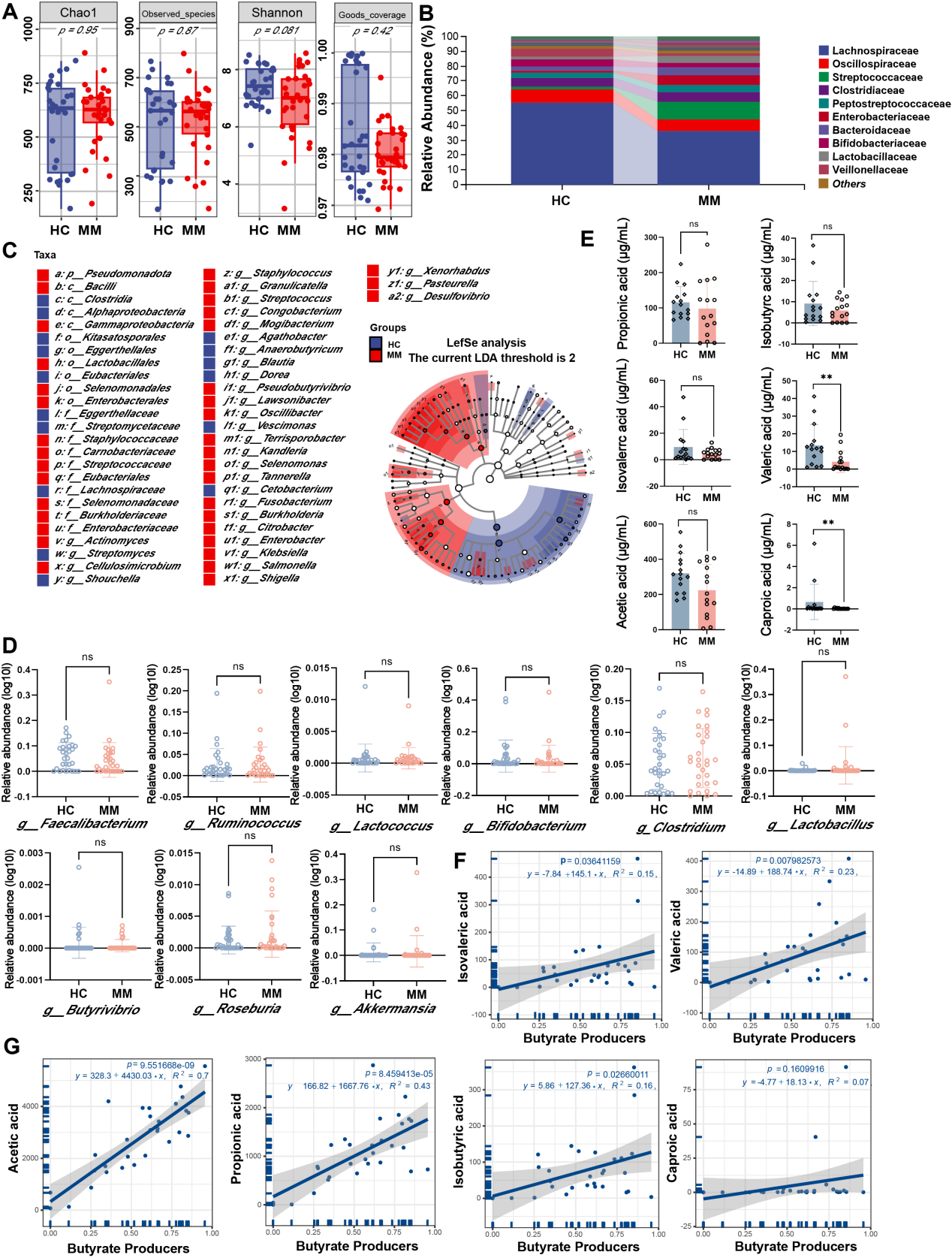


**Figure S1. A)** Alpha diversity of the microbial community. **B)** Family-level classification histogram. **C)** Classification branch graph of Linear discriminant analysis effect size showing the relative abundance of bacteria. **D)** Relative abundance of butyrate-producing bacteria (mean ± SEM, n=15, ***P* <0.01 by Mann-Whitney U test). **E)** Short-chain fatty acids (SCFAs) levels in feces. **F)** and **G)** Linear correlation between butyrate-producing bacteria abundance and SCFAs levels.


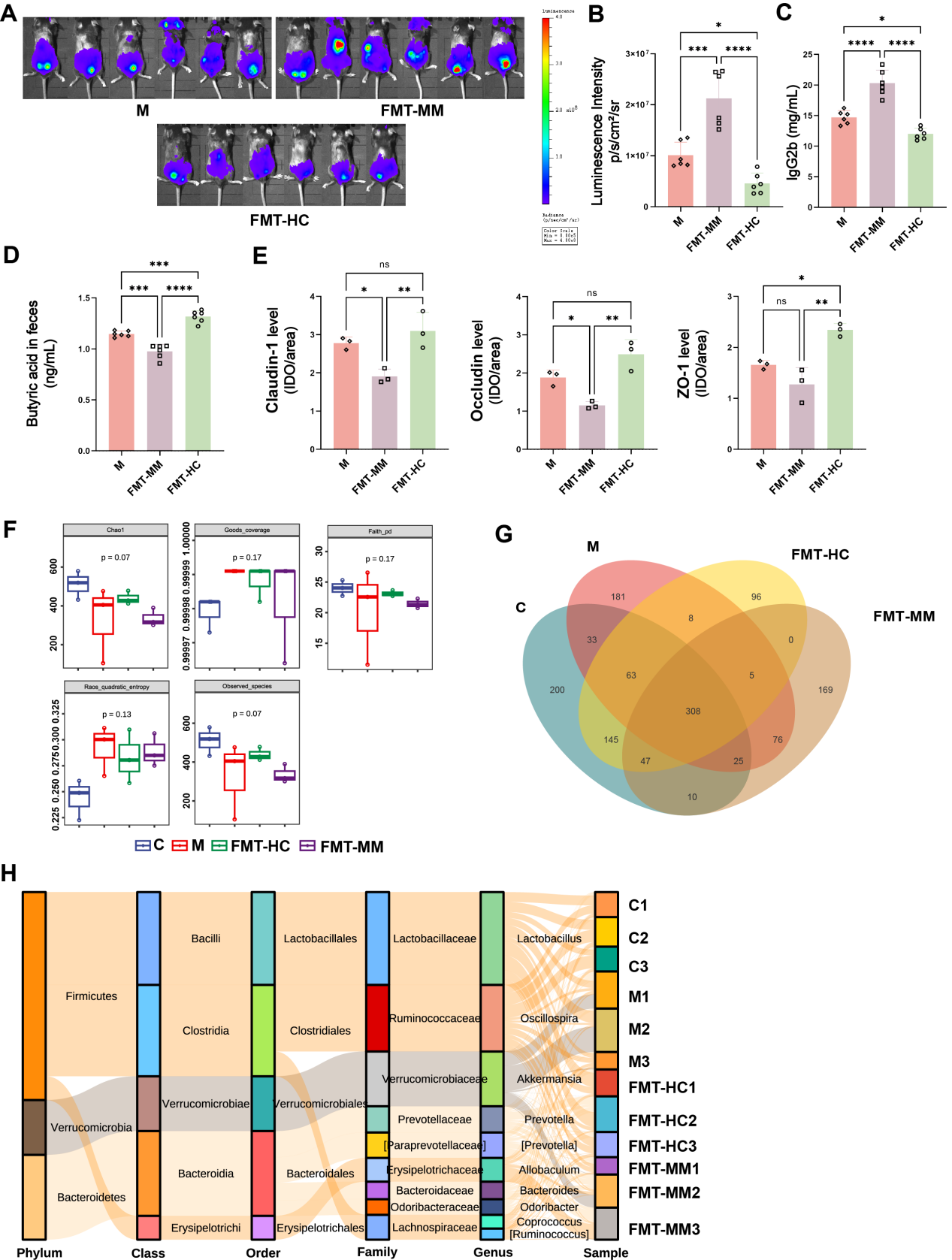


**Figure S2. A)** Live imaging of mice in the repeated FMT experiments. **B)** Quantification of fluorescence intensity (mean ± SEM, n=6, **P* <0.05, ****P* <0.001, *****P* <0.0001 by one-way ANOVA). **C)** Serum IgG2b concentrations of mice in the repeated FMT experiments (mean ± SEM, n=6, **P* <0.05, *****P* <0.0001 by one-way ANOVA). **D)** Fecal butyric acid levels (mean ± SEM, n=6, ****P* <0.001 by one-way ANOVA). **E)** Quantification of ZO-1, Claudin-1 and Occludin immunofluorescence staining (mean ± SEM, n=3, *P <0.05, **P < 0.01 by one-way ANOVA). **F)** Alpha diversity. **G)** Wayne diagram analysis of the species classification unit. **H)** Species composition mulberry map.


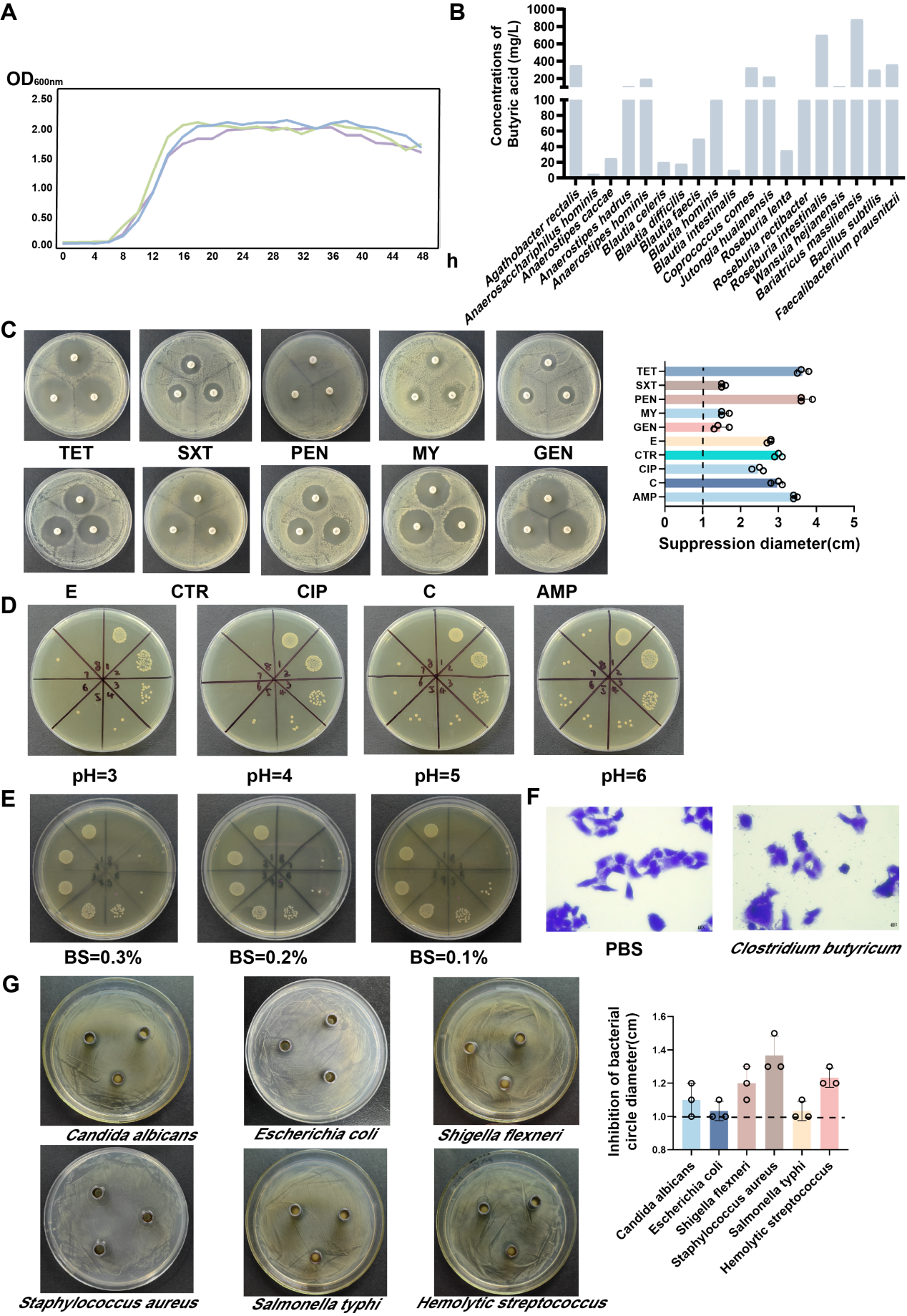


**Figure S3. A)** Growth curve of *Clostridium butyricum* (*C. butyricum*). **B)** The concentrations of butyric acid produced by several common butyric acid-producing bacteria *in vitro*. **C)** Antibiotic susceptibility test. The black dotted line represents the diameter of the drug-sensitive paper. **D)** The representative images of the acid resistance test. **E)** The representative images of the bile salt resistance experiment. **F)** Co-culture experiment of *C. butyricum* with HT29 cells. **G)** Antibacterial experiment. The black dotted line represents the diameter of the Oxford Cup. n=3 group. C: chloramphenicol (30μg/tablet), CTR: ceftriaxone (30μg/tablet), TET: tetracycline (30μg/tablet), GEN: gentamycin (10μg/tablet), PEN: penicillin (10μg/tablet), AMP: ampicillin (10μg/tablet), MY: Lincomycin (2μg/tablet), CIP: ciprofloxacin (5μg/tablet), SXT: Compound sulfamethoxazole (25μg/tablet), E: erythromycin (15μg/tablet).


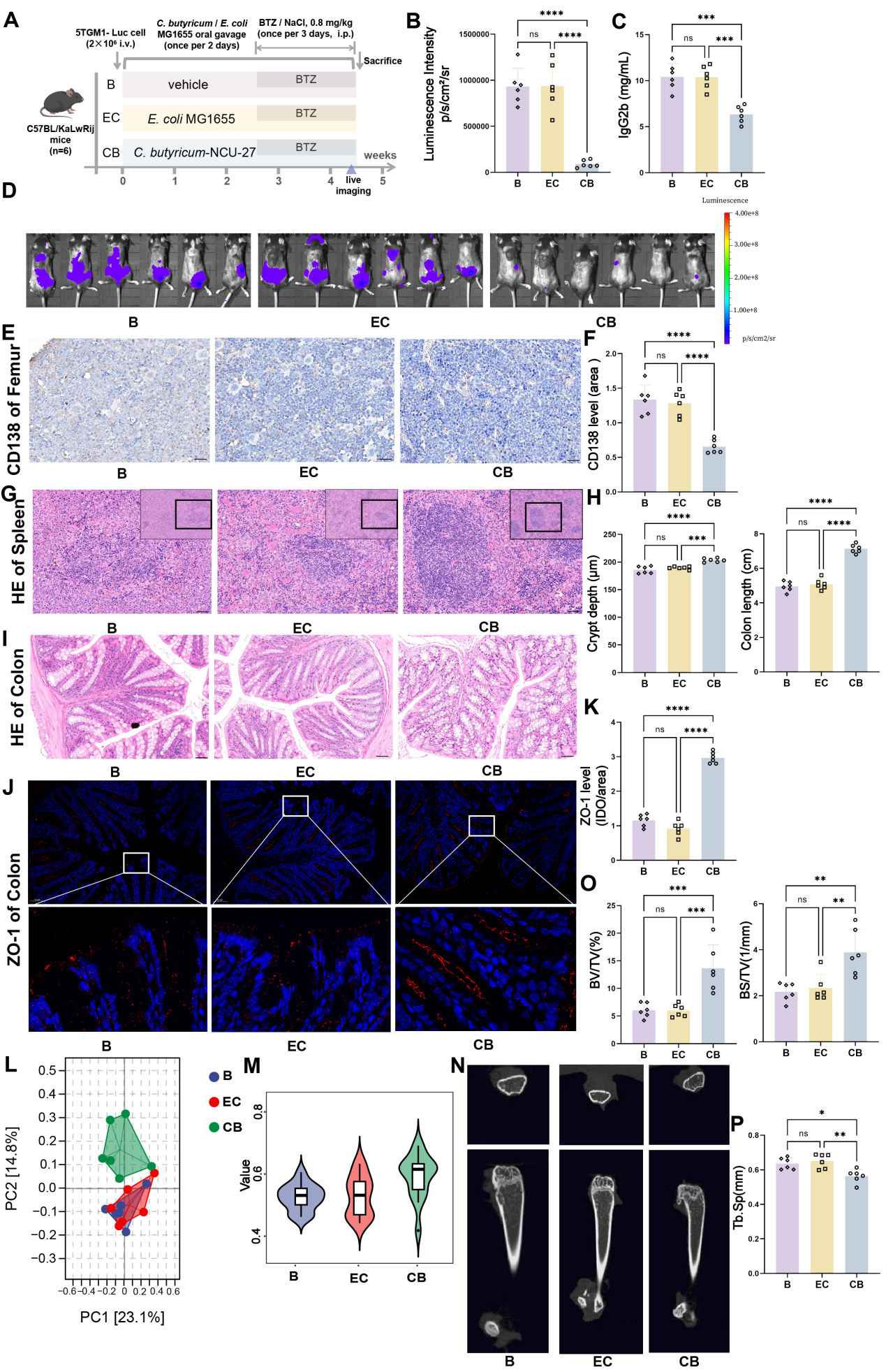


**Figure S4. A)** Experimental design schematic diagram. **B)** Quantification of fluorescence intensity in live imaging (mean ± SEM, n=6, *****P* <0.0001 by one-way ANOVA). **C)** Concentration of serum IgG2b (mean ± SEM, n=6, ****P* < 0.001 by one-way ANOVA). **D)** Live imaging of the mice at day 30. **E)** Immunohistochemistry of CD138 in femur, Scale bar: 25 μm. **F)** Quantification of CD138 immunohistochemical staining (mean ± SEM, n=6, *****P* <0.0001 by one-way ANOVA). **G)** The representative H&E staining of the spleen, Scale bar: 100 μm. **H)** Colonic crypt depth and colon length (mean ± SEM, n=6, ****P* < 0.001, *****P* <0.0001 by one-way ANOVA). **I)** The representative images of H&E staining of the colon, Scale bar: 50 μm. **J)** The representative images of immunofluorescence staining for ZO-1, Scale bar: 50 μm. **K)** Quantification of ZO-1 immunofluorescence staining (mean ± SEM, n=6, **P* <0.05, ***P* < 0.01 by one-way ANOVA). **L)** and **M)** PCoA analysis and comparative violin plots of Beta diversity. **N)** Representative micro-CT images of femur. **O)** and **P)** Quantitative results of BV/TV, BS/TV, Tb.Sp (mean ± SEM, n=6, **P* < 0.05, ***P* < 0.01, ****P* < 0.001 by one-way ANOVA).


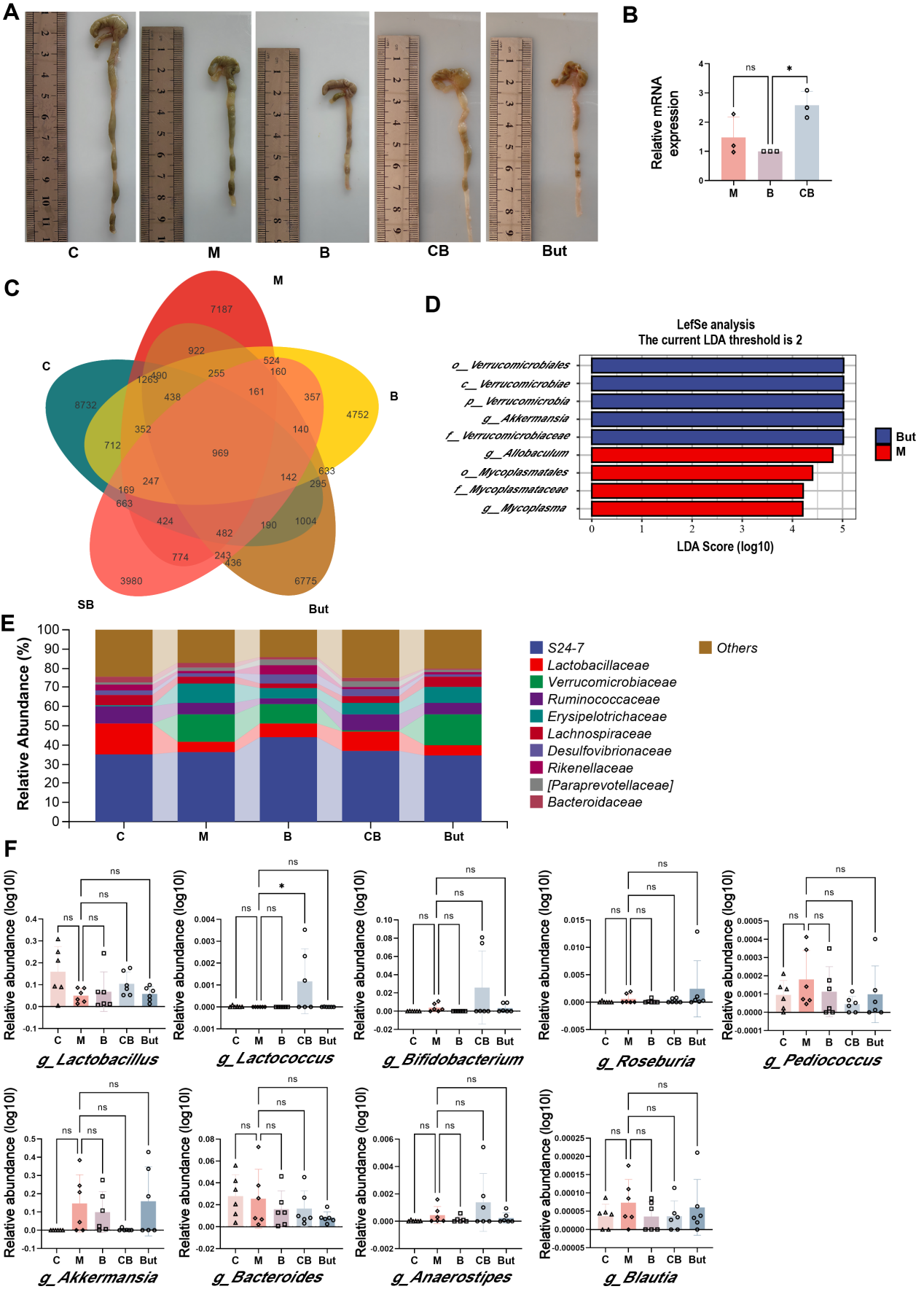


**Figure S5. A)** Representative images of the colon. **B)** Detection of *C. butyricum* by qPCR (mean ± SEM, n=3, **P* < 0.05 by one-way ANOVA). **C)** Wayne diagram analysis of species classification units. **D)** Histogram of Linear discriminant analysis effect size showing the relative abundance of bacteria. **E)** Family-level classification histogram. **F)** Relative abundance of several butyrate-producing bacteria (mean ± SEM, n=6, **P* < 0.05 by one-way ANOVA).


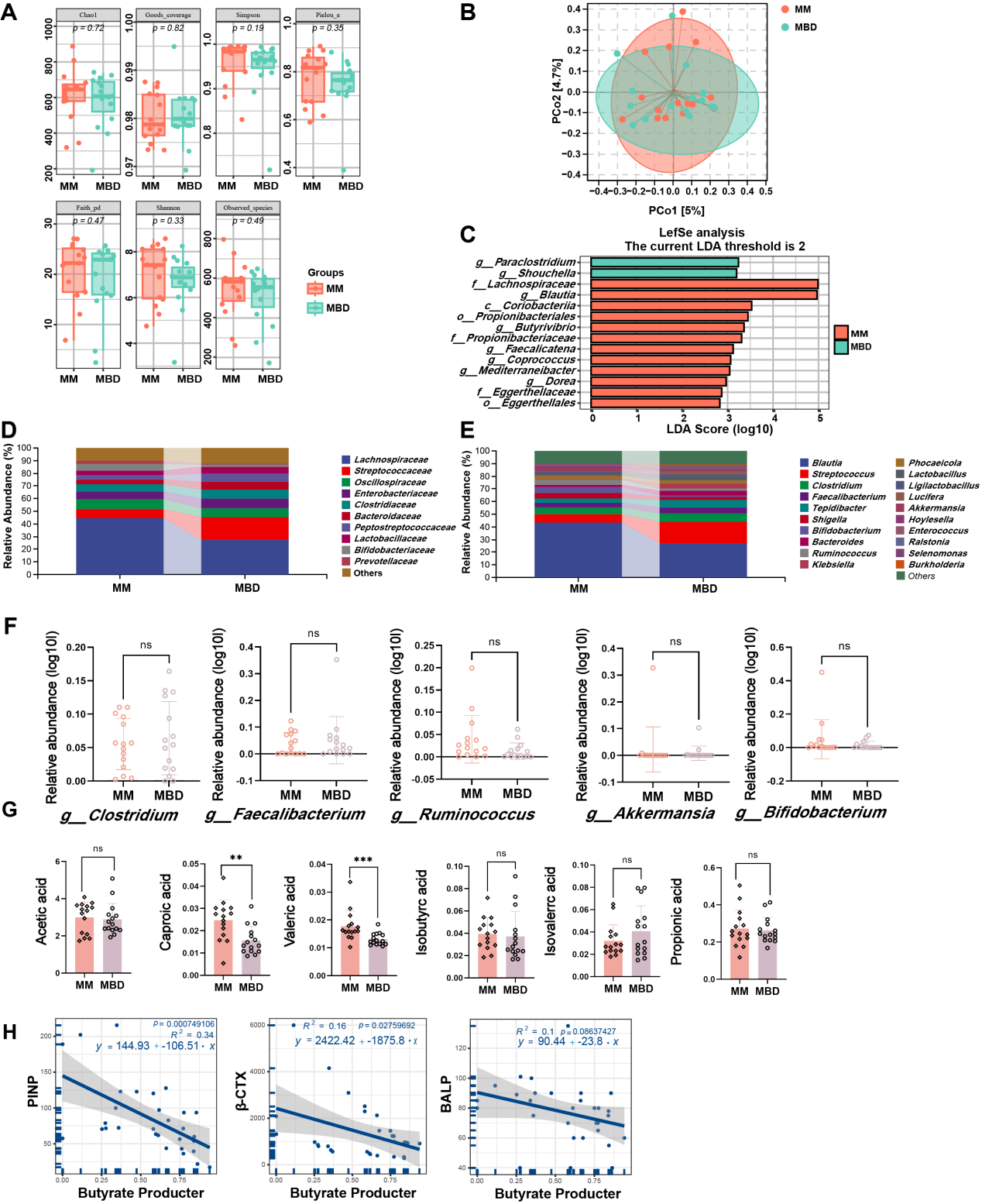


**Figure S6-1. A)** Alpha diversity of the microbial community in multiple myeloma patients with or without bone disease. **B)** Beta diversity. **C)** Histogram of Linear discriminant analysis effect size showing the relative abundance of bacteria. **D)** Family-level classification histogram. **E)** Genus-level classification histogram. **F)** Relative abundance of butyrate-producing bacteria. **G)** Short-chain fatty acids levels in serum (mean ± SEM, n=15, ***P* <0.01, ****P* < 0.001 by Mann-Whitney U test). **H)** linear correlation diagram between butyrate-producing bacteria and clinical bone metabolism markers.


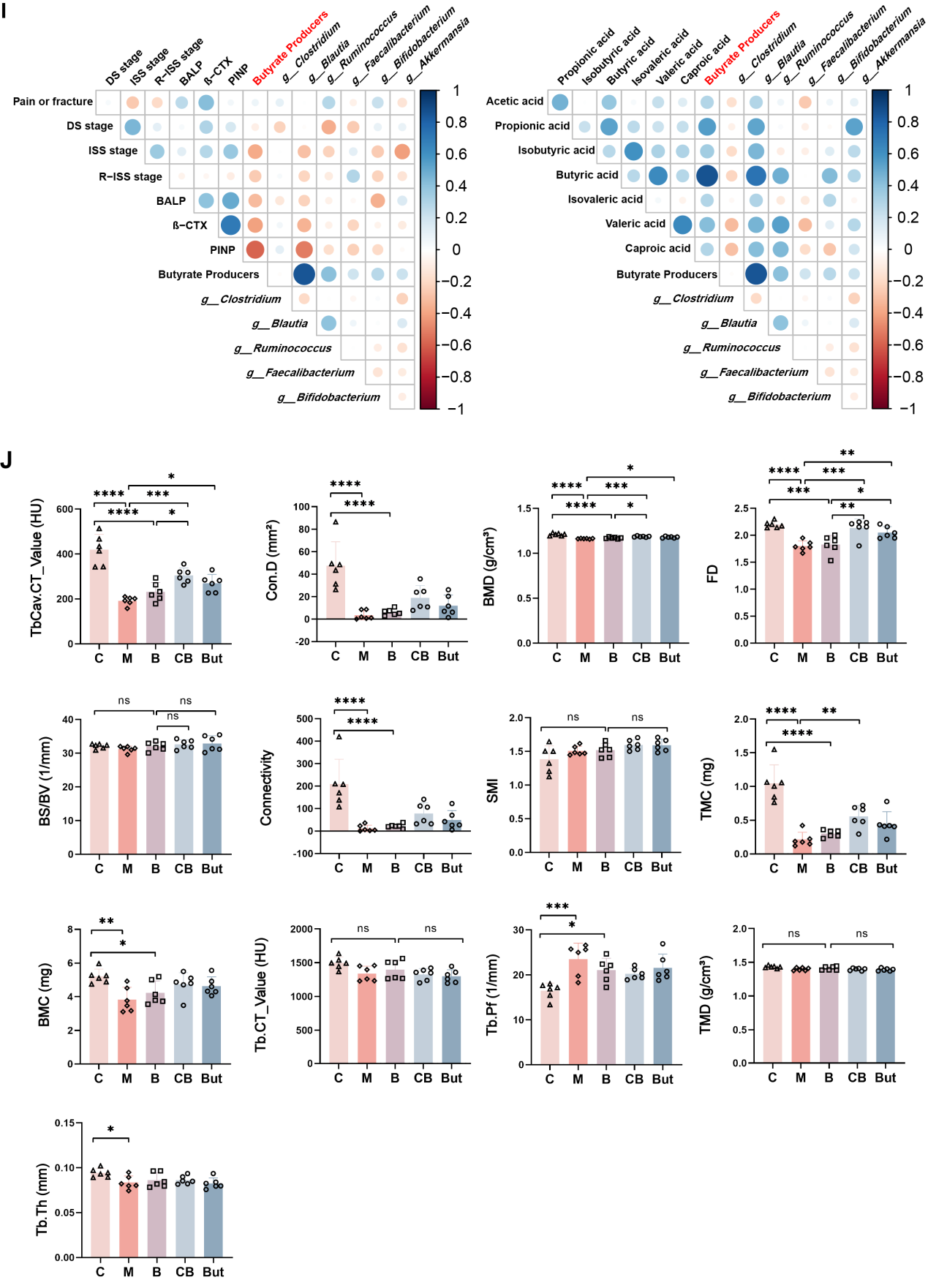


**Figure S6-2. I)** Correlation heat map of clinical indicators, short-chain fatty acids levels, and butyrate-producing bacteria abundance. **J)** Quantitative results of bone parameters (mean ± SEM, n=6, **P* < 0.05, ***P* < 0.01, ****P* < 0.001, *****P* <0.0001 by one-way ANOVA). TbCav.CT_Value: CT value of bone marrow cavity; Conn.D: Bone trabecular connection density; BMD: Bone mineral density; FD: Fractal dimension; BS/BV: Bone surface area and bone volume ratio; SMI: Structural model index; TMC: Tissue mineral content; BMC: Bone mineral content; Tb.CT_Value: CT value of trabecular bone; Tb.Pf: CT value of trabecular bone; TMD: Tissue mineral density; Tb.Th: trabecular thickness.


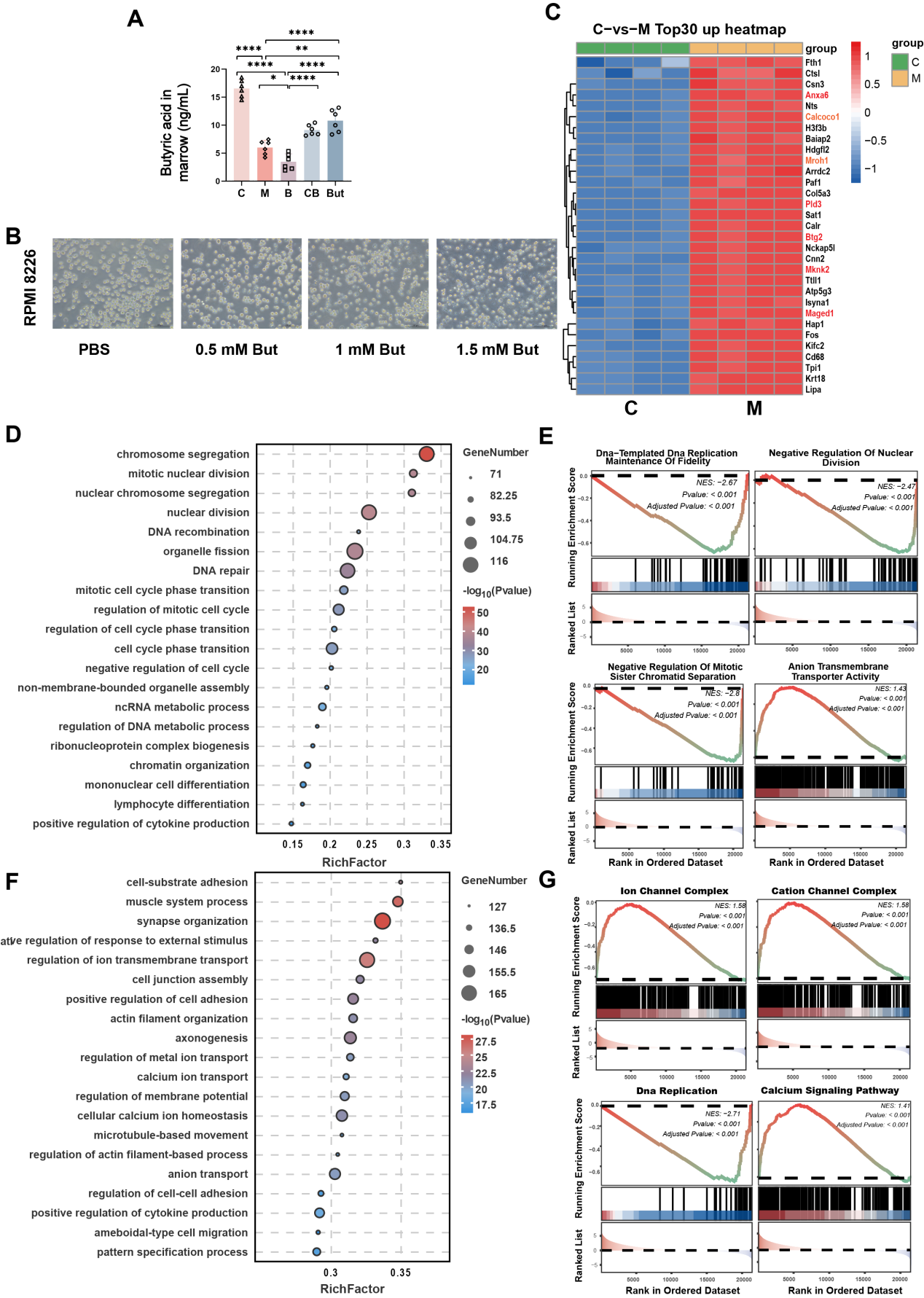


**Figure S7. A)** Butyric acid content in bone marrow (mean ± SEM, n=6, **P* < 0.05, ***P* < 0.01, *****P* <0.0001 by one-way ANOVA). **B)** Representative optical microscope images of RPMI 8226 cells treated with PBS or sodium butyrate. Scale bar: 100 μm. **C)** Heatmap of differentially upregulated genes in 5TGM1 cells identified via RNA-Seq analysis. **D)** GO enrichment analysis bubble diagram of 5TGM1 cells. **E)** GSEA enrichment analysis of biological processes. **F)** KEGG analysis bubble diagram in 5TGM1 cells. **G)** GSEA enrichment analysis of molecular function.


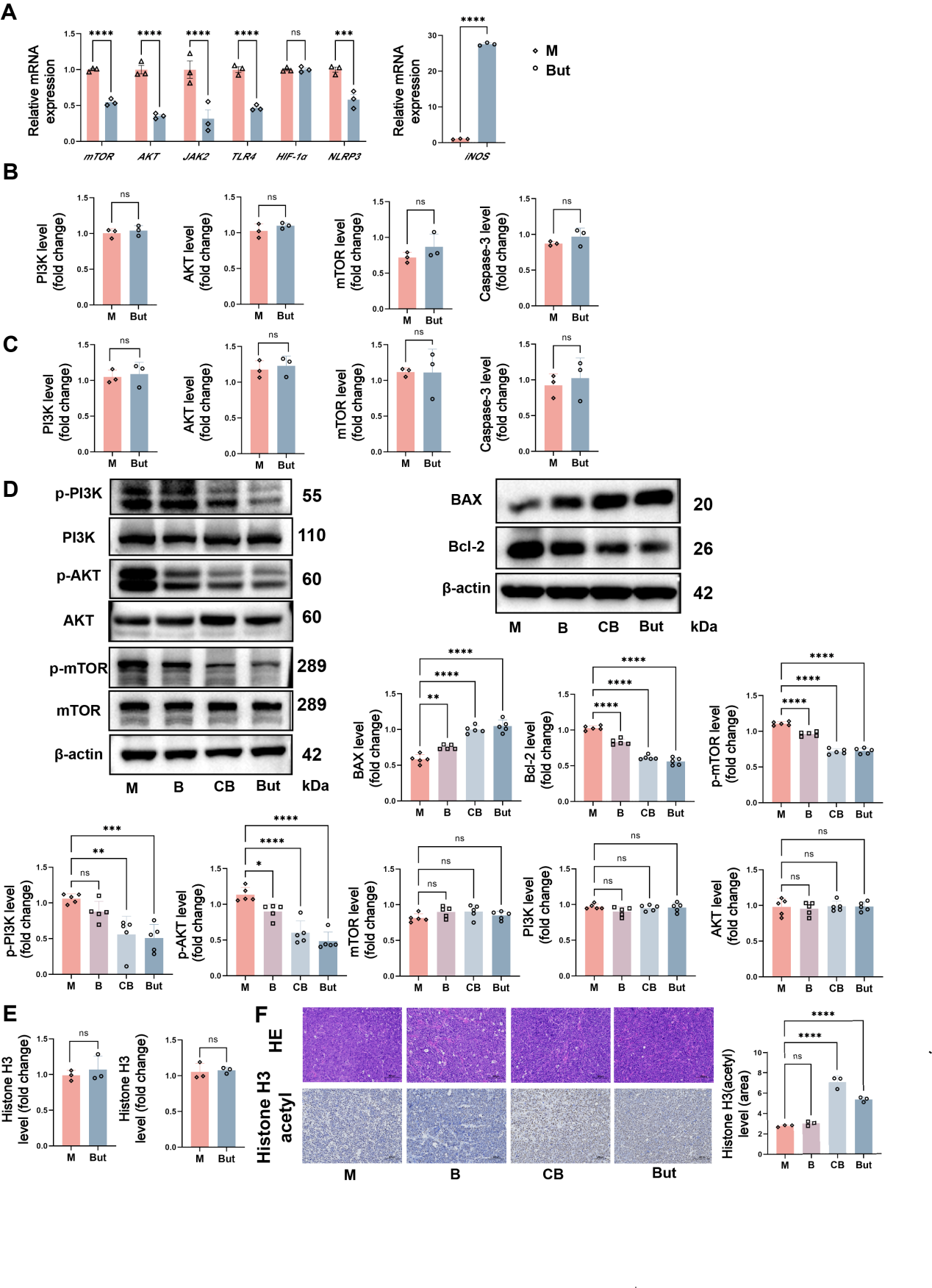


**Figure S8. A)** The gene expression levels tested by PCR in 5TGM1 cells (mean ± SEM, n=3, ****P* < 0.001, *****P* <0.0001 by Mann-Whitney U test). **B)** and **C)** Quantification of PI3K, AKT, mTOR, and Caspase3 protein levels in **B)** 5TGM1 cells and **C)** RPMI 8226 cells. **D)** Western blot analysis and quantification of expression levels for PI3K/AKT/mTOR pathway and apoptosis proteins in the tumor tissues. (mean ± SEM, n=5, **P* < 0.05, ***P* < 0.01, ****P* < 0.001, *****P* <0.0001 by Mann-Whitney U test). **E)** Quantification of acetylated histone levels in 5TGM1 cells and RPMI 8226 cells. **F)** The representative images of tumor H&E staining, immunohistochemistry, and quantitative results of tumor acetylated histone, Scale bar: 100 μm (mean ± SEM, n=3, *****P* <0.0001 by one-way ANOVA).


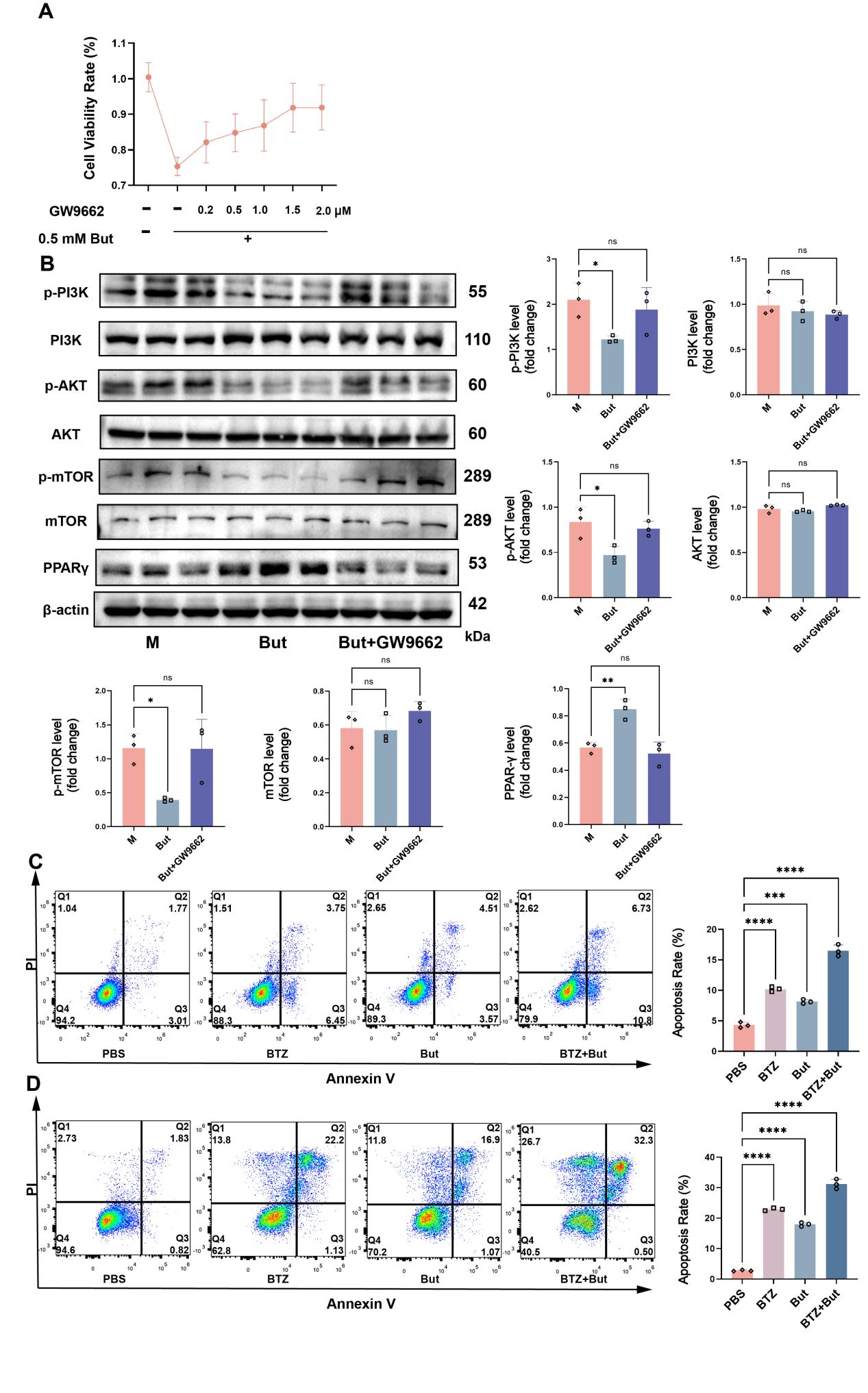


**Figure S9-1. A)** Cell viability of 5TGM1 cells assessed by Cell Counting Kit-8 in response to sodium butyrate and PPARγ inhibitor GW9662 at diverse concentrations (mean ± SEM, n=6). **B)** Western blot analysis showing changes in the PI3K/AKT/mTOR signaling pathway and PPARγ protein expression levels in 5TGM1 cells treated with vehicle, sodium butyrate (But), or But combined with GW9662 (mean ± SEM, n=3, **P* < 0.05, ***P* < 0.01, ****P* < 0.001, *****P* <0.0001 by Mann-Whitney U test). **C)** Representative flow cytometry plots and quantification of apoptotic 5TGM1 cells after treatment with PBS, bortezomib (BTZ), sodium butyrate (But), or BTZ combined with But. **D)** Representative flow cytometry plots and quantification of apoptotic RPMI 8226 cells after treatment with PBS, BTZ, But, or BTZ combined with But (mean ± SEM, n=3, **P* < 0.05, ***P* < 0.01, *****P* <0.0001 by one-way ANOVA).


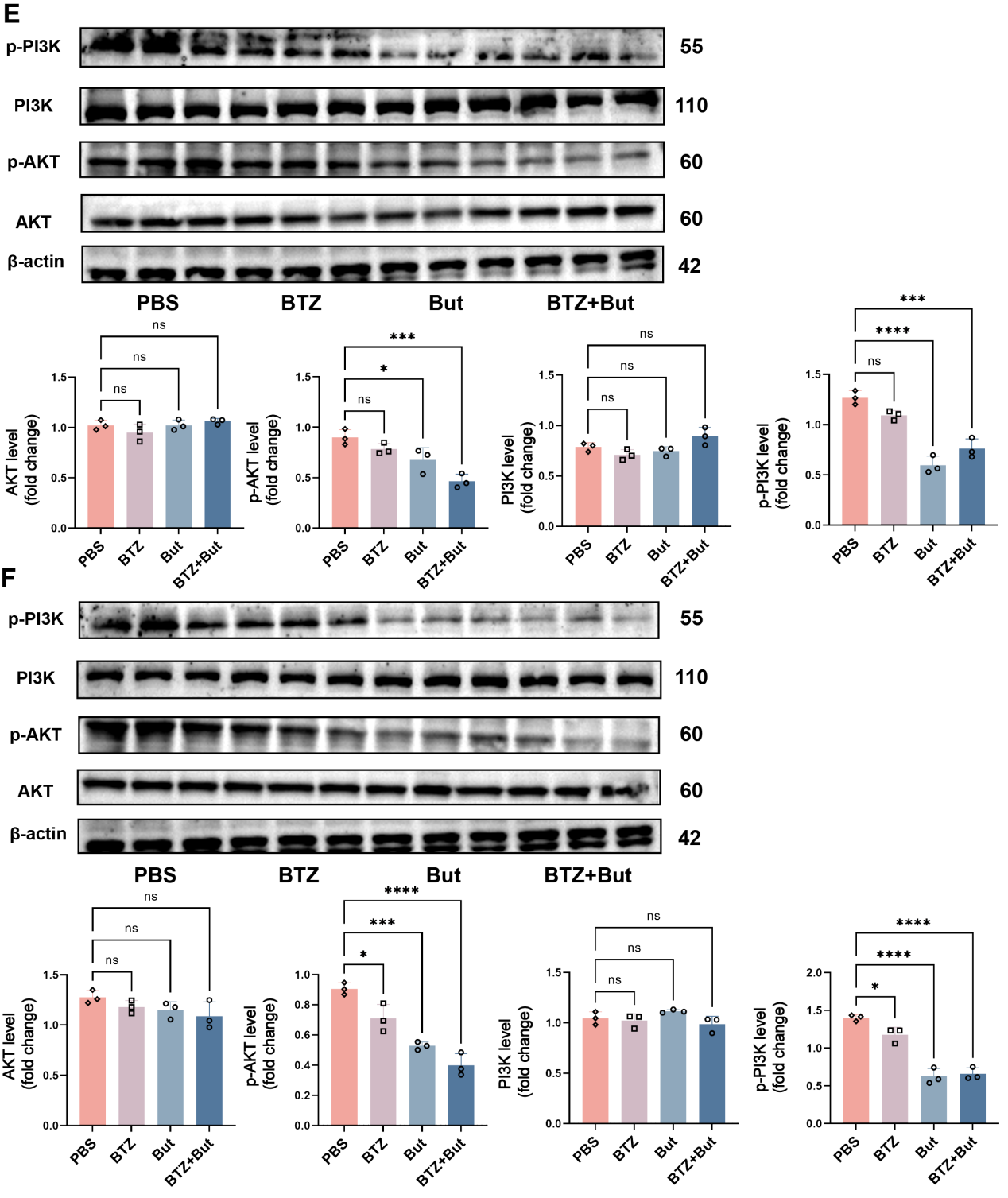


**Figure S9-2. E)** Western blot analysis and quantification of expression levels for PI3K/AKT pathway in 5TGM1 cells after treatment with PBS, BTZ, But, or BTZ combined with But. (mean ± SEM, n=3, **P* < 0.05, ***P* < 0.01, ****P* < 0.001, *****P* <0.0001 by Mann-Whitney U test). **F)** Western blot analysis and quantification of expression levels for PI3K/AKT pathway in RPMI 8226 cells after treatment with PBS, BTZ, But, or BTZ combined with But. (mean ± SEM, n=3, **P* < 0.05, ***P* < 0.01, ****P* < 0.001, *****P* <0.0001 by Mann-Whitney U test).


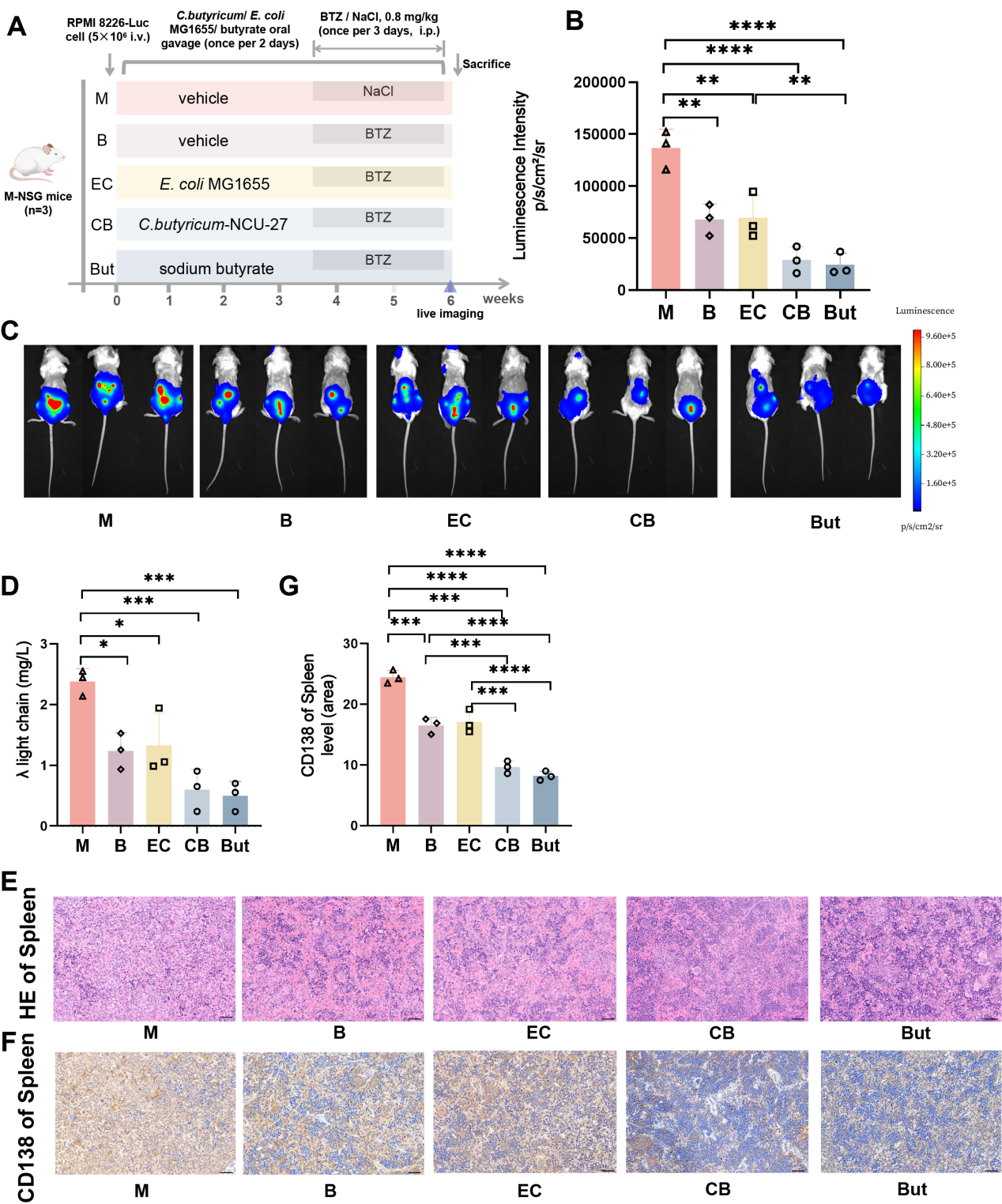


**Figure S10. A)** Experimental design schematic diagram. **B)** Quantification of fluorescence intensity in live imaging of the M-NSG mice (mean ± SEM, n=3, ***P* < 0.01, *****P* <0.0001 by one-way ANOVA). **C)** Live imaging of the M-NSG mice. **D)** Serum immunoglobulin λ light chain levels of the M-NSG mice (mean ± SEM, n=3, **P* <0.05, ****P* < 0.001 by one-way ANOVA). **E)** The spleen representative H&E staining of the M-NSG mice, Scale bar: 100 μm. **F)** The spleen representative CD138 immunohistochemistry of the M-NSG mice, Scale bar: 100 μm. **G)** Quantification of CD138 immunohistochemical staining (mean ± SEM, n=3, ****P* < 0.001, *****P* <0.0001 by one-way ANOVA).
